# DRP1 and MID49 co-diffusion scans mitochondria for fission

**DOI:** 10.1038/s41556-026-01986-w

**Published:** 2026-06-11

**Authors:** Cristiana Zollo, David Gomez Suarez, Elmira Parvindokht Bararpour, Andreas Jenner, Pratima Verma, Jan Koch, Sabine Wilhelm, Christian Jüngst, Lukas Faber, Faye White, Ana J. García-Sáez

**Affiliations:** 1 Institute of Genetics, University of Cologne, Cologne, Germany; 2 Cologne Excellence Cluster on Cellular Stress Responses in Aging-Associated Diseases, University of Cologne, Cologne, Germany; 3 Department of Membrane Dynamics, Max Planck Institute of Biophysics, Frankfurt am Main, Germany; 4 Imaging Facility, Max Planck Institutes of Biophysics and Brain Research, Frankfurt am Main, Germany; 5 Cluster of Excellence SubCellular Architecture of Life (SCALE), Goethe University Frankfurt, Frankfurt, Germany

**Keywords:** Mitochondria, Single-molecule biophysics

## Abstract

DRP1 is a dynamin-related large GTPase responsible for mitochondrial fission, which ensures proper mitochondrial distribution, morphology and quality control. Despite its relevance, the mechanism of mitochondrial division, especially regarding the dynamic regulation of DRP1, remains elusive. Here we report that DRP1 oligomers diffuse in helical-like trajectories along mitochondria, browsing the organelle surface and stalling at preconstricted fission sites, in what we call ‘mito-scanner’ motion. Molecular dynamics simulations support a geometry-mediated diffusion mechanism emerging from surface confinement. Perturbation of DRP1 motility results in elongated mitochondria, underscoring the functional importance of DRP1 scanning dynamics in mitochondrial division. We also show that DRP1 dynamics on mitochondria are differentially regulated by interactions with its adaptors, where co-diffusion of MID49/MID51 with DRP1 promotes its motility. Our findings support a model in which receptor-regulated mitochondrial surveillance by DRP1 enables balanced organelle division, with potential implications for targeting this process in disease.

## Abstract (web summary)

Zollo et al. examine DRP1 behaviour at mitochondria: DRP1 diffuses along mitochondria in helical-like patterns influenced by MID49/MID51, scanning the organelle surface and stalling at preconstricted fission sites.

## Main

Mitochondrial fission is a vital process essential for maintaining mitochondrial function, distribution and the faithful inheritance of mitochondrial DNA during cell division^1^. It ensures proper segregation of mitochondria to daughter cells and contributes to mitochondrial network remodelling and quality control^2–5^. Mitochondrial fission requires the large GTPase dynamin-related protein 1 (DRP1)^6,7^, whose function is essential, as DRP1 knockout (KO) in mice results in embryonic lethality due to abnormal placental and cardiac development^8,9^. Dysregulated DRP1 activity has also been linked to tumorigenesis, cardiomyopathies and neurodegeneration^10–14^.

In mammalian cells, DRP1 shuttles between mitochondria and the cytosol under basal conditions and acts as a sensor of cellular stress and metabolic cues^7,15^. Upon activation, DRP1 is recruited to discrete foci on the mitochondrial outer membrane (MOM), where it assembles into oligomeric complexes that mediate membrane constriction and division^16–20^. For this, DRP1 assemblies undergo progressive maturation, with only a fraction of them resulting in mitochondrial fission for reasons incompletely understood. DRP1 domain architecture comprises an N-terminal GTPase domain that hydrolyses GTP to drive conformational changes, a central stalk that mediates oligomerization and self-assembly into higher-order structures, a bundle signalling element (BSE) that transmits conformational changes from the GTPase domain to the stalk, and a variable domain (VD) regulating membrane association and curvature sensing^21–27^.

DRP1 function is tightly regulated by recruitment to mitochondria through receptor proteins^28^ and by post-translational modifications including phosphorylation and SUMOylation^29–31^. These mechanisms regulate DRP1 localization and oligomerization. For example, phosphorylation of Ser616 and Ser637 promotes or inhibits mitochondrial fission depending cellular context^30,32,33^. Other modifications, such as ubiquitination and O-GlcNAcylation, as well as modifications of DRP1 receptors, also modulate this process^34^. While mitochondrial fission factor (MFF) and mitochondrial dynamics proteins of 49 and 51 kDa (MID49 and MID51) are bona fide DRP1 receptors, the role of mitochondrial fission protein 1 (FIS1) remains debated^35,36^. Genetic ablation of these receptors strongly impairs DRP1 recruitment, with MFF depletion being most severe^37,38^. Simultaneous loss of MFF, MID49 and MID51 fully phenocopies DRP1 KO, underscoring their non-redundant roles^19^. Mechanistically, MFF enhances DRP1’s GTPase activity, an effect further potentiated by cardiolipin^25^. Cryogenic electron microscopy structures of full-length DRP1 co-assembled with MID49 revealed that MID49 not only recruits DRP1 but also co-polymerizes into linear filaments, stabilizing specific nucleotide-bound states^24^. A recent study by Kleele et al.^39^ further highlighted how different receptors mediate fission events with spatially and functionally distinct outcomes: MFF drives midzone divisions linked to mitochondrial biogenesis, while FIS1 coordinates peripheral fission events that facilitate mitophagy. However, little is known about the dynamic behaviour of DRP1 at the MOM, its regulation by DRP1 adaptors and how it contributes to mitochondrial fission.

Here, we report that individual DRP1 oligomers freely diffuse along the mitochondrial membrane in helical-like trajectories that scan the organelle for fission sites, which we call ‘mito-scanning’. Supported by molecular dynamics simulations, we propose that this motion arises from coupling between DRP1 structure, membrane interactions and mitochondrial geometry. Remarkably, perturbation of DRP1 mobility compromises its ability to mediate fission. We also show that DRP1 scanning dynamics is regulated by direct interaction with MID49/MID51 (MID49/51), but not MFF or FIS1, which co-diffuses with DRP1 and promotes its mobility on the mitochondrial surface. Our findings reveal a mechanistic link between DRP1 dynamics, receptor interactions and the regulation of mitochondrial fission.

## Results

### DRP1 oligomers scan the surface of mitochondria with spiral-like trajectories

**Fig. 1:**
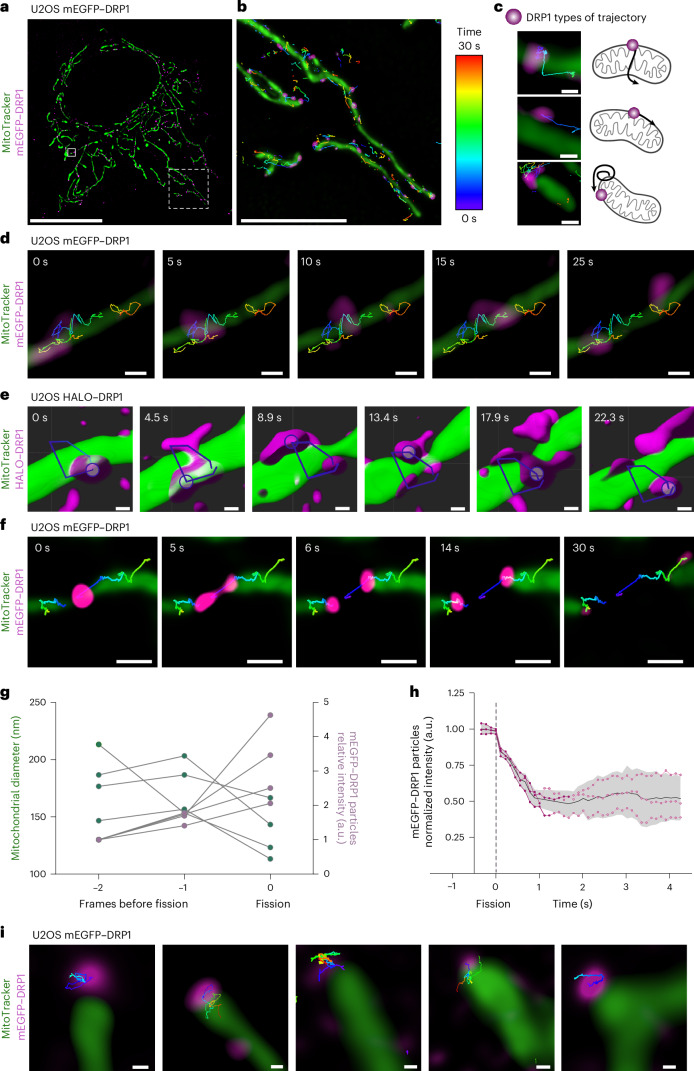
mEGFP–DRP1 oligomers scan the surface of mitochondria with spiral-like trajectories. **a**, Snapshot from a SIM video of a U2OS mEGFP–DRP1 cell showing mitochondria (green, labelled with MitoTracker Deep Red) and mEGFP–DRP1 (magenta). Scale bar, 20 µm. **b**, Zoomed-in view of the boxed region in **a**. mEGFP–DRP1 particle trajectories colour-coded by time. Scale bar, 500 nm. **c**, Right: schematic representation of DRP1 trajectories according to motion type. Left: SIM of mEGFP–DRP1. From top to bottom: spiral-like, along the longitudinal mitochondrial axis, and rotating at the mitochondrial tip. Scale bar, 0.2 µm. Representative of three independent experiments. **d**, Time-lapse SIM images of a mEGFP–DRP1 particle (magenta) trajectory on the mitochondrial tubule (green, labelled with MitoTracker Deep Red). Scale bars, 100 nm. **e**, A 3D live-cell SIM rendered time lapse showing HALO–DRP1 (magenta; labelled with JFX554) diffusing around mitochondria (green; labelled with MitoTracker Deep Red). Scale bars, 100 nm. **f**, Representative snapshot from a 2D SIM video of a U2OS mEGFP–DRP1 cell showing mEGFP–DRP1 (magenta) during mitochondrial fission (green, labelled with MitoTracker Deep Red). **g**, Mitochondrial diameter at fission sites was determined by FWHM analysis at the time of fission (0) and two frames preceding fission (−1 and −2). A total of four independent fission events were analysed. Individual dots represent single FWHM measurements extracted from line profiles across the mitochondrial constriction site. The right *y* axis shows the corresponding DRP1 intensity for each event. Only events with stable mitochondrial signal-to-noise ratio and focus throughout the acquisition were included. **h**, Quantification of mEGFP–DRP1 fluorescence intensity at mitochondrial constriction sites at the time of fission (0) and after fission. Fluorescence intensity was normalized to the prefission value (set to 1). Individual fluorescence intensity traces (*n* = 3 fission events) are shown. Mean (dotted line) ± s.d. (shaded area). **i**, Representative SIM images showing mEGFP–DRP1 particles (magenta) rotating at mitochondrial tips (green, labelled with MitoTracker Deep Red). Trajectory colour-coded by time. Scale bars, 0.2 µm. Representative of three independent experiments. Source data

**Extended Data Fig. 1:**
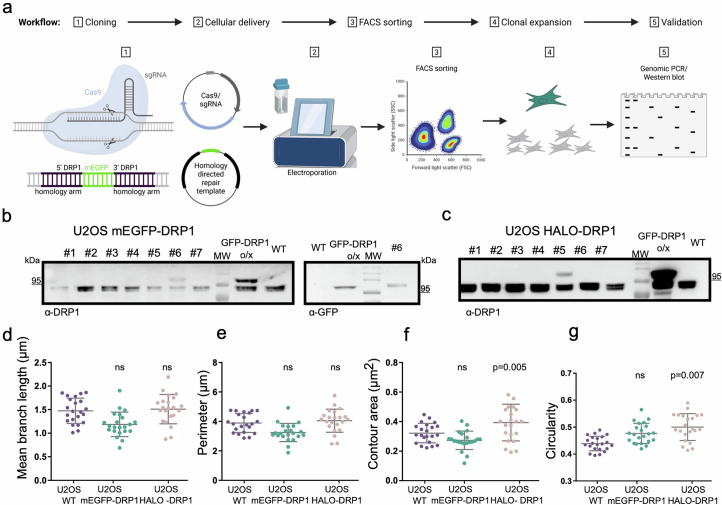
Generation and validation of U2OS mEGFP- and HALO-DRP1 cell lines. **a**) Graphical representation of the workflow for the generation and validation of a U2OS cell line with endogenous tagging of DRP1 using mEGFP via a CRISPR/Cas9-based approach. A synthetic guide RNA (sgRNA) targeting the endogenous DRP1 locus was cloned into a vector together with the Cas9 coding sequence. Homology arms (800 bp each), corresponding to regions upstream and downstream of the DRP1 start codon, were fused to the mEGFP coding sequence and inserted into a homology-directed repair (HDR) template vector. Both the Cas9/sgRNA construct and the HDR template were delivered to U2OS cells by electroporation. Cells were analyzed by flow cytometry, and individual GFP-positive cells were sorted, with gating performed to exclude dead cells using the DAPI channel. Single-cell clones were expanded and validated by PCR genotyping of genomic DNA, immunoblotting, and fluorescence imaging. Image created with Biorender under CC BY 4.0 (Jenner, A. (2026) https://BioRender.com/uqowvit). **b**) Western blot analysis of lysates from U2OS mEGFP-DRP1 single-cell clones (as indicated by the clone number), wild-type U2OS cells (WT), and cells transiently overexpressing GFP-DRP1, serving as negative and positive controls, respectively. The clone-screening wb was performed once; validation of mEGFP-DRP1 expression was confirmed in 3 independent experiments. **c**) Western blot analysis of lysates from U2OS HALO-DRP1 cell clones (as indicated by the clone number), wild-type U2OS cells (WT), and cells transiently overexpressing GFP-DRP1, serving as negative and positive controls, respectively.The clone-screening wb was performed once; validation of HALO-DRP1 expression was confirmed in 3 independent experiments. **d-g**) Quantification of mitochondrial morphological parameters, mean branch length (**d**), perimeter (**e**), area (**f**), and circularity (**g**), performed using MitoSkel1 based on the mean of n = 3 independent experiments. Data are presented as mean ± SEM, with each point representing an individual cell. Normality of the data distribution was assessed using the Shapiro–Wilk test, equality of variances was evaluated using an F-test, followed by statistical comparison to the WT group using an unpaired two-tailed Student’s t-test. Source numerical data and unprocessed blots are available in source data. Illustrations in **a** created in BioRender; Jenner, A. https://www.biorender.com/uqowvit (2026). Source data

**Extended Data Fig. 2:**
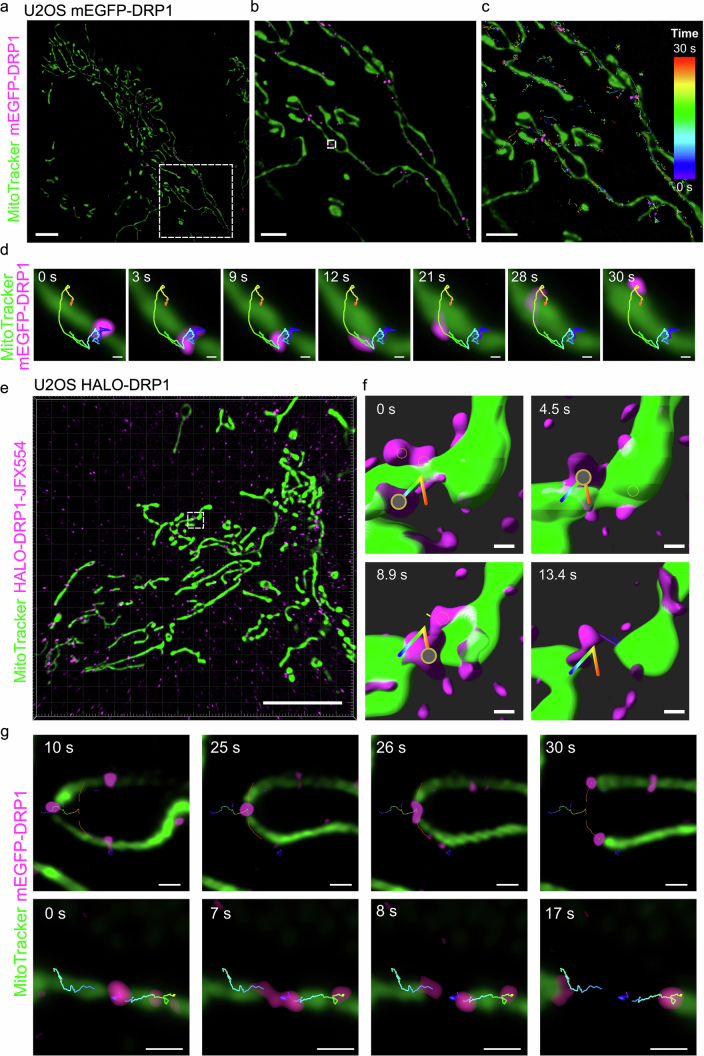
mEGFP- and HALO-DRP1 oligomers scan the surface of mitochondria with spiral-like trajectories. **a**) Snapshot from a 2D SIM movie of a U2OS mEGFP-DRP1 cell showing mitochondria in green labelled with MitoTracker Deep Red (100 nM) and mEGFP-DRP1 in magenta. Scale bar: 5 µm.Representative of 3 independent experiments. **b**) Zoomed-in view of the boxed region in panel (**a**). Scale bar: 2µm.Representative of 3 independent experiments. **c**) Zoomed-in view of the boxed region in panel (**a**), showing mEGFP-DRP1 particle trajectories color-coded by time. Scale bar: 2 µm. **d**) Time-lapse SIM images of a mEGFP-DRP1 particle trajectory on the mitochondrial surface, color-coded by time. Mitochondria in green labelled with MitoTracker Deep Red and mEGFP-DRP1 in magenta. Scale bar: 100 nm. Representative of 3 independent experiments **e**) Snapshot from a 3D SIM movie of a U2OS HALO-DRP1 cell showing mitochondria in green labelled with MitoTracker Deep Red (100 nM) and HALO-DRP1 in magenta labelled with JFX554 (300 nM). Representative of 3 independent experiments. **f**) 3D rendering of a zoomed-in view of the boxed region in panel (**e**). Scale bar, 500 nm. Representative of 3 independent experiments. **g**) Representative snapshots from 2D SIM movies of a U2OS mEGFP-DRP1 cell undergoing mitochondrial fission. mGFP-DRP1 is shown in magenta and the mitochondrial network labelled with MitoTracker Deep Red in shown in green. Scale bar, 500 nm. Representative of 3 independent experiments. Source data

To investigate how DRP1 dynamics contributes to mitochondrial fission, we generated U2OS cell lines expressing DRP1 endogenously tagged with mEGFP (mEGFP–DRP1) or with HALO-tag (HALO–DRP1) using CRISPR–Cas9 technology (Extended Data Fig. 1a). While we cannot discard the presence of untagged DRP1 in the cells on the basis of western blot analysis (Extended Data Fig. 1b), we confirmed the functionality of mEGFP–DRP1 in the U2OS cell line by quantitatively comparing mitochondrial morphology with that of wild-type (WT) cells (Extended Data Fig. 1d–g). We then visualized the dynamic subcellular distribution of mEGFP–DRP1 under steady-state conditions with high temporal and spatial resolution using live-cell two-dimensional (2D) and three-dimensional (3D) structured illumination microscopy (SIM). The signal of endogenous mEGFP–DRP1 on mitochondria appeared as individual particles, corresponding to oligomers of unknown stoichiometry, but bright enough to be detected by SIM. To our surprise, mEGFP–DRP1 was not simply recruited to defined fission sites at mitochondria, as reported in previous studies using confocal microscopy under overexpression conditions^7,20^, but exhibited high motility on the mitochondrial surface (Fig. 1, Extended Data Fig. 2a–c and Supplementary Videos 1–3). Strikingly, diffusing mEGFP–DRP1 oligomers often exhibited in spiral-like trajectories resulting from the movement along the mitochondrial axis accompanied by rotation around that axis (Fig. 1d and Extended Data Fig. 2d). HALO–DRP1 particle tracking in 3D live cell imaging of U2OS HALO–DRP1 confirmed the motion of HALO–DRP1 around the mitochondrial tubule (Fig. 1e, Extended Data Fig. 2e,f and Supplementary Videos 4 and 5).

Under our physiological conditions, the majority of mEGFP–DRP1 oligomers diffusing on mitochondria were not involved in fission events during the acquisition time. Yet, we found that some of them accumulated at discrete, immobile puncta that ultimately evolved into mitochondrial fission sites (Fig. 1f, Extended Data Fig. 2g and Supplementary Video 3). Direct visualization of events in which scanning DRP1 oligomers stalled at to-be fission sites revealed how they halted their diffusive motion and condensed through rotational movements around the constricted site, which eventually underwent fission. We quantified the mitochondrial diameter at the site of mEGFP–DRP1 stalling relative to the dynamics of DRP1 arrival and accumulation at these sites (Fig. 1g). Remarkably, the mitochondrial diameter was narrower at the sites where mEGFP–DRP1 accumulated, compared with the normal diameter of 300–500 nm for unconstructed mitochondria^40^ indicating preconstriction at to-be fission sites. The mitochondrial diameter continued to decrease as mEGFP–DRP1 accumulated until fission took place.

**Extended Data Fig. 3:**
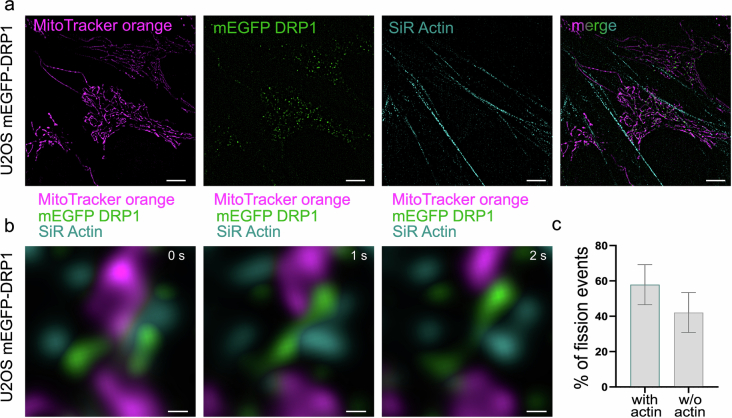
Actin localization at mitochondrial fission events. **a**) Snapshot from a 2D SIM movie of a U2OS mEGFP-DRP1 cell showing mitochondria in magenta labelled with MitoTracker orange (100 nM), mEGFP-DRP1 in green and actin in cyan, labelled with SiR actin(100 nM). Scale bar: 5 µm. Representative of 3 independent experiments. **b**) Zoomed-in view of a fission event showing mitochondria in magenta labelled with MitoTracker orange and mEGFP-DRP1 in green and actin in cyan, labelled with SiR actin. Scale bar: 300 nm. **c**) Quantification of the percentage of mitochondrial fission events occurring with or without SiR actin signal localized at the constriction site. Data are presented as mean ± SEM from 3 independent experiments. Source data

Because fission sites have been reported to be marked by endoplasmic reticulum (ER)-actin-based constriction of mitochondria^41^, we also imaged DRP1 stall sites in conjunction with actin filaments (Extended Data Fig. 3 and Supplementary Video 6); the reduced temporal resolution precluded quantitative analysis of DRP1 scanning dynamics under these conditions. Nevertheless, actin filaments were detectable at sites of DRP1 accumulation (Extended Data Fig. 3), consistent with previous studies^40^.

We also found that the two mEGFP–DRP1 particles resulting from the splitting of the complex at the tips of the two daughter mitochondria had similar brightness and were each approximately half as bright as the original particle. These results reveal that DRP1 oligomers redistribute roughly equally into two after fission (Fig. 1h and Supplementary Video 3). Interestingly, mEGFP–DRP1 particles at the mitochondrial tips generally presented continuous rotating movements (Fig. 1i and Supplementary Video 7), suggesting that they might be kinetically trapped at these sites of increased membrane curvature.

### Stable DRP1 oligomers move on the MOM through free diffusion

**Fig. 2:**
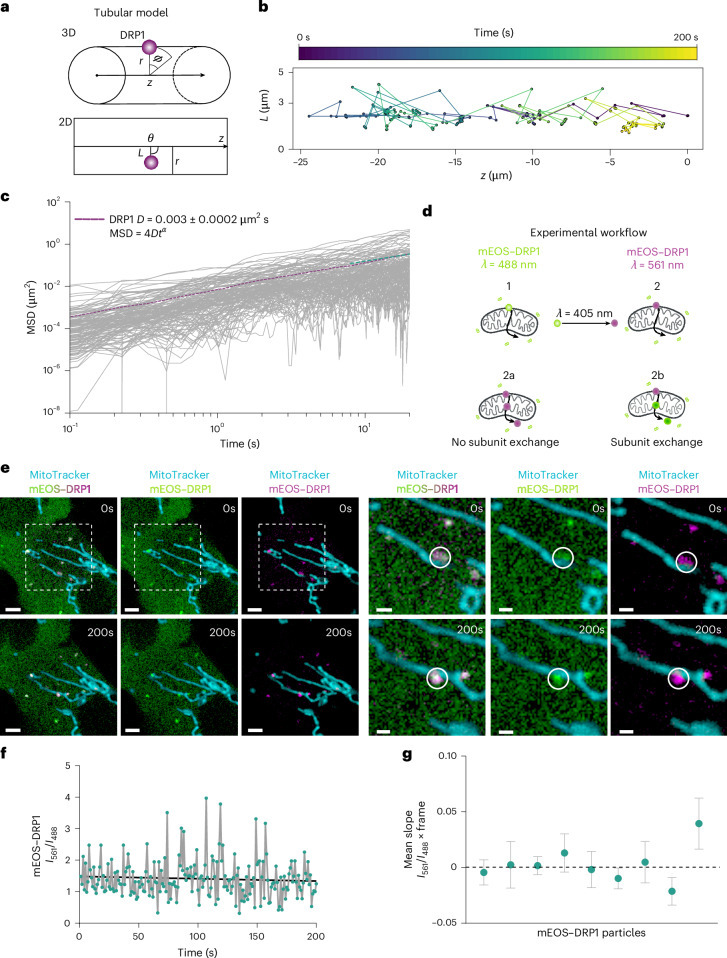
DRP1 mito-scanning motion proceeds via free diffusion of stable oligomers. **a**, Schematic representation of the ‘tubular model’, depicting mitochondria as a cylindrical tube with a DRP1 particle represented as a green sphere on its surface. The 3D model includes the *z* axis, radius (*r*) and angle (*ϕ*), measured from the top view and ranging from −90° to 90°. The lower part of the model shows a 2D projection of a mitochondrion, in which the distance (*L*) of the COM of DRP1 perpendicular to the centre axis (*z*) of the mitochondrion, the radius (*r*) and the perpendicular angle (*θ*) are used to calculate the angle (*ϕ*) in cylindrical coordinates. **b**, Representative trajectory of the COM of mEGFP–DRP1 molecules diffusing along the mitochondrial surface, with different timepoints indicated by the colour scale. **c**, Log–log plot of the MSD of the COM of 202 mEGFP–DRP1 particles with trajectories longer than 15 s. The MSD shows linear behaviour in the long-time regime (averaged in the dashed magenta line) and can be fitted with a free-diffusion model (dashed green line), yielding a diffusion coefficient of 0.003 ± 0.0002 µm^2^ s^−1^. **d**, Schematic illustration of the experimental workflow for DRP1 photoconversion experiments. DRP1 is tagged with the photoconvertible protein mEOS, which fluoresces green (*λ* = 488 nm excitation) before photoconversion (1). Upon photoconverting illumination at *λ* = 405 nm, assembled mEOS–DRP1 oligomers on the MOM emit in red (magenta, *λ* = 561 nm excitation), while the cytosolic pool remains largely unconverted (2). If DRP1 oligomers are stable, the magenta signal would persist over time, indicating no detectable subunit exchange (2a). By contrast, if subunit exchange occurs, unconverted cytosolic mEOS–DRP1 would gradually incorporate into the oligomer, leading to increased green fluorescence and a decreasing magenta/green intensity ratio (2b). **e**, Left: confocal time-lapse imaging of HeLa DRP1-KO cells transiently expressing mEOS–DRP1 (green) and mitochondria stained with MitoTracker Deep Red (cyan) immediately after photoconversion (*t* = 0 s) and 200 s after conversion. Scale bars, 3 µm. Right: zoom-in of a representative mEOS–DRP1 particle (white circle), as indicated by the dashed rectangle. Scale bars, 1 µm. **f**, Quantification of red (561 nm)/green (488 nm) intensity (*I*) ratio over time for individual DRP1 particles. Linear regression fit shown as black line. **g**, Distribution of slope values calculated from the 561/488 intensity ratio per frame, based on *n* = 9 independent measurements from different cells. Only particles tracked for more than 20 s were included. Each dot represents one cell; data are presented as mean ± s.d. Source data

Given the unexpected high motility detected for mEGFP–DRP1 particles on the mitochondrial surface (Fig. 1 and Supplementary Video 1), we further investigated the underlying mode of motion. For this, we developed an analytical approach that accounts for the movement of mitochondria within the cell during video acquisition. In this method, mEGFP–DRP1 particles are represented as spheres identified by their centre of mass (COM) and tracked over time, while each individual mitochondrion is modelled as a tube (Fig. 2a). The 2D SIM microscopy images provide a projection of each individual mitochondrial tube of length *z* and radius *r*, from which the distance of the mEGFP–DRP1 particles to the centre and their perpendicular angle *θ* can be measured to calculate the angle *ϕ* in cylindrical coordinates. Our tubular model also allows the quantification of the distance (*L*) between the centre of the mitochondrial tube and the COM of each mEGFP–DRP1 particle along its entire trajectory. A representative trajectory is shown in Fig. 2b. Notably, this method can be generally applied to study the motion of other protein complexes at mitochondria as well as at other tubular organelles, such as the ER.

**Extended Data Fig. 4:**
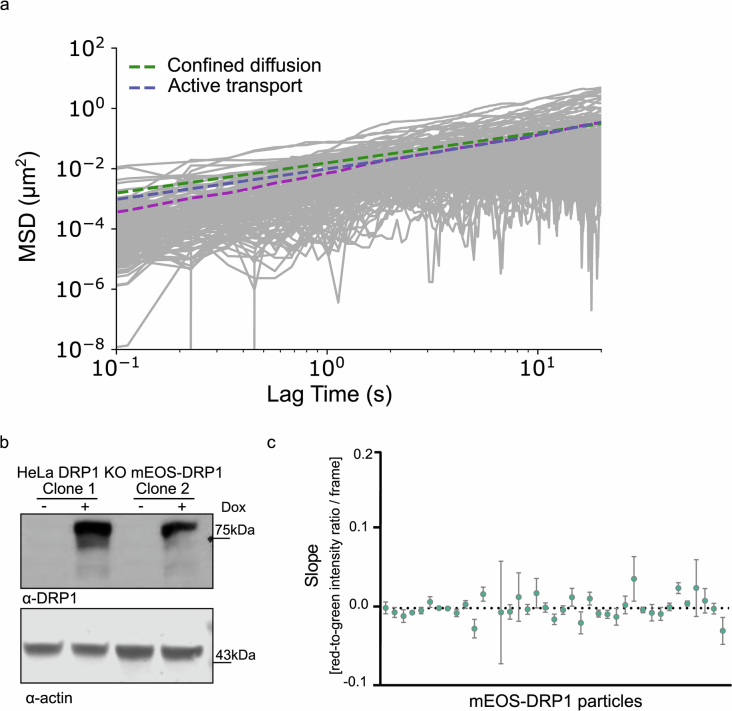
DRP1 mito-scanning motion proceeds via free diffusion of stable oligomers. **a**) Log–log plot of the time-averaged mean-squared displacement (MSD) of the centre of mass of 202 mitochondrial mEGFP-DRP1 particles with trajectories longer than 15 s. Individual trajectories are shown in grey. The pink dashed line is DRP1 MSD, active transport (violet), and confined diffusion (green). The MSD displays a linear behaviour in the long-time regime, consistent with free diffusion. The characteristic ballistic behavior in active transport isn’t recovered and no confinement is observed when using these models. **b**) Western blot analysis of lysates from HeLa DRP1 KO cell clones (as indicated by the clone number), stably transfected with doxycycline-inducible mEOS-DRP1. Western blot was probed against DRP1 (top panel) and against actin (loading control, bottom panel). Representative of 3 independent experiments. **c**) Distribution of slope values (561/488 intensity ratio per frame) from linear fit of fluorescence intensity ratio over time analyzed from n = 39 independent measurements across n = 22 different cells, with particles tracked for more than 20 s. Data are presented as individual measurements (individual data points) ± SEM (whiskers). Dashed line at x = 0 to indicate a slope of 0 representing no exchange in the fluorescence intensity ratio over time. Source numerical data and unprocessed blots are available in source data. Source data

We selected all trajectories longer than 15 s from our SIM videos and calculated their mean square displacement (MSD) as a function of time. For the over 200 mEGFP–DRP1 trajectories analysed, the MSD presented a linear increase in the long-term regime, as shown by the average MSD curve (Fig. 2c, magenta), which fit best to a model of free diffusion (Fig. 2c, dashed green line). From this analysis, we determined a diffusion coefficient of *D* = 0.003 ± 0.0002 µm^2^ s^−1^, significantly slower than that obtained for the diffusion of the TOM complex^42,43^ but in line with the larger size and more complex supramolecular structure of DRP1 oligomers. Fitting the average MSD curve to a confined diffusion model yielded confinement lengths far exceeding any physically relevant spatial scale of the system, effectively reducing the model to the free diffusion limit. Similarly, inclusion of a drift term in an active transport model did not produce a detectable quadratic scaling regime, and the fitted velocities were insufficient to generate ballistic behaviour within the experimental time window. In both cases, the models collapse to normal diffusion, indicating that a simple diffusive description provides the minimal and physically consistent interpretation of the data (Extended Data Fig. 4a). Our findings suggest that DRP1 particles constitutively scan and explore the mitochondrial surface through diffusion, before accumulating and constricting at a specific site to initiate a fission event.

We next attempted to clarify whether DRP1 displaces via a treadmill-like mechanism or whether it moves as stable oligomeric assemblies. To this aim, we transiently overexpressed DRP1 tagged with the photoconvertible fluorescent protein mEOS in HeLa DRP1 KO. Upon ultraviolet illumination, mEOS undergoes a structural rearrangement that shifts its fluorescence emission from green to red^44^. Individual mEOS–DRP1 oligomers at the MOM were initially localized in the green channel and then photoconverted to red by ultraviolet irradiation, leaving cytosolic mEOS–DRP1 largely unconverted. If mitochondrial mEOS–DRP1 particles displaced at the expense of the cytosolic mEOS–DRP1 pool, the incorporation of green cytosolic mEOS–DRP1 subunits would cause a decrease in the red/green fluorescence intensity ratio over time (Fig. 2d). By contrast, if the mEOS–DRP1 oligomers at the mitochondrial surface were stable in composition during the acquisition time, the red/green fluorescence intensity ratio of single mEOS–DRP1 particles would be constant over time (Fig. 2d). We included in our analysis only individual mEOS–DRP1 assemblies that could be tracked on the MOM for more than 50 consecutive frames (a time of approximately 200 s) after photoconversion (Fig. 2e–g and Supplementary Video 8).

We found that the fluorescence intensity of individual, photoconverted mEOS–DRP1 particles in the red channel remained predominantly constant throughout the imaging period, with minimal increase in green fluorescence (Fig. 2f). We also quantified the slope of the red/green fluorescence intensity ratio over time for individual mEOS–DRP1 particles and plotted the mean ± s.d. for each individual case (Fig. 2g). For all particles analysed, the distribution of slopes was centred around zero with no consistent trend (Fig. 2g), indicating no significant change in the red/green fluorescence ratio. Similar results were obtained using a stable inducible cell line expressing mEOS-tagged DRP1 under doxycycline regulation at levels similar to physiological conditions (Extended Data Fig. 4b,c). These data suggest that DRP1 forms stable oligomers on the MOM with minimal subunit exchange with the cytosolic DRP1 pool at the spatiotemporal resolution of our assay, which, given their speed on the MOM, rules out a treadmilling model of displacement.

Notably, mGFP tagging of DRP1 has been reported to enhance its oligomerization^45^. Although this potential limitation should be considered, this effect does not seem to impact the ability of mEGF–DRP1 oligomers to ‘scan’ the mitochondrial surface, given that the ‘scanning’ motion does not require exchange. Yet, because smaller oligomers not visualized in our study may exchange, we cannot fully exclude a contribution of assembly/disassembly to DRP1 dynamics.

### Molecular dynamics simulations of DRP1 diffusion on mitochondria

**Fig. 3:**
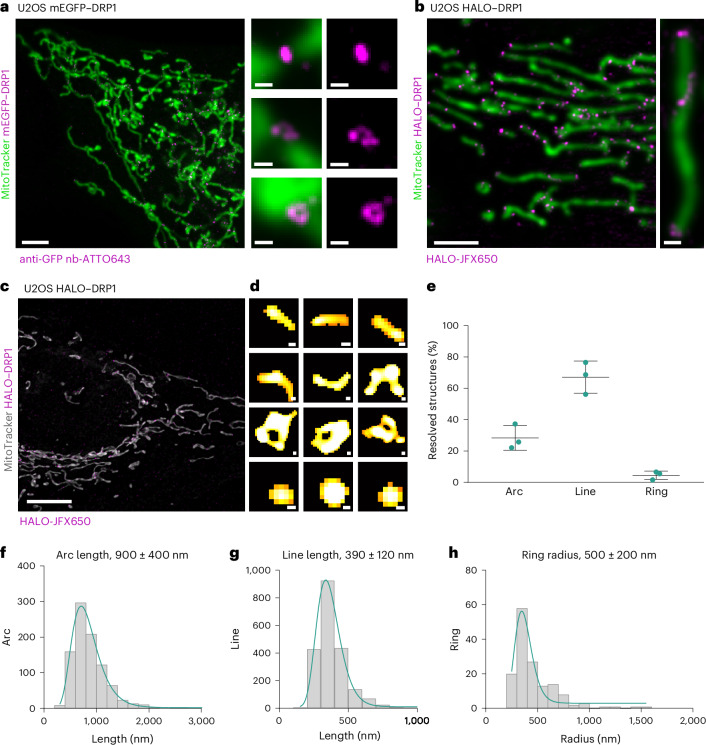
Super-resolution analysis of DRP1 assemblies. **a**, Snapshot from a STED microscopy image of a U2OS mEGFP–DRP1 cell showing mitochondria labelled with MitoTracker orange (green) and mEGFP–DRP1 labelled with anti-GFP nanobodies coupled with ATTO643 (magenta). The zoomed-in views (right) highlight the super-resolved structure of non-random DRP1 macromolecular assemblies, appearing as lines, arcs and rings. Scale bars, 3 µm (overview) and 500 nm (zoom). **b**, Representative STED image of a U2OS HALO–DRP1 cell, with mitochondria labelled with MitoTracker orange (green) and HALO–DRP1 stained with Janelia Fluor 650 (magenta). A zoomed-in view reveals super-resolved DRP1 assemblies. Scale bars, 3 µm (overview) and 50 nm (zoom). Representative of three independent experiments. **c**, Snapshot from a SIM microscopy image of a U2OS HALO–DRP1 cell showing mitochondria labelled with MitoTracker orange (grey) and HALO–DRP1 with JFX650 (magenta). Scale bar, 3 µm. **d**, Representative images of DRP1 macromolecular assemblies identified using automated structures analysis program (ASAP) analysis, classified as lines, arcs, rings and unresolved dots^56^. Scale bars, 50 nm. Representative of three independent experiments. **e**, Quantification of the relative distribution of resolved, non-random DRP1 structures as lines, arcs and rings, expressed as a percentage of the total number of assemblies analysed. Each dot represents one independent replicate (*n* = 3). Data are presented as mean ± s.e.m. (total *N* = 3,128 structures). **f**, Histogram showing the size distribution of DRP1 arc lengths, with bin size and total number of assemblies indicated in the graph and a log-normal fit (*R*^2^ = 0.9957). **g**, Histogram showing the size distribution of DRP1 line lengths, with bin size and total number of assemblies indicated and a Gaussian fit (*R*^2^ = 0.9867). **h**, Histogram showing the size distribution of DRP1 ring radii, with bin size and total number of assemblies indicated and a log-normal fit (*R*^2^ = 0.9283). Source data

We reasoned that the spiral-like trajectories observed for DRP1 dynamics on mitochondria might result from the coupling between the spatial arrangement of DRP1 oligomers and the tubular geometry of mitochondria. To explore this possibility, we performed a shape analysis of endogenous DRP1 tagged with mEGFP and HALO-tag visualized by stimulated emission depletion (STED) microscopy and in SIM images (Fig. 3a–c,h). While the large majority of particles corresponded to dots, among the resolved structures we detected a strong enrichment of line and arc assemblies compared with closed rings (Fig. 3d,e). Notably, given the spatial resolution of SIM, we could not resolve DRP1 structures associated with constricted fission sites. It is thus likely that the few rings observed correspond to arcs or spirals observed from a tilt angle. We found that the length of the arc-shaped assemblies was centred at around 900 nm, approximately twice that of linear structures, which would be insufficient to span the perimeter of unconstricted mitochondria of around 1,500 nm (ref. ^5^).

**Fig. 4:**
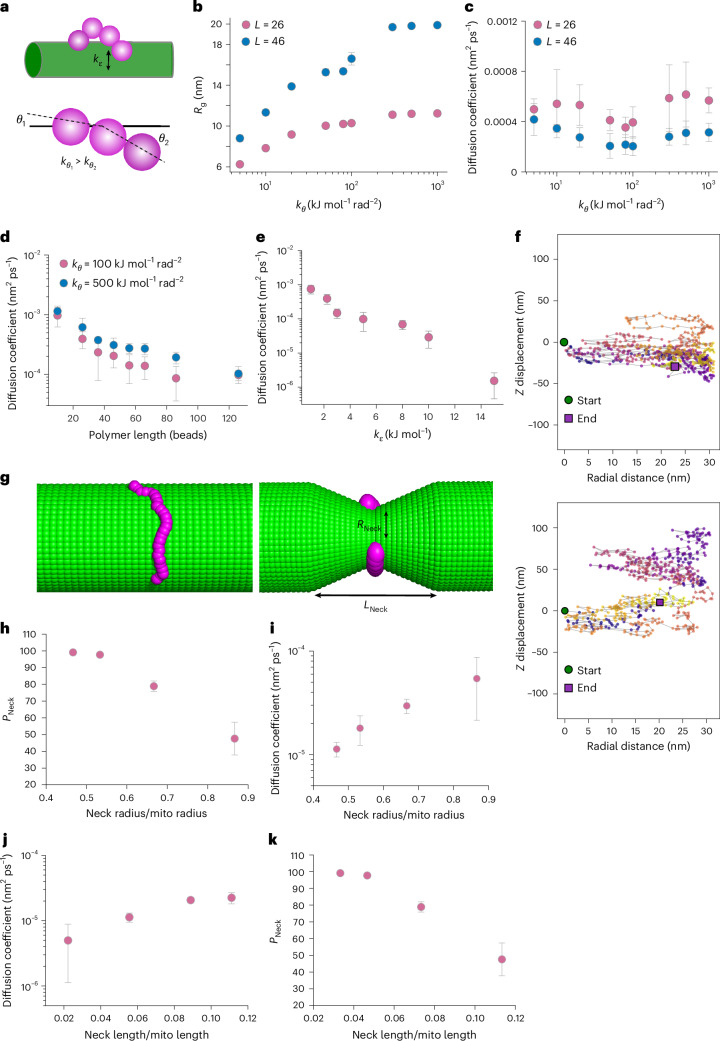
Coarse-grained simulation model and polymer diffusion dynamics. **a**, Schematic of the coarse-grained (CG) simulation model. A single polymer chain, representing DRP1, is constrained to diffuse along the surface of a rigid cylindrical scaffold that mimics the MOM. Polymer flexibility is controlled by an angular potential with tunable force constant ${k}_{\theta }$ (kJ mol⁻^1^ rad^−2^), which modulates the radius of gyration and conformational ensemble. Polymer–surface interactions are described by a binding potential with force constant ${k}_{\varepsilon }$ (kJ mol⁻^1^). **b**, ${R}_{{\rm{g}}}$ as a function of ${k}_{\theta }$ for the indicated polymer lengths (*L*). As stiffness increases, ${R}_{{\rm{g}}}$ approaches a plateau consistent with a fully rigid configuration. Data were obtained from five independent simulation runs (*n* = 5) and are presented as mean ± s.d. **c**, Diffusion coefficient of the polymer as a function of ${k}_{\theta }$ for the same polymer lengths, revealing non-monotonic behaviour. For the longer polymer ($L=26$ beads), two distinct peaks in diffusivity are observed at ${k}_{\theta }=10$ and 500 kJ mol⁻^1^ rad^−2^. Data were obtained from five independent simulation runs (*n* = 5) and are presented as mean ± s.d. **d**, Diffusion coefficient as a function of polymer length for the indicated values of ${k}_{\theta }$. Polymers with higher rigidity generally exhibit greater diffusivity than more flexible polymers, indicating that ${R}_{{\rm{g}}}$ alone does not fully dictate surface mobility. At a given stiffness, polymers achieve maximal diffusivity by aligning with the cylinder axis, whereas both excessive flexibility and excessive rigidity reduce scanning efficiency. Data were obtained from five independent simulation runs (*n* = 5). and are presented as mean ± s.d. **e**, Diffusion coefficient as a function of binding strength ${k}_{\varepsilon }$. Stronger polymer–surface interactions markedly diminish diffusivity. Diffusion coefficients in **c**–**e** were obtained by averaging values from *n* = 5 independent simulation replicates. Data were obtained from five independent simulation runs (*n* = 5) and are presented as mean ± s.d. **f**, Representative trajectories of the polymer COM for an $L=26$ system with ${k}_{\varepsilon }$ = 2.2 kJ mol⁻^1^ at two stiffness regimes: ${k}_{\theta }$ = 20 kJ mol⁻^1^ rad⁻^2^ (top) and ${k}_{\theta }$ = 500 kJ mol⁻^1 ^rad^−2^ (bottom). **g**, Snapshots of polymer on tube and tube containing a neck of a given radius (*R*) and length (*L*). **h**,**i**, Probability of residence at the cylinder neck (**h**) and diffusion coefficient (**i**) as a function of neck radius. As overall diffusivity increases, the polymer shows reduced propensity to localize at the neck region. Data were obtained from ten independent simulation runs (*n* = 10). and are presented as mean ± s.d. **j**,**k**, Diffusion coefficient (**j**) and probability of residence at the cylinder neck (**k**) as a function of neck length. Probability values were derived from *n* = 10 independent simulations and represent the averaged polymer residence time within ±1.3 nm of the neck centre, thereby incorporating local positional fluctuations. Data were obtained from ten independent simulation runs (*n* = 10) and are presented as mean ± s.d. mito, mitochondria. Source data

Next, we developed a coarse-grained molecular dynamics model in which the mitochondrion is represented as a rigid cylindrical scaffold aligned with the *z* axis of the simulation box and a single polymer chain, representing a DRP1 oligomer, is constrained to diffuse along the cylindrical surface (Fig. 4a). Polymer stiffness is controlled by an angular potential acting on the polymer backbone, with the angular force constant (*k*_*θ*_) serving as a key parameter to probe the coupling between oligomer conformation and surface diffusion.

The angular force constant (*k*_*θ*_, kJ mol⁻^1^ rad^−2^) determines the polymer’s equilibrium radius of gyration (*R*_g_). As *k*_*θ*_ increases, *R*_g_ increases and eventually saturates at the polymer’s contour length (Fig. 4b). Because translational diffusion is inversely related to polymer size, we systematically varied *k*_*θ*_ for oligomers of two different lengths and quantified the resulting diffusion coefficient (*D*). This analysis revealed a non-monotonic dependence of *D* on *k*_*θ*_, with a pronounced minimum at intermediate stiffness values (Fig. 4c and Supplementary Video 9). At low *k*_*θ*_, the polymer adopts a compact conformation and diffuses rapidly. Increasing *k*_*θ*_ initially slows diffusion as the polymer extends, but further stiffening leads to an increase in *D*. This behaviour contrasts with simple 3D diffusion, where *D* typically scales as *R*_g_^−1^, and highlights the importance of anisotropic confinement on a curved surface.

To separate the effects of polymer length and stiffness, we simulated oligomers with identical bead numbers but different *k*_*θ*_ values. As expected, *D* decreased with increasing polymer length (Fig. 4d). Importantly, for a given length, stiffer polymers diffused faster than more flexible, compact ones. This counterintuitive result indicates that polymer extension along the cylindrical surface reduces effective frictional drag, thereby enhancing mobility.

We next examined the role of specific DRP1–mitochondrion interactions by varying the polymer–cylinder interaction strength (*k*_*ε*_). For a 26-bead oligomer with fixed stiffness (*k*_*θ*_ = 100 kJ mol⁻^1^ rad^−2^), increasing *k*_*ε*_ caused a monotonic decrease in *D* (Fig. 4e). This result is in line with our experimental observations and indicates that receptor-mediated tethering is a dominant regulator of DRP1 surface diffusion.

Representative COM trajectories illustrate the constrained motion of the polymer along the cylindrical surface (Fig. 4f). Together, these simulations reproduce the elongated and curved conformations of DRP1 observed experimentally and establish a mechanistic framework in which surface-bound diffusion is governed by polymer stiffness, length, interaction strength and membrane geometry. This provides a physical basis for mechanical feedback in the DRP1–mitochondria interaction and supports the proposed mito-scanner model.

Given the frequent occurrence of mitochondrial constriction during fission, we extended the model to include cylinders with a tunable central neck, mimicking mechanically deformed mitochondrial membranes. The constricted geometry is defined by two parameters: the neck radius (*R*_Neck_) and the neck length (*L*_Neck_), which control the depth and spatial extent of the constriction, respectively (Fig. 4g). We first quantified the effect of *R*_Neck_ on oligomer dynamics. As the neck radius decreased, the diffusion coefficient dropped markedly and the probability of polymer localization within the neck region (*P*_Neck_) increased (Fig. 4h,i and Supplementary Video 10). Notably, even modest constrictions (*R*_Neck_/*R*_mito_ ≈ 0.9) were sufficient to induce strong oligomer trapping, consistent with experimental observations that mitochondrial structural cues influence DRP1 dynamics. We then investigated the role of constriction length by varying *L*_Neck_ at a fixed *R*_Neck_/*R*_mito_ ≈ 0.9. Increasing *L*_Neck_ led to higher diffusion coefficients and reduced trapping probabilities, allowing the polymer to escape the constricted region more readily (Fig. 4j,k). These results indicate that both the depth and the longitudinal extent of a geometric deformation regulate the stability of oligomer condensation.

While these simulations provide a simplistic model to test whether diffusion of an oligomer on a cylindrical surface reproduces basic aspects of DRP1 motility in cells, they do not capture all elements of the physiological context. Yet, they strengthen a model in which local mitochondrial geometry acts as a direct physical regulator of DRP1 diffusion, with neck-like constrictions acting as structural cues that mark to-be fission sites for DRP1 stalling and maturation into fission complexes. They also provide a quantitative biophysical framework for understanding how membrane deformations bias DRP1 oligomers towards localized trapping, thereby increasing the likelihood of downstream mitochondrial fission events.

### Altering DRP1 dynamics impairs mitochondrial fission efficiency

**Extended Data Fig. 5:**
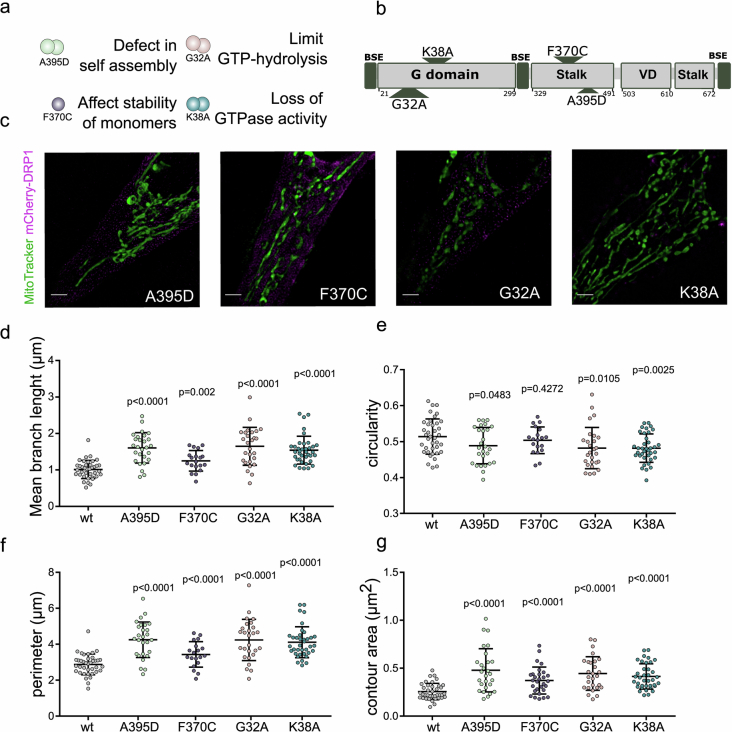
Effects of mutations on DRP1self-assembly and mitochondrial morphology. **a**) Schematic representation of the effects of different DRP1 mutants on DRP1 self-assembly, GTP hydrolysis and receptor interaction. The A395D mutation impairs self-assembly properties of DRP12. The F370C mutation affects DRP1 monomer stability and tetramer formation, rendering DRP1 unable to remodel mitochondrial membranes at constriction sites3. The G32A mutation impairs GTP hydrolysis, resulting in defects in mitochondrial localization and higher-order assembly, limiting hydrolysis and preventing membrane constriction4. The K38A mutation results in the loss of GTPase activity5. **b**) Schematic representation of DRP1 mutants, with variants shown according to the domain distribution of DRP1 (amino acid positions correspond to DRP1 isoform 3). **c**) Representative SIM image of DRP1 knockout (KO) HeLa cells transfected with wild-type (WT) mCherry-DRP1 or the indicated DRP1 mutant plasmids. Scale bar: 3 µm. Representative of 3 independent experiments. **d-g**) Quantification of mitochondrial morphological parameters, mean branch length (**d**), circularity (**e**), perimeter (**f**), and contour area (**g**), performed using MitoSkel based on the mean of n = 3 independent experiments. Data are presented as mean ± SEM. Each point represents an individual cell. Normality of data distribution was assessed using the Shapiro–Wilk test. For normally distributed data, statistical comparison to the WT group was performed using Student’s t-test. Exact p-values for all comparisons are indicated in the figure. Statistical significance was defined as p < 0.05. Source numerical data are available in source data. Source data

To test whether core mechanistic and enzymatic activities of DRP1 affect its dynamics on mitochondria, we analysed disease-related mutants defective in self-assembly, GTP binding/hydrolysis or higher-order oligomerization (Extended Data Fig. 5a,b). Specifically, we analysed disease-associated mutations located in distinct DRP1 domains: A395D, located in the stalk, impairs DRP1 self-assembly^46^; F370C, also in the stalk, disrupts tetramer formation and membrane remodelling; mutant G32A in the GTPase domain is defective in GTP hydrolysis and higher-order assembly^47^; and K38A, also located in the GTPase domain, leads to a complete loss of GTPase activity^29^. However, these mutants failed to localize to mitochondria, which precluded any scanning motion on the organelle (Extended Data Fig. 5c). Expression of WT mEGFP–DRP1 in DRP1-KO cells efficiently rescued the fragmented mitochondrial phenotype and restored a highly interconnected mitochondrial network (Extended Data Fig. 5d–g). By contrast, cells expressing the mEGFP–DRP1 mutants showed extensive mitochondrial elongation and absence of mEGFP–DRP1 foci at mitochondria (Extended Data Fig. 5c). These DRP1 variants often formed cytosolic aggregates or exhibited diffuse cytosolic localization. Quantitative analysis of mitochondrial branch length and area confirmed significant differences compared with DRP1 WT, with all mutants inducing a hyperfused mitochondrial network.

**Fig. 5:**
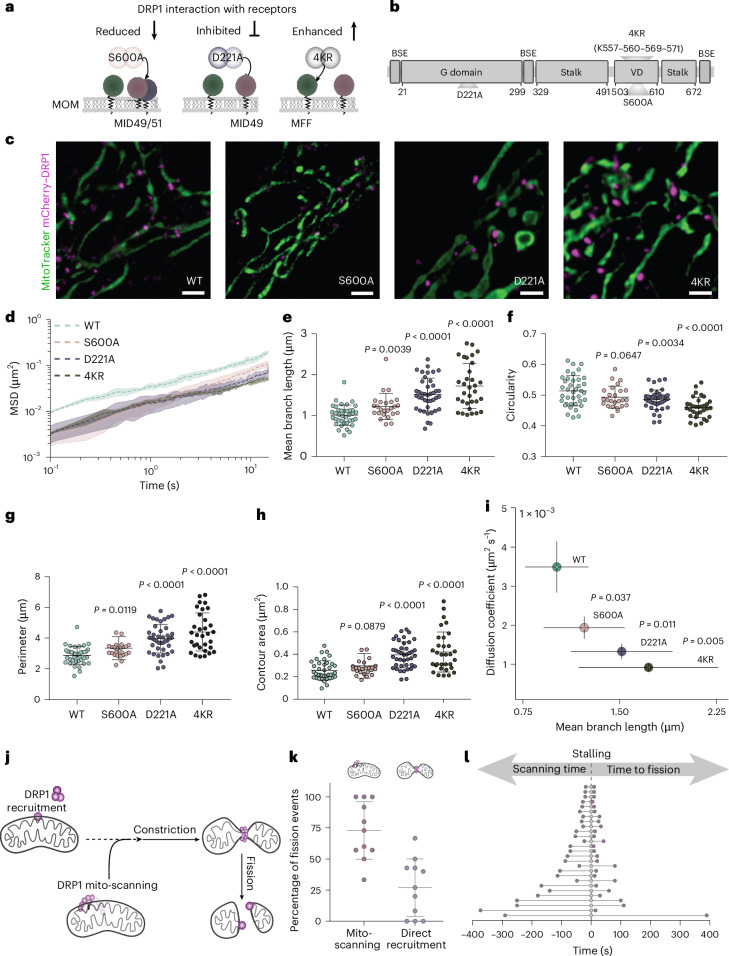
Altering DRP1 dynamics impairs mitochondrial fission efficiency. **a**, Schematic summary of the reported effects of DRP1 mutants on interactions with DRP1 receptors. The S600A mutation reduces interaction with MiD49/51^48^, D221A disrupts interaction with MID49^24^ and 4KR enhances interaction with MFF^49^. **b**, Schematic representation of DRP1 mutants, with variants displayed according to the domain organization of DRP1 (amino acid positions correspond to DRP1 isoform 3). **c**, Representative SIM images of DRP1 KO HeLa cells overexpressing WT mCherry–DRP1 or the indicated mutants (magenta). Mitochondria labelled with MitoTracker (green). Scale bars, 3 µm. **d**, Log–log plot of the MSD averaged from three independent experiments of DRP1 particles over time, derived from *n* = 202 trajectories longer than 15 s. MSD mean values are shown as a dashed line, and the shaded area represents the s.d. Statistical significance was assessed using a two-sided *t*-test. **e**–**h**, Quantification of mitochondrial morphological parameters—mean branch length (**e**), circularity (**f**), perimeter (**g**) and area (**h**)—in cells expressing mCherry–DRP1 mutant versions S600A, D221A and 4KR compared with WT. Data are shown as individual measurements (data points) and mean (line) ± s.e.m. (whiskers) from *n* = 3 independent experiments. Normality was assessed before statistical testing. Normally distributed datasets were analysed using an unpaired two-tailed Student’s *t*-test, whereas non-normally distributed datasets were analysed using a two-tailed Mann–Whitney test. Mutants versus WT: **e**, S600A, *P* = 0.0039; D221A, *P* < 0.0001; 4KR, *P* < 0.0001; **f**, S600A, *P* = 0.0647; D221A, *P* = 0.0034; 4KR, *P* < 0.0001; **g**, S600A, *P* = 0.0119; D221A, *P* < 0.0001; 4KR, *P* < 0.0001; **h**, S600A, *P* = 0.0879; D221A, *P* < 0.0001; 4KR, *P* < 0.0001. Statistical significance was defined as *P* < 0.05. **i**, Diffusion coefficient of DRP1 mutants as a function of mitochondrial mean branch length. s.d. is shown in the horizontal direction for mean branch length and in the vertical direction for diffusion coefficient. Statistical significance was assessed using unpaired two-tailed *t*-tests comparing each mutant with WT (S600A, *P* = 0.037; D221A, *P* = 0.011; 4KR, *P* = 0.005). **j**, Schematic representation of direct DRP1 recruitment versus scanning DRP1 recruitment to mitochondrial fission sites. **k**, DRP1 particles classified as mitochondrial scanning or direct recruitment to fission sites. Each point represents an individual particle, with data pooled from three independent experiments performed on different days (scanning, *n* = 49; recruitment, *n* = 16; total *N* = 65 particles). Recruitment excludes particles that were immobile at fission sites at the beginning of acquisition. **l**, Time from mitochondrial recruitment to scanning, stalling and fission. Analysis was restricted to fully observed mitochondrial scanning events starting from recruitment and culminating in fission. Time was aligned to the onset of DRP1 immobilization (stalling), defined as *t* = 0. Negative values represent the scanning phase preceding stalling, and positive values indicate the time from stalling to fission. Each line represents a single event (*n* = 27 events from multiple independent measurements). Source data

We then decided to explore milder mutations that do not directly interfere with DRP1 activity. A key step in DRP1-mediated mitochondrial fission is its recruitment to the MOM by adaptor proteins^28^. We thus used point mutation versions of DRP1 that affect its interaction with the receptors and measured their diffusion over time (Fig. 5a–i). The S600A mutation in the VD of DRP1 reduces its interaction with MID49 and MID51^48^, while the D221A mutation in the GTPase domain disrupts the interaction with MID49^24^ (Fig. 5a,b). We also tested the non-SUMOylatable DRP1 K557/560/569/571R (4KR) mutant in the VD, which has been reported to bind ~2.5-fold more effectively to MFF compared with DRP1 WT^49^ (Fig. 5a,b). We transfected HeLa DRP1-KO cells with mCherry-tagged WT DRP1 or its mutant versions and confirmed that the S600A, D221A and 4KR mutants showed a subcellular distribution similar to DRP1 WT, forming particles located on the mitochondrial surface (Fig. 5c).

We then performed single-particle tracking (SPT) analysis for mCherry–DRP1 WT and mutants and obtained a linear increase of the MSD with time, indicating a diffusive behaviour of mCherry–DRP1 particles (Fig. 5d). Our analysis also showed that all mutant versions of DRP1 had lower diffusion than DRP1 WT. Although we cannot discard other, unknown effects of these mutations, our findings suggest that reducing the interaction with MID49, or increasing the interaction with MFF, reduces the scanning dynamics of DRP1 on the MOM.

To investigate whether altering DRP1 scanning diffusion has a functional effect on DRP1-mediated mitochondrial fission, we performed a quantitative analysis of the mitochondrial network morphology using the Mitoskel software^50^. We quantified geometric descriptors of mitochondria, such as mean branch length, circularity, perimeter and contour area from SIM images (Fig. 5e–h). All three mutations associated with decreased DRP1 motility resulted in changes in mitochondrial morphology compared with the WT protein, with the D221A and 4KR mutants showing the most pronounced changes. Remarkably, we found DRP1 motility correlated negatively with mitochondrial branch length, supporting that the presence of elongated mitochondria in these mutants results from impaired DRP1 dynamics (Fig. 5i).

To further strengthen the causal relationship between DRP1 mito-scanning motion and mitochondrial fission, we quantified the number of fission events in which DRP1 arrived to the fission site via scanning versus via direct recruitment (Fig. 5j). As shown in Fig. 5k, DRP1 reached fission sites through scanning in the majority of the cases, underscoring DRP1 mito-scanning as a major mechanism for DRP1 enrichment at fission sites and, thus, for mitochondrial fission. For those trajectories in which we could capture the full process of recruitment of an individual mEGFP–DRP1 particle followed by scanning, stalling and fission, we recorded scanning paths of heterogeneous duration reaching up to hundreds of seconds before stalling. After stalling at prefission sites, the time to fission was also relatively heterogeneous for the individual particles analysed (Fig. 5l).

Collectively, these data indicate that mitochondrial scanning is a common mechanism for DRP1 to reach fission sites and that regulation of DRP1 scanning dynamics through interaction with its mitochondrial adaptors modulates the correct efficiency of fission events and thus mitochondrial morphology.

### DRP1 diffuses on the MOM in dynamic co-assemblies with MID49

**Fig. 6:**
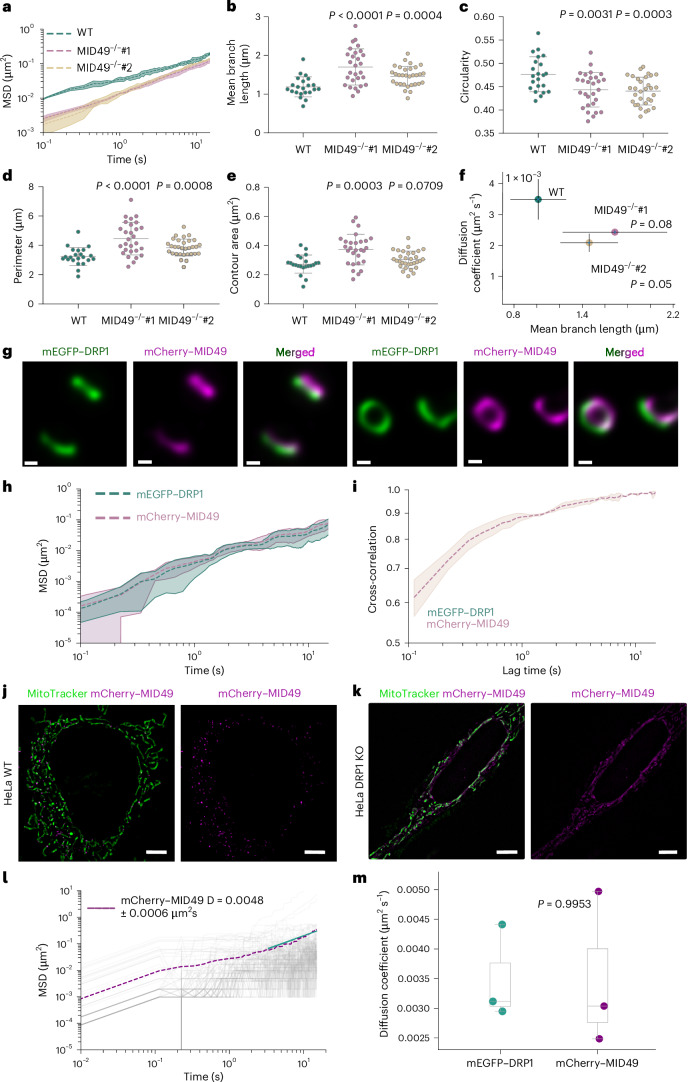
DRP1 diffuses on the MOM in dynamic co-assemblies with MID49. **a**, Log–log plot displaying the MSD of mEGFP–DRP1 particles in U2OS mEGFP–DRP1 MID49 KO clones #1 and #2. Data were collected from 200 trajectories, each lasting longer than 15 s. Mean MSD is shown as a dashed line, and the shaded area represents the s.d. **b**–**e**, Quantification of mitochondrial morphological parameters: mean branch length (**b**), circularity (**c**), perimeter (**g**) and area (**e**) with MitoSkel^50^ based on the mean of *n* = 3 independent experiments. Data are shown as individual measurements (data points) and mean (line) ± s.e.m. (whiskers) from *n* = 3 independent experiments. Normality was assessed before statistical testing. Normally distributed datasets were analysed using an unpaired two-tailed Student’s *t*-test, whereas non-normally distributed datasets were analysed using a two-tailed Mann–Whitney test. Exact *P* values are indicated in the figure. MID49 KO #1 versus WT and MID49 KO #2 versus WT: **b**, *P* < 0.0001 and *P* = 0.0004; **c**, *P* = 0.0031 and *P* = 0.0003; **d**, *P* < 0.0001 and *P* = 0.0008; **e**, *P* = 0.0003 and *P* = 0.0709. Statistical significance was defined as *P* < 0.05. **f**, Diffusion coefficient as a function of mitochondrial mean branch length for WT and U2OS mEGFP–DRP1 MID49 KO clones #1 and #2. Each point represents the mean of *n* = 3 independent experiments. Vertical error bars indicate s.e.m. of the diffusion coefficient, and horizontal error bars indicate s.e.m. of the mitochondrial mean branch length. Statistical differences between groups were assessed using an unpaired two-tailed *t*-test. MID49 KO #1 versus WT, *P* = 0.08; MID49 KO #2 versus WT, *P* = 0.05. **g**, Representative live cell SIM images showing co-diffusing macromolecular assemblies formed by mEGFP–DRP1 (green) and mCherry–MiD49 (magenta). Scale bars, 200 nm. Representative of three independent experiments. **h**, MSD analysis of mEGFP–DRP1 and mCherry–MID49 trajectories. Data from 57 particles pooled from multiple cells and independent measurements are shown. Mean MSD is shown as dashed lines and s.d. by shaded areas. **i**, Displacement cross-correlation analysis of mEGFP–DRP1 and mCherry–MID49 trajectories. The mean is shown as a dashed line and the s.d. as shaded area. **j**, Snapshot from a 2D SIM video of a HeLa WT cell showing mitochondria labelled with MitoTracker Green (green) and mCherry–MID49 appearing in discrete foci (magenta). Scale bars, 4 µm. **k**, Snapshot from a 2D SIM video of a HeLa DRP1 KO cell showing mitochondria labelled with MitoTracker Green (green) and mCherry–MiD49 homogeneously distributed on mitochondria (magenta). Scale bars, 5 µm. **l**, Log–log plot of the MSD of the COM of mCherry–MID49 particles with trajectories longer than 15 s. Mean MSD is shown in magenta, with a fit to a free-diffusion model (green) yielding a diffusion coefficient of 0.0048 ± 0.0002 µm^2^ s^−1^. **m**, Diffusion coefficients of mEGFP–DRP1 and mCherry–MID49 particles derived from MSD analysis. Each dot represents one independent experiment. Centre line, median; box limits, 25th and 75th percentiles; whiskers, minimum to maximum. Statistical significance was assessed using an unpaired two-tailed Student’s *t*-test: *P* = 0.9953. Statistical significance was assessed using an unpaired two-tailed *t*-test: *P* = 0.9953. Source data

To validate whether the altered DRP1 dynamics observed in these mutants were caused by impaired DRP1/MID49/51 interaction rather than other defects in DRP1 activity, we generated MID49-KO cell lines from the U2OS cells endogenously expressing mEGFP–DRP1, clones (#1 and #2), and performed SPT of mEGFP–DRP1 with live cell SIM. Consistent with our findings for the mEGFP–DRP1 D221A mutant, which selectively disrupts interaction with MID49^24^, loss of MID49 resulted in a clear reduction of mEGFP–DRP1 diffusion (Fig. 6a,f) that was accompanied by significant mitochondrial elongation as quantified by MitoSkel (Fig. 6b–e). These results indicate that, also in MID49-KO cells, as in the experiments with the DRP1 D221A mutant, reduced DRP1 motility compromised the surface scanning mechanism with consequences for adequate mitochondrial division.

To further validate the effect of the interaction with MID49 on DRP1 motility, we turned to a second system based on HeLa cells double KO in DRP1 and MID49, in which fluorescently labelled DRP1 and MID49 were transiently expressed. Using this system, we identified macromolecular co-assemblies between mEGFP–DRP1 and mCherry–MID49 in living cells (Fig. 6g), which had been previously reported only in vitro^24^. Remarkably, these co-assemblies formed linear and ring-shaped structures that co-diffused with overlapping intensity profiles, demonstrating direct interaction between both protein assemblies (Supplementary Video 11). SPT analysis of mEGFP–DRP1 and mCherry–MID49 also revealed coordinated motion, shown by matched trajectories and overlapping MSD profiles (Fig. 6h). We also evaluated the dynamic coupling between mEGFP–DRP1 and mCherry–MID49 by calculating the displacement cross-correlation function. As shown in Fig. 6i, the cross-correlation approaches a value of 1 with increasing lag times, confirming strong dynamic coupling, and therefore interaction, between mEGFP–DRP1 and mCherry–MID49.

This raised the question whether MiD49 might be underlying the mito-scanner motion observed for DRP1. To test this possibility, we performed live-cell SIM imaging of mCherry–MID49 in HeLa WT and DRP1-KO cells. We found that DRP1 influenced the mitochondrial distribution of mCherry–MID49, which appears as discrete foci in WT cells, but homogeneously distributed along the MOM in the absence of DRP1 (Fig. 6j,k). We then performed SPT on mCherry–MID49 discrete particles in WT cells, where we found that these structures exhibit diffusion properties comparable to those of DRP1 assemblies (Fig. 6l,m).

**Extended Data Fig. 6:**
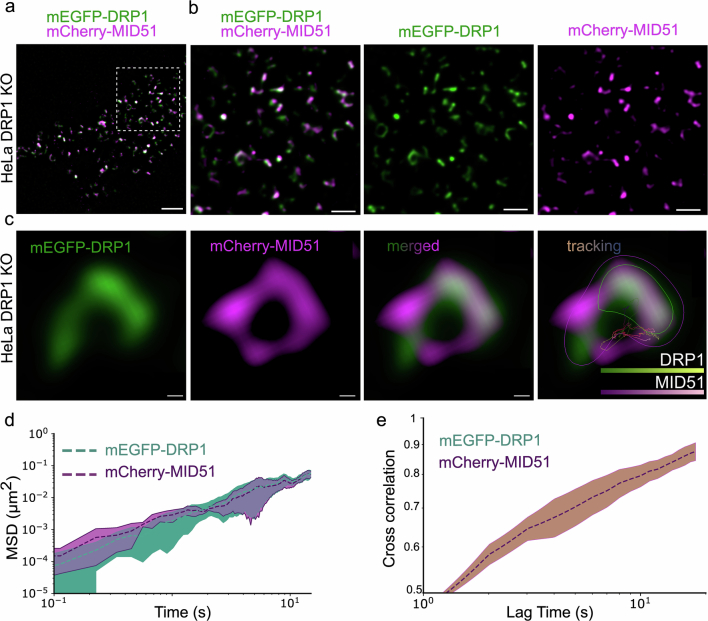
DRP1 forms dynamic co-assemblies with MID51. **a**) Snapshot from a 2D SIM time-lapse movie of a HeLa DRP1 knockout (KO) cell overexpressing mEGFP–DRP1 (green) and mCherry–MID51 (magenta). Scale bar, 2 µm. Representative of 3 independent experiments. **b**) Zoom in region from panel (**a**) showing mEGFP-DRP1 in green, and mCherry-MID51 in magenta. Scale bar, 1 µm. **c**) Representative 2D SIM images showing macromolecular assemblies formed by mEGFP–DRP1 (green) and mCherry–MID51 (magenta). Individual channels, merged image, and exemplary particle tracks are shown. Representative of 3 independent experiments. **d**) Mean squared displacement (MSD) analysis of mEGFP-DRP1 and mCherry-MID51 trajectories. Data from 60 particles pooled from multiple cells and 3 independent measurements are shown. The mean is indicated by dashed lines and the SD by shaded areas. **e**) Displacement cross-correlation analysis of DRP1 and MID51 trajectories. The mean is shown as a magenta dashed line and the SD as shaded areas. Data are derived from 3 independent experiments. Source numerical data are available in source data. Source data

We also performed two-colour live-cell SIM imaging experiments with mCherry–MID51 and mEGFP–DRP1 in HeLa DRP1-KO cells. We found that mEGFP–DRP1/mCherry–MID51 also co-diffused together as complexes, similar to MID49, with comparable MSDs (Extended Data Fig. 6). However, the correlation analysis between the GFP–DRP1 and mCherry–MID51 signal was lower than for mCherry–MID49, indicating that a fraction of mCherry–MI51 is not interacting with mEGFP–DRP1 under our experimental conditions.

**Fig. 7:**
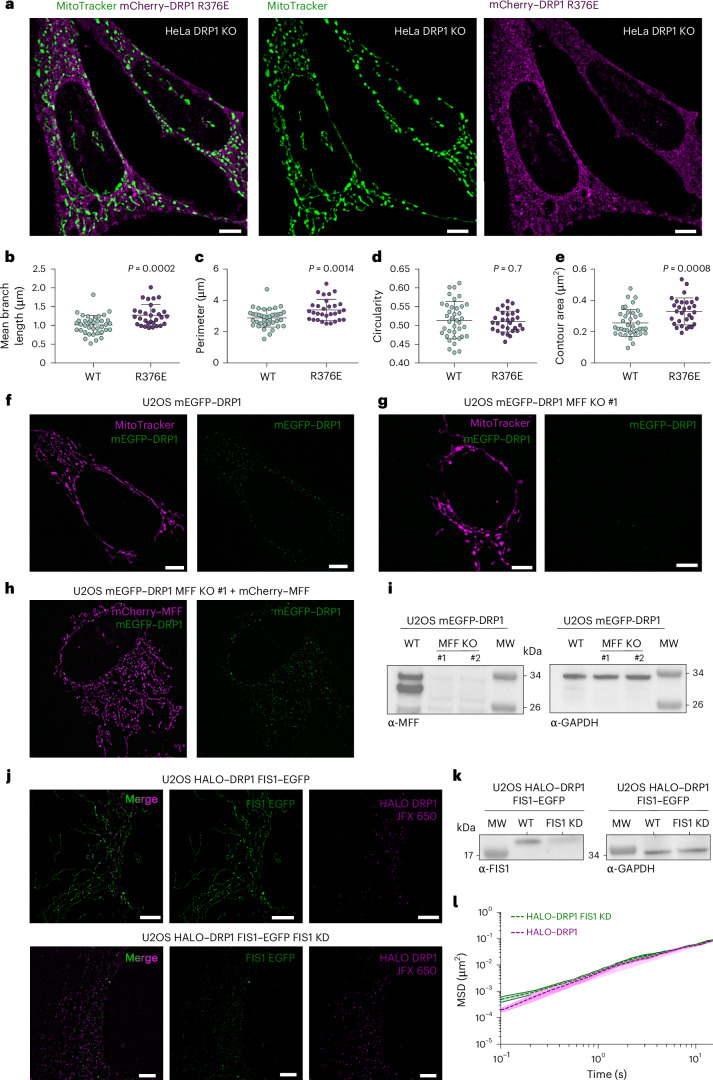
MFF is required for DRP1 recruitment to mitochondria. **a**, Representative SIM images of DRP1 KO HeLa cells overexpressing mCherry–DRP1 R376E (magenta). Mitochondria were labelled with MitoTracker (green). Scale bars, 5 µm. Representative of three independent experiments. **b**–**e**, Quantification of mitochondrial morphological parameters: mean branch length (**b**), perimeter (**c**), circularity (**d**) and contour area (**e**), analysed using MitoSkel^50^. Data represent the mean of *n* = 3 independent experiments. Each point corresponds to an individual cell. Data are shown as mean ± s.e.m. Normality was assessed using the Shapiro–Wilk test. For normally distributed datasets, comparisons with WT were performed using an unpaired two-tailed Student’s *t*-test. Exact *P* values are indicated in the figure. Mutant versus WT: **b**, *P* = 0.0002; **c**, *P* = 0.0014; **d**, *P* = 0.7; **e**, *P* = 0.0008. Statistical significance was defined as *P* < 0.05. **f**, Representative SIM images of U2OS mEGFP–DRP1 cells (green). Mitochondria were labelled with MitoTracker Deep Red (magenta). Scale bars, 5 µm. Representative of three independent experiments. **g**, Representative SIM images of U2OS mEGFP–DRP1 MFF KO clone 1. Scale bars, 5 µm. Representative of three independent experiments. **h**, Representative SIM images of U2OS mEGFP–DRP1 MFF KO clone 1 transiently overexpressing mCherry–MFF. Representative of three independent experiments. **i**, Western blot analysis of lysates from U2OS mEGFP–DRP1 cells and U2OS mEGFP–DRP1 MFF KO clones 1 and 2. Western blot was probed against MFF and against GAPDH (loading control). Representative of three independent experiments. MW, molecular weight; kDa, kilodaltons. **j**, Representative SIM images of U2OS HALO–DRP1 (magenta) FIS1–EGFP (green) cells (top) and FIS1 knockdown (40 nM siRNA, 72 h) (bottom). Representative of three independent experiments. Scale bars, 4 µm (top); 5 µm (bottom). **k**, Western blot analysis of lysates from U2OS HALO–DRP1 FIS1–EGFP cells ± FIS1 knockdown (40 nM siRNA, 72 h). Blots were probed for FIS1 and GAPDH (loading control). Representative of three independent experiments. **l**, Log–log plot of the MSD of HALO–DRP1 particles in U2OS HALO–DRP1 FIS1–EGFP cells ± FIS1 knockdown (40 nM siRNA, 72 h). The mean is shown as dashed lines, and the shaded areas represent the s.d. Source data

**Extended Data Fig. 7:**
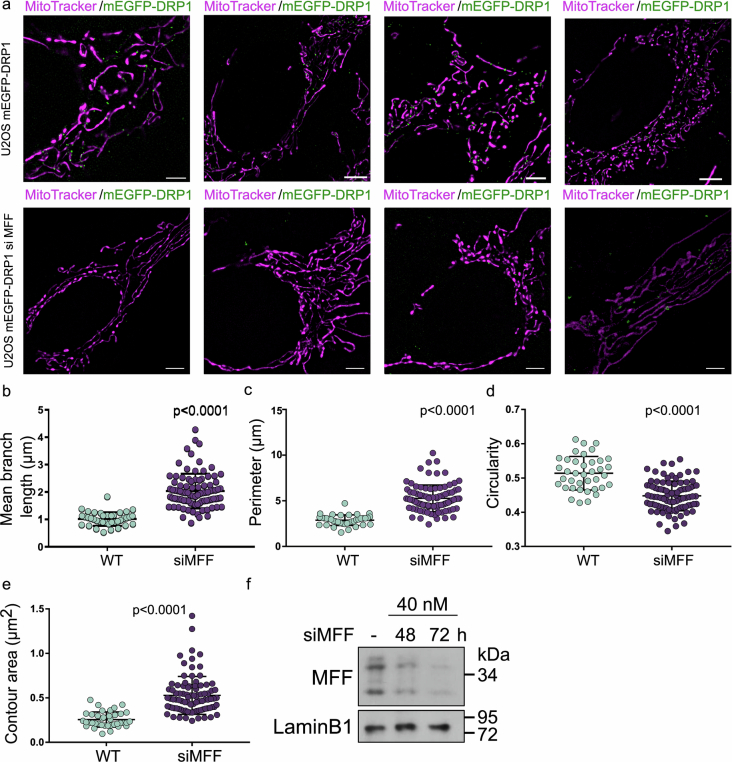
MFF is required for DRP1 foci formation at the mitochondrial outer membrane. **a**) 2D SIM snapshots from U2OS mEGFP-DRP1 cells treated with siMFF (40 nM, 72 h). The upper panel shows control cells; the lower panel shows siMFF-treated cells. Mitochondria are shown in magenta, labelled with MitoTracker Deep Red (100 nM), and mEGFP-DRP1 in green. In WT cells, DRP1 appears as discrete mitochondrial foci, whereas in siMFF-treated cells DRP1 is absent from mitochondria or forms cytosolic aggregates. Scale bar, 5 µm. Representative of 3 independent experiments. **b-e**) Quantification of mitochondrial morphological parameters, mean branch length (**b**), perimeter (**c**), circularity (**d**), and contour area (**e**), performed using MitoSkel1 based on the mean of n = 3 independent experiments. Data are presented as mean ± SEM. Each point represents an individual cell. Normality of data distribution was assessed using the Shapiro–Wilk test. For normally distributed data, statistical comparison to the WT group was performed using Student’s t-test. Exact p-values for all comparisons are indicated in the figure. Statistical significance was defined as p < 0.05. **f**) Western blot analysis of lysates from U2OS mEGFP-DRP1 cells with and without siMFF 40nM at 48 and 72h. Western blot was probed against MFF (top panel) and against LaminB1 (loading control, bottom panel). Representative of 3 independent experiments. Source numerical data and unprocessed blots are available in source data. Source data

To evaluate the potential contribution of other adaptors to mito-scanning by DRP1, we investigated the effect of MFF and FIS1. We analysed the dynamic behaviour of DRP1 under conditions of no interaction with MFF with live cell SIM imaging. For this, we used a mutant of mCherry–DRP1 that is deficient in MFF binding, R376E^26,51^, transiently expressed in HeLa DRP1-KO cells (Fig. 7a–e). In contrast to WT DRP1, the DRP1 R376E mutant showed a more homogeneous cytosolic distribution, indicating impaired formation of discrete DRP1 foci. Cells expressing DRP1 R376E displayed a more elongated mitochondrial network, supporting the role of the DRP1–MFF interaction in promoting DRP1 foci formation, accumulation at mitochondria and efficient mitochondrial fission. As an alternative approach, we also analysed the dynamics of DRP1 endogenously tagged with mEGFP in U2OS cells in which we knocked down and knocked out MFF (Fig. 7f–i and Extended Data Fig. 7). In all cases, the mitochondrial localization of DRP1 was lost, and with that its mito-scanner activity, which was accompanied by an elongation of the mitochondrial network. Reconstitution of MFF in KO cells rescued mEGFP–DRP1 mitochondrial localization and foci formation (Fig. 7h). Live-cell SIM imaging of transiently expressed mCherry–MFF in U2OS GFP–DRP1 cells knocked out in MFF also showed that mCherry–MFF mostly presents a homogeneous distribution, suggesting a lack of co-diffusion with DRP1 in discrete complexes (Fig. 7h). These results are in line with a role of MFF in the recruitment of DRP1 to mitochondria^38^, upstream of the mito-scanner activity. However, they do not rule out that continued interactions with MFF molecules—for example, through on–off dynamics—may contribute to association with the mitochondrial surface even during the ‘scanning’ process, thereby playing a role in it.

In the case of FIS1, we found that FIS1–EGFP presented a more homogeneous distribution, without forming discrete foci along the mitochondrial surface. Also, FIS1–EGFP and HALO–DRP1 did not co-localize together in U2OS cells expressing both proteins endogenously tagged (Fig. 7j). Furthermore, in this system, depleting FIS1 had no significant effect on the diffusion coefficient of HALO–DRP1 (Fig. 7k,l). These results indicate that FIS1 does not contribute to DRP1 mito-scanning activity.

Together, our results reveal distinct effects of DRP1 adaptors on its mito-scanner activity. While MFF is required for DRP1 recruitment to mitochondria, and with that, for mito-scanning, our data suggest that it does not form discrete mito-scanning complexes. By contrast, the diffusive behaviour of DRP1 is tightly bound to its interaction with the mitochondrial adaptor protein MID49/51 as part of mito-scanning complexes containing both proteins, thereby also revealing that it is a regulated process.

## Discussion

Here, we report on the dynamics of DRP1 at mitochondria during steady-state conditions in healthy cells. Contrary to textbook models in which DRP1 is directly recruited at discrete sites on mitochondria to mediate organelle fission, we show that it exhibits high motility on the MOM, freely diffusing in a mito-scanner fashion with a functional role for mitochondrial homeostasis.

The free diffusion of DRP1 on the MOM results in spiral-like trajectories along the mitochondrial network. This is probably a consequence of the coupling between the tubular structure of the MOM and the conformational organization of DRP1 oligomers into curved assemblies with a defined persistence length, as supported by our dynamic simulations. While the copy number of diffusive DRP1 oligomers remains unknown at this point, our images suggest that their length is insufficient to cover the MOM perimeter of a non-constricted mitochondrion. At preconstricted to-be fission sites, we measured an average diameter of 180 ± 30 nm (Fig. 1g), with a perimeter that could then be covered by DRP1 oligomers, allowing head-to-tail and/or lateral interactions between DRP1 monomers within a spiral- or ring-shaped oligomer.

We visualize how DRP1 oligomers halt their diffusive motion and condense through rotational movements around narrower constricted prefission sites followed by mitochondrial fission. After this, fragments of DRP1 oligomers of roughly similar size stay associated with the two tips of the newly generated daughter mitochondria, where they continue to rotate as if kinetically trapped at the highly curved mitochondrial ends. These observations strongly suggest that the curvature or radius of the organelle strongly influences DRP1 motility along the mitochondrial network, leading to accumulation at sites of higher curvature such as constricted prefission sites and mitochondrial tips, in line with the molecular dynamics simulations. Based on this, it is tempting to speculate that initial constriction mediated by the ER and the actin cytoskeleton at mitochondrial fission sites could serve as structural cues to halt diffusive DRP1 particles, so that they can condense to further constrict and divide the organelle.

Remarkably, we find that the diffusive motion of DRP1 is a regulated process that depends on the strength of its interaction with its mitochondrial adaptors. Perturbing these interactions through point mutations results in reduced DRP1 motility and in elongated mitochondria, which underscores the functional relevance of the mito-scanner motion of DRP1 for adequate mitochondrial network organization. This is especially relevant for MID49/51. Cells lacking MID49 presented reduced DRP1 diffusion and elongated mitochondria, revealing a role for this protein in the regulation of DRP1 motility. In line with this, we find that DRP1 and MID49/51 co-diffuse as a complex during the mito-scanner motion of DRP1. Although these experiments were performed under overexpression conditions, the differences in how MID51 and MID49 interact with DRP1 raise questions about potential three-way interactions among DRP1, MID49 and MID51 and how they are regulated, which warrant further investigation.

We propose a model for the mito-scanner function of DRP1 based on the distinct effects of DRP1 adaptors on DRP1 dynamics, in which the interplay among the receptors ensures precise spatial and temporal control over mitochondrial division. After recruiting DRP1 oligomers from the cytosol^38^, we speculate that MFF might contribute to the interaction strength of DRP1 with the mitochondrial surface through on/off interactions between homogeneously distributed MFF molecules and diffusing, discrete DRP1/MID49/51 complexes. Considering the linear filaments reported for DRP1/MID49 assemblies^24^, one could envision that the association of MID49/51 with DRP1 stabilizes larger oligomers by impairing GTPase activity^24^, contributing to the oligomer stiffness required for efficient mito-scanning, in line with our molecular dynamics simulations. Here, the non-overlapping properties of MID49 and MID51 might fine-tune the stiffness of the complexes with DRP1. Further work will be necessary to elucidate the molecular determinants that regulate the interaction of DRP1 with the different receptors, including during the transition from scanning to constricting behaviour.

Our findings may have wider implications for elongated oligomers diffusing on the surface of cellular organelles. For example, DRP1 is also involved in peroxisome fission. Given that peroxisomes have a different geometry, one would expect that the diffusion of DRP1 on the organelle surface may give rise to different trajectories. Alternatively, peroxisome elongation might influence DRP1 motility on the organelle and with that the fission process. In the broader sense, it is tempting to speculate that other GTPases of the dynamin family might exhibit ‘scanning’-like diffusion as part of their function in membrane fission and fusion. The supercoiling of lipid nanotubes by dynamin was interpreted as a rotatory movement of the helix turns^52^. A recent study on Mitofusins reports their enrichment at fusion and fission sites^53^, which would be compatible with a similar dynamic behaviour. Based on our model, one would expect that the geometry of the membrane would strongly influence the resulting motility of the dynamin oligomer diffusing on it. Visualization of the oligomers dynamics at high spatiotemporal resolution and endogenous expression will help elucidate this question. By contrast, a treadmilling rotating motion around the septum marking the site for membrane fission was reported for the tubulin homologue FtsZ in bacterial cell division^54,55^, indicating that alternative modes of motion can also be used for promoting membrane fission reactions.

In summary, our findings support a model in which DRP1 oligomers bound to MID49/51 dynamically scan the MOM to locate preconstricted to-be fission sites. Upon stalling at these sites, DRP1 reorganizes to constrict the mitochondrion, culminating in membrane scission. The observed diffusive behaviour of DRP1 arises from the coupling between the oligomer and mitochondrial surface relative geometries and suggests that the stochastic search for fission sites limits the fission process. Consequently, reduced DRP1 motility compromises the efficiency of the surface scanning mechanism required for timely fission site identification, ultimately leading to reduced mitochondrial division and the formation of an elongated mitochondrial network. Our results underscore mitochondrial division not as an isolated event but as a regulated process involving steps of browsing, sensing and execution, with implications for therapeutic intervention in mitochondrial disorders.

## Methods

All experiments were performed in accordance with institutional guidelines. The use of established cell lines did not require ethical approval. Data collection and analysis were not performed blind to the conditions of the experiments. No data points were excluded from the analyses.

### Cell culture

U2OS WT, U2OS mEGFP–DRP1, U2OS HALO–DRP1, U2OS mEGFP–DRP1 MID49-KO, HeLa DRP1-KO and HeLa DRP1-KO mEOS–DRP1 cells were cultured at 37 °C and 5% CO_2_ in Dulbecco’s modified Eagle medium supplemented with 10% fetal bovine serum and 1% penicillin–streptomycin (Invitrogen). All cell lines used in this study were checked against the International Cell Line Authentication Committee database of commonly misidentified cell lines. HeLa cells, which are listed by the International Cell Line Authentication Committee database as being associated with cross-contamination issues, were used because the DRP1-KO cell line employed in this study is derived from a HeLa background. The use of this model is well established in the field. Cell line identity was based on prior validation by the providing laboratories. The U2OS mEGFP–DRP1 and HALO–DRP1 cell lines generated in this study were validated by locus-specific polymerase chain reaction (PCR), sequencing and fluorescence imaging. All cell lines were regularly tested for mycoplasma contamination and were confirmed to be negative.

### Generation of U2OS mEGFP–DRP1 and HALO–DRP1 knock-in cell line using CRISPR–Cas9

CRISPR–Cas9-mediated knock-in of the fluorescent protein mEGFP and HALO-tag into the DRP1 locus was achieved using a combinatorial approach. WT U2OS cells were electroporated with the SpCas9-2A-GFP vector (Addgene #48138^57^, which encodes the SpCas9 enzyme and a single guide RNA (sgRNA) targeting DRP1, alongside a homology-directed repair (HDR) template. This template contained 800-bp DRP1 homology arms flanking the HALO-tag/mEGFP expression cassette, enabling precise integration directly upstream of the start codon. Genome-edited single-cell clones were subsequently isolated and validated through sequencing of the DRP1 locus, immunoblotting and fluorescence microscopy. For sgRNA design and cloning, sgRNAs targeting the human DRP1 locus (ENSG00000087470) near the start of the DRP1 open reading frame were designed using the CRISPR guide RNA design tools Benchling and CRISPOR. Candidate 20-bp sgRNAs with an NGG protospacer adjacent motif were filtered based on predicted on-target^58^ and off-target^59^ scores, using the human reference genome GRCh38 (hg38, *Homo sapiens*, Genome Reference Consortium), while accounting for known genome variants (dbSNP148)^60^. The selected sgRNA (sgRNA6: 5′-CGCCGGCCACGGCAATGAAT-3′) was cloned into the SpCas9-2A-GFP transfer vector using a corresponding synthetic DNA oligonucleotide set (sgRNA6-forward: 5′-caccgCGCCGGCCACGGCAATGAAT-3′ and sgRNA6-reverse: 5′-aaacATTCATTGCCGTGGCCGGCGc-3′).

Homology regions flanking the DRP1 locus (800 bp upstream and downstream of the start codon, excluding the start codon itself) were amplified from genomic DNA isolated from WT U2OS cells. These fragments were cloned into the pUC19 vector (Addgene #50005) using Gibson assembly, incorporating a multiple cloning site between the homology arms. The mEGFP sequence, including its start codon and a (GGS)_3_ linker, was amplified from pEGFP-N1 and inserted between the homology arms in the pUC19-DRP1-HDR vector via restriction cloning.

WT U2OS cells were transfected with the SpCas9-DRP1-gRNA7 construct and co-transfected with 2 µg of the pUC19-DRP1-mEGFP-HDR construct via electroporation. The following day, cells were analysed by flow cytometry. Individual GFP-positive cells were sorted, with gating applied to exclude dead cells using the 4′,6-diamidino-2-phenylindole (DAPI) channel. Single-cell clones were expanded and validated by PCR genotyping of genomic DNA, immunoblotting and fluorescence microscopy. All newly generated cell lines are available from the corresponding author upon reasonable request.

### Western blot

Cells were collected using trypsin–EDTA (Sigma), resuspended in culture medium and collected by centrifugation at 300*g* for 5 min at 4 °C. The cell pellets were washed twice with ice-cold phosphate-buffered saline. For lysis, the pellets were resuspended in RIPA lysis buffer (50 mM Tris–HCl, pH 8.0, 150 mM NaCl, 1% (v/v) Triton X-100, 0.5% (w/v) sodium deoxycholate and 0.1% (w/v) sodium dodecyl sulfate (SDS)), incubated on ice for 20 min and then centrifuged at 20,000*g* for 20 min at 4 °C to remove cellular debris. Protein concentration was determined using the Bradford protein assay (Bio-Rad) according to the manufacturer’s protocol. A total of 50–100 µg of protein was boiled in SDS–polyacrylamide gel electrophoresis sample buffer (62.5 mM Tris–HCl, pH 6.8, 2% (w/v) SDS, 10% (v/v) glycerol, 0.005% (v/v) β-mercaptoethanol and 0.01% (w/v) bromophenol blue) for 5 min at 95 °C before SDS–polyacrylamide gel electrophoresis. Proteins were transferred to a nitrocellulose membrane (Trans-Blot Turbo, Bio-Rad), and equal sample loading was confirmed using Ponceau S staining.

The blots were washed with TBST (50 mM Tris–HCl, pH 7.5, 150 mM NaCl and 0.1% (v/v) Tween 20) and blocked with 5% (w/v) low-fat milk in TBST for 60 min. The membranes were then incubated with a 1:1,000 dilution of rabbit α-DRP1 primary antibody (DRP1 (D6C7) rabbit mAb #8570), rabbit α-MFF primary antibody (MFF pAb #17090-1-AP) or mouse α-FIS1 primary antibody (FIS1 mAb #sc-376469) in 3% (w/v) bovine serum albumin in TBST at 4 °C for 16 h. After three 5-min washes with TBST, the membranes were incubated with a 1:10,000 dilution of α-rabbit or α-mouse IgG-HRP secondary antibody (Jackson ImmunoResearch, #111-035-003 and #115-035-044) in 3% (w/v) bovine serum albumin in TBST for 1 h at room temperature.

The blots were washed three times with TBST and developed using SuperSignal West Pico PLUS chemiluminescent substrate (Thermo Scientific). Signals were detected either using a Fusion SL Gel Chemiluminescence Documentation System (Vilber Lourmat) or by exposure to Fujifilm medical X-ray film. Images were adjusted for brightness and contrast and cropped using Fiji/ImageJ^61^.

### Generation of DRP1 mutants by Q5 site-directed mutagenesis

The pACmEGFP-DNM1L and pACmCherry-DNM1L plasmids contain a cytomegalovirus (CMV) promoter and a kanamycin resistance cassette. The correct nucleotide sequences of the constructs were verified by DNA sequencing (Eurofins Genomics). Mutagenic primers were designed using the NEBaseChanger tool (New England Biolabs) and synthesized by Sigma-Aldrich. Single amino-acid substitutions were introduced into the pACmEGFP-DNM1L and pACmCherry-DNM1L plasmids using the Q5 Site-Directed Mutagenesis Kit (New England Biolabs), following the manufacturer’s instructions. In brief, the mutagenesis workflow included PCR amplification with Q5 Hot Start High-Fidelity DNA Polymerase, agarose gel electrophoresis to verify amplification, and a Kinase-Ligase-DpnI (KLD) reaction to circularize the amplified plasmid and remove the parental template DNA. The resulting plasmids were transformed into chemically competent *Escherichia coli* cells, followed by plasmid purification (miniprep) and verification of the desired mutations by DNA sequencing.

### Generation of CRISPR–Cas9 KO cell lines

KO cell lines were generated using CRISPR–Cas9 genome editing^59,62^. sgRNAs targeting the genes of interest were cloned into the px458 vector (Addgene #48138), which co-expresses Cas9 and GFP. Complementary oligonucleotides were annealed and ligated into BbsI-digested px458 following standard cloning procedures. The ligation product was transformed into DH5α competent *E. coli*, and colonies were selected on ampicillin plates. Correct insertion of the sgRNA was confirmed by Sanger sequencing using the U6 promoter forward primer (5′-GAGGGCCTATTTCCCATGATTC-3′). The resulting plasmids were transfected into the respective parental cell lines using Lipofectamine 2000 (Thermo Fisher Scientific) according to the manufacturer’s instructions. GFP-positive cells were isolated by fluorescence-activated cell sorting, and single cells were sorted into 96-well plates. Individual clones were expanded, and KO of the target gene was validated by Sanger sequencing and/or western blot analysis. The following sgRNAs were used: MFF KO: 5′-ctcagcgtaagtacacgagg-3′; MID49 KO: 5′-gggaagcggcgtagcgacga-3′.

### siRNA-mediated knockdown of MFF and FIS1

Knockdown of MFF and FIS1 was performed using small interfering RNA (siRNA) electroporation. Cells were seeded 2 days before transfection in six-well plates. For each condition, 1 × 10^6^ cells were collected and electroporated using the 4D-Nucleofector system (Lonza) with SE solution and program CM-104, following the manufacturer’s instructions. Cells were electroporated in 100 µl SE buffer containing siRNA at a final concentration of 40 nM. The following siRNAs were used: siMFF (Dharmacon, cat. no. L-018261-00-0005) and siFIS1 (Dharmacon, cat. no. L-020907-02-0005). After electroporation, cells were resuspended in complete growth medium and plated at approximately 1.5 × 10^5^ cells per well in six-well plates for western blot analysis. For microscopy experiments, ~1 × 10^5^ cells were seeded in 35-mm Ibidi imaging chambers (MatTek, #P356-1.5-14-C). Knockdown efficiency was maximal 72 h after electroporation, and cells were analysed at this timepoint.

### Sample preparation for SIM microscopy

All cells from the different cell lines were seeded in a glass-bottom imaging dish (MatTek, #P356-1.5-14-C) at a density of 10^5^ cells per dish 1 day before the experiment. Cells were stained with 100 nM MitoTracker Orange/Deep Red/Green (ThermoFisher, #M7510, #M22426, #M7514) for 20 min at 37 °C to visualize mitochondria. HALO–DRP1 was labelled with Janelia Fluor JFX650 HaloTag ligand (Promega, cat. no. HT1070) at 300 nM for 45 min at 37 °C in 5% CO_2_, followed by a 2 h washout in fresh medium before imaging. In addition, HeLa DRP1-KO cells were transfected with 50 ng mCherry–DRP1 and its mutant variants 2 days before microscopy.

### SIM microscopy

SIM imaging was performed on an Elyra 7 Lattice SIM2 inverted fluorescence microscope (Zeiss), which utilizes lattice patterns instead of grid lines. The microscope is equipped with a Plan Apochromat 63×/1.4 oil immersion objective, 488-nm and 561-nm optical pumped semiconductor lasers and a 642-nm diode laser, all maintained under temperature control at 37 °C with 5% CO_2_. Fluorescence emission was collected on two scientific complementary metal–oxide–semiconductor cameras in spectrally separated channels. For mitochondrial morphology measurements, *z*-stacks were acquired over a range of approximately 3 μm, with *z* resolution determined by the software algorithm to achieve maximum resolution with a minimal number of slices (Leap mode). For SPT measurements, time-lapse sequences were acquired for 30 s at a maximum time resolution of 112 ms per frame. Image channels were aligned using a transformation matrix generated from a calibration measurement with fluorescent beads. The raw image data were processed using the Zeiss SIM2 processing algorithm to generate high-resolution images, which were then adjusted for brightness and contrast using Fiji/ImageJ^61^.

Processed 2D SIM time series were imported into Imaris. DRP1 oligomers were segmented using the Surfaces module, and object centres were linked over time using the autoregressive motion model to generate trajectories. Tracks were rendered in 3D view for visualization. Additional microscope acquisition parameters and instrument performance information are provided in Supplementary Tables 1 and 2.

### SPT tracking with tubular model and MSD calculation

The Python script developed for this study is available via GitHub at https://github.com/YaPaKo/DRP1-Mito-Tracker. DRP1 tracking was analysed using a cylindrical coordinate model, suitable for the tubular mitochondrial network^48^. DRP1 positions were projected onto the tube, with a 180° angle limit due to 2D fluorescence microscopy data. Key parameters included DRP1’s *xy* coordinates, mitochondrial skeleton, radius and distance from DRP1 to the skeleton. The angular position was calculated as *ϕ* = arcsin(min(*d*, *r*)/*r*), where *d* is the distance to the skeleton and *r* is the radius. Positive and negative angles represent DRP1 on the left and right sides of the mitochondria, respectively, enabling trajectory analysis independent of mitochondrial shape.

The DRP1 signal was separated using thresholding algorithms from the scikit-image package^49^, with the Otsu method applied to isolate DRP1 oligomers on the outer mitochondrial membrane. The highest-intensity pixel within each oligomer was extracted per frame. Coordinates of DRP1 oligomers were linked across frames using the Hungarian algorithm^50^, with a displacement threshold of 15 pixels (~0.47 μm) and a tolerance for up to 5 missing points. Trajectories were assigned using the linear_sum_assignment algorithm (scipy)^51^.

The mitochondrial skeleton was generated by applying thresholding algorithms to the mitochondrial channel, followed by skeletonization^26^. The skeleton was smoothed using a seventh-order centred moving average, and junctions were removed. A vector and radius were calculated for each skeleton point in each frame.

DRP1 trajectories were projected onto the mitochondrial model by finding the nearest perpendicular skeleton point for each trajectory point. The cost function *c* = *d*(1 + |cos *θ*|) was used, where *θ* is the angle between the skeleton vector and the vector to the DRP1 point. The *z*-axis movement was determined from changes along the mitochondrial skeleton. DRP1 points outside the mitochondrial mask were adjusted using an offset from the Savitzky–Golay filter^38^.

Reliable trajectories were selected based on a minimum of 133 frames (~15 s) and perpendicularity to the mitochondrial skeleton (75° to 105°). Trajectories at mitochondrial ends or constriction sites were excluded, as well as those with a distance exceeding 5 pixels (~0.16 μm) from the skeleton.

Particle dynamics were quantified using the ensemble MSD. For each trajectory *i*, the squared displacement relative to the initial position was computed as $| {r}_{i}(t)-{r}_{i}(0){{\rm{| }}}^{2}$, where ${r}_{i}(t)$ denotes the particle position at time $t$. The MSD was then obtained by averaging over all trajectories,

$$\mathrm{MSD}(t)={{\langle }}| {r}_{i}(t)-{r}_{i}(0){| }^{2}{{{\rangle }}}_{i}.$$

Here, $t$ represents the elapsed time from the beginning of each trajectory. The resulting MSD curves were fitted to theoretical models describing distinct transport mechanisms. Diffusive dynamics were described by $\mathrm{MSD}(t)=4D{t}^{\alpha }$, where $D$ is the generalized diffusion coefficient and $\alpha$ is the anomalous diffusion exponent. Active transport was modelled as a combination of stochastic diffusion and persistent drift, $\mathrm{MSD}(t)=4{Dt}+{({vt})}^{2}$, where $v$ represents the drift velocity. Confined diffusion was described by $\mathrm{MSD}(t)={L}^{2}[1-\exp (-4{Dt}/{L}^{2})]$, where $L$ is the characteristic confinement length that determines the plateau value of the MSD at long times. Model parameters were estimated using nonlinear least-squares regression. Parameter uncertainties were estimated from the covariance matrix of the fit, and model selection was guided by goodness of fit and physical consistency with the observed trajectories.

To capture the observed super-diffusive-to-diffusive transition, MSD data were fitted with a two-regime model. For the short-time regime (*t* < 1 s), we used MSD(*τ*) = 4*D*_s_*t*^*α*^, where *α* quantifies anomalous diffusion (*α* > 1 indicates superdiffusion). For the long-time normal diffusion regime (*t* > 10 s), we used MSD(*t*) = 4*Dt*, after ensuring that in this regime α ≈ 1. Parameters were obtained via nonlinear least-squares fitting (SciPy 1.11.4), with uncertainties derived from the covariance matrix and reported as mean ± standard error of the mean (s.e.m.) across experimental replicates.

### Photoconversion and time-lapse imaging of mEOS–DRP1 oligomers

HeLa DRP1-KO cells were transiently transfected with mEOS–DRP1 using Lipofectamine 3000 according to the manufacturer’s instructions. Twenty-four hours after transfection, cells were seeded on live-cell imaging glass-bottom dishes (ibidi, μ-dish, 35 mm high) and incubated for an additional 24 h at 37 °C and 5% CO_2_ before imaging. For photoconversion experiments, cells were imaged in phenol red-free Dulbecco’s modified Eagle medium supplemented with 10% fetal bovine serum. DRP1 oligomers were photoconverted using a 405-nm laser pulse directed to the entire cell area. This selectively converted assembled DRP1 oligomers on the MOM from their native green fluorescence (excitation 488 nm) to a red-emitting form (excitation 561 nm), while the unassembled cytosolic DRP1 pool remained largely unconverted. Immediately after photoconversion (*t* = 0 s), confocal time-lapse imaging was performed using a Leica SP8 inverted confocal microscope equipped with 488-nm and 561-nm laser lines. Mitochondria were labelled with MitoTracker Deep Red (100 nM) added 30 min before imaging. Sequential images of the converted and unconverted DRP1 populations were acquired over time to monitor the stability of DRP1 oligomers and potential subunit exchange events. The green and red fluorescence intensities of individual DRP1 particles were quantified over time, and the red (561 nm) to green (488 nm) intensity ratio was determined for each tracked particle. Linear regression analysis was performed to evaluate the slope of intensity changes, allowing assessment of subunit exchange dynamics. Particles tracked for more than 20 s were included in the final analysis. Data were collected from at least nine independent cells in three biological replicates. Additional microscope acquisition parameters and instrument performance information are provided in Supplementary Tables 3 and 4.

### Generation of HeLa DRP1-KO doxycycline-inducible mEOS–DRP1 cells

To generate a doxycycline-inducible mEOS–DRP1 stable cell line, an mEOS-linker-DRP1 cassette was assembled into the pNTI821 Sleeping Beauty transposon vector (Addgene #216630) using PCR amplification followed by NEBuilder HiFi DNA Assembly into an SfiI-linearized vector. The construct was verified by Sanger sequencing. HeLa DRP1-KO cells were co-transfected with the pNTI mEOS-DRP1 plasmid and the SB100X transposase plasmid pCMV(CAT)T7-SB100 (Addgene #34879) using Lipofectamine 3000 according to the manufacturer’s instructions. Twenty-four hours after transfection, cells were selected with hygromycin for 4 days and subsequently cultured in antibiotic-free medium for 1 week. Single-cell clones were isolated by limiting dilution and screened for inducible expression. mEOS–DRP1 expression was induced by overnight treatment with doxycycline (1 µg ml⁻^1^) and verified by fluorescence microscopy and immunoblotting using anti-DRP1 antibodies.

### Molecular dynamics simulations

Coarse-grained molecular dynamics simulations were performed using GROMACS 2025.1 to investigate the surface diffusion of a semi-flexible polymer constrained to a rigid cylindrical scaffold. The model was designed to mirror experimental observations from single-molecule fluorescence microscopy, in which DRP1 filaments bind to and diffuse along the MOM. To match the experimental length scale, the cylindrical scaffold was assigned a diameter of 30 nm, corresponding to an approximate 1:16 scaling relative to in-cell imaging data. Polymer length was varied by adjusting the number of coarse-grained beads ($L$), spanning the range of filament lengths observed experimentally.

The cylindrical scaffold consisted of 27,000 beads arranged in a regular lattice with 2 nm interbead spacing, forming a smooth, impenetrable surface. All scaffold beads were harmonically restrained to their lattice positions ($k$= 10^6^ kJ mol⁻^1^ nm^−2^) to maintain structural rigidity while permitting unrestricted polymer diffusion along the surface. The polymer was represented as an $L$-bead chain with an equilibrium bond length of 1.5 nm. Interactions between beads were described using a modified Martini-style force field with pairwise Lennard-Jones potentials. Cylinder bead diameter (${\sigma }_{{\rm{C}}}$ = 1.0 nm), polymer bead diameter (${\sigma }_{{\rm{P}}}$ = 2.0 nm) and the cross-interaction parameter (${\sigma }_{{\rm{C}}-{\rm{P}}}$ = 1.5 nm) were defined using Lorentz–Berthelot combination rules to ensure appropriate excluded volume, stable surface confinement, and physically consistent bead sizes relative to the cylindrical geometry. Interaction energy parameters were set to ${k}_{\varepsilon ,{\rm{C}}}$ = 5.0 kJ mol⁻^1^, ${k}_{\varepsilon ,{\rm{P}}}$ = 1.0 kJ mol⁻^1^ and ${k}_{\varepsilon ,{\rm{C}}-{\rm{P}}}=\sqrt{5.0\times 1.0}\approx 2.23\,\mathrm{kJ}\,{\mathrm{mol}}^{-1}$. Polymer flexibility was systematically tuned using an angular potential with force constants ${k}_{\theta }$ ranging from 5 to 1,000 kJ mol⁻^1^ rad^−2^, and the polymer–surface interaction strength was independently modulated by varying ${k}_{\varepsilon ,{\rm{C}}-{\rm{P}}}$ across simulation sets.

All systems were energy-minimized using the steepest descent algorithm for up to 10,000 steps, with a convergence threshold of 100 kJ mol⁻^1^ nm⁻^1^. A conservative integration step size of 0.001 nm was employed to ensure numerical stability given the relatively large bead diameters. During minimization, scaffold beads were immobilized using GROMACS freeze-group functionality to prevent unphysical motion, while allowing the polymer to relax freely. Non-bonded interactions were evaluated using a 20 nm cut-off, and electrostatics were treated with a simple cut-off scheme appropriate for the uncharged coarse-grained system.

Stochastic (Langevin) dynamics were then employed to sample the canonical ensemble using the SD integrator. The equations of motion were integrated with a timestep of 0.1 ps, enabling stable propagation while capturing polymer relaxation dynamics. The friction coefficient was set to *g* = 5 ps^−1^, providing efficient conformational sampling without overdamping diffusive motion. Production simulations were run for 100 ns (5 × 10^7^ steps), ensuring sufficient sampling of polymer diffusion along the cylindrical surface and through constricted regions. Temperature was maintained at 330 K using a velocity-rescale thermostat with a coupling constant of 1.0 ps, and the entire system was coupled to a single thermostat to ensure uniform thermalization. Non-bonded interactions were computed using the Verlet cut-off scheme with neighbour lists updated every 20 steps; both van der Waals and electrostatic interactions were truncated at 1.0 nm, and a Verlet buffer tolerance of 0.005 kJ mol⁻^1^ pm⁻^1^ was applied to balance computational efficiency and interaction accuracy. Trajectory coordinates and energies were recorded every 100 ps, providing sufficient temporal resolution for diffusion analysis while maintaining manageable data volumes.

To probe the influence of geometric constriction on polymer dynamics, we additionally implemented a cylindrical channel featuring a smoothly varying radial constriction (neck) along its central axis. The total cylinder length was ${L}_{{\rm{c}}}=900\,\mathrm{nm}$, and the radius profile $R(z)$ was defined as a piecewise linear function of the axial coordinate $z$, with a minimum radius ${R}_{\min }$ at the neck centre (${z}_{{\rm{c}}}=450\,\mathrm{nm}$) and a maximum radius ${R}_{\max }=15.0\,\mathrm{nm}$ outside the neck region. This continuous profile ensured smooth first derivatives at the neck boundaries, avoiding force discontinuities. The cylinder was embedded in a periodic simulation box of 75.0 × 75.0 × 990.0 nm^3^, with the scaffold spanning $z=[\mathrm{45.0,}\,945.0]\,\mathrm{nm}$ to prevent spurious interactions between periodic images. The radial profile was defined as

$$R(z)=\left\{\begin{array}{cc}{R}_{\min }+({R}_{\max }-{R}_{\min })\frac{| z-{z}_{{\rm{c}}}| }{{N}_{{\rm{L}}}/2}, & | z-{z}_{{\rm{c}}}| < {N}_{{\rm{L}}}/2\\ {R}_{\max }, & \mathrm{otherwise},\end{array}\right.$$

where ${N}_{{\rm{L}}}$ defines the axial extent of the neck region.

Trajectories generated under periodic boundary conditions were unwrapped by correcting discontinuities whenever bead displacements exceeded half the box length in any spatial direction. To analyse polymer motion relative to the scaffold, Cartesian coordinates were transformed into a cylinder-centric reference frame. The local axial coordinate was defined as ${z}_{{\rm{local}}}=z-{z}_{{\rm{offset}}}$, aligning the scaffold with the interval $\left[0,|,{L}_{{\rm{c}}}\right]$. Angular coordinates were computed as $\theta (t)={\rm{atan}}2(y(t),x(t))$ and unwrapped using standard phase-unwrapping procedures to ensure continuity across $2{\rm{\pi }}$. Motion along the cylindrical surface was quantified using the arc-length coordinate $s(t)=R({z}_{{\rm{local}}}(t)){\theta}_{{\rm{unwrap}}}(t)$, which correctly maps angular displacements to physical distances on a non-uniform cylindrical surface.

Neck occupancy was quantified by defining a neck region ${z}_{{\rm{neck}}}=[{z}_{c}-\Delta z,\,{z}_{c}+\Delta z]$ with $\Delta z=2.6\,\mathrm{nm}$. From the unwrapped trajectories, we computed the fraction of time spent within this region, the distribution of continuous residence times, and the frequency of entries into the neck. Statistical uncertainties were estimated using block averaging with block sizes exceeding the autocorrelation time. Polymer surface mobility was characterized by calculating the MSD of the polymer COM. For each degree of freedom $\alpha \in \{z,s,{xy},3\mathrm{D}\}$, MSDs were computed as time-averaged squared displacements at lag time $\tau$, using corrected Cartesian coordinates for ${{\rm{MSD}}}_{{xy}}$ and arc-length displacements for ${\mathrm{MSD}}_{{\rm{s}}}$. Diffusion coefficients were extracted from the linear regime of the MSD curves, excluding early-time transients, using dimensionality-appropriate models: ${{\rm{MSD}}}_{z}(\tau )=2{D}_{z}\tau +{C}_{z}$, ${\mathrm{MSD}}_{{\rm{s}}}(\tau )=2{D}_{{\rm{s}}}\tau +{C}_{{\rm{s}}}$, ${{\rm{MSD}}}_{{xy}}(\tau )=4{D}_{{xy}}\tau +{C}_{{xy}}$ and ${\mathrm{MSD}}_{3\mathrm{D}}(\tau )=6{D}_{3\mathrm{D}}\tau +{C}_{3\mathrm{D}}$.

Together, this computational framework enables quantitative dissection of how polymer flexibility and confinement geometry regulate diffusion on cylindrical substrates, directly linking molecular-scale parameters to transport behaviours observed in mitochondrial systems.

### Co-diffusion analysis of DRP1 and MID49/51

To assess the dynamic coupling between DRP1 and MID49/51, we calculated the displacement cross-correlation function using the expression

$${C}_{{xy}}\left(\tau \right)={{\langle }}\Delta {{{\bf{r}}}_{1}}(t,\tau )\cdot \Delta {{{\bf{r}}}_{2}}(t,\tau ){{{\rangle }}}_{t},$$

where $\Delta {{{\bf{r}}}_{1}}\left(t\right)$ and $\Delta {{{\bf{r}}}_{2}}(t)$ represent the displacement vectors of DRP1 and MID49, respectively, and 〈⋅${\rangle }_{t}$ denotes the time average.

### Mitochondrial constriction quantification from 2D SIM live-cell time lapse

Live-cell time-lapse stacks were analysed in Fiji/ImageJ. For each fission event, two timepoints spanning prefission to postfission were selected. At each timepoint, a straight line region of interest (ROI; line width 5 pixels) was drawn perpendicular to the mitochondrial tubule at the prospective fission site and added to the ROI Manager. Line profiles were extracted for all timepoints simultaneously using ROI Manager → More → Multi Plot, exported via Data → List, and analysed offline. Mitochondrial diameter at the fission site was estimated as the full width at half maximum (FWHM) of the mitochondrial intensity profile (I_half = I_max/2; crossings determined by linear interpolation).

### Mitochondrial morphology analysis

Processed 3D images (*z*-stacks) acquired from live-cell SIM microscopy were adjusted for brightness and contrasts using Fiji/ImageJ. Cells that were not transfected with DRP1 were identified and excluded from the analysis. Image files were saved as ‘RGB color’ for MitoSkel analysis and changed to ‘8-bit’ for classification analysis. All mitochondrial morphology parameters, such as branch length, area, perimeter and circularity, were quantified using MitoSkel software^50^.

### Statistics and reproducibility

No statistical method was used to predetermine sample size. Sample sizes were based on standard practice in the field and previous studies. No data were excluded from the analyses. The experiments were not randomized, and no blinding was applied during data acquisition. Imaging-based analyses were conducted using standardized acquisition settings and automated or semi-automated analysis pipelines, minimizing potential bias. Whenever possible, data were analysed in a double-blinded manner to minimize confirmation bias. For mitochondrial morphology analysis of DRP1 mutants, image folders were anonymized and analysed using MitoSkel software in a blinded fashion. Effect sizes were not explicitly calculated, as comparisons focused on differences in distributions between groups and are supported by exact *P* values and sample sizes.

Statistical analyses and plots were generated using GraphPad Prism (version 8) and Origin (OriginLab, 2019). Data are presented as mean ± s.e.m. and derived from at least three independent experiments, unless otherwise stated. Data distribution was assessed using the Shapiro–Wilk test. For comparisons between two groups, normally distributed datasets were analysed using unpaired two-tailed Student’s *t*-tests, with Welch’s correction applied when variances were unequal. When normality assumptions were not met, two-tailed Mann–Whitney *U* tests were used.

Morphological parameters, including mean branch length, contour area, perimeter and circularity, were analysed as described above. Exact *P* values, sample sizes (*n*) and statistical tests are reported in the figure legends.

All experiments were independently repeated at least three times with similar results, unless otherwise stated.

### Reporting summary

Further information on research design is available in the Nature Portfolio Reporting Summary linked to this article.

## Online content

Any methods, additional references, Nature Portfolio reporting summaries, source data, extended data, supplementary information, acknowledgements, peer review information; details of author contributions and competing interests; and statements of data and code availability are available at 10.1038/s41556-026-01986-w.

## Supplementary information

Reporting Summary

Peer Review File

Supplementary Video 1 Two-dimensional SIM time-lapse imaging of U2OS mEGFP–DRP1 (magenta) and mitochondria labelled with MitoTracker (green). The dynamics of mEGFP–DRP1 on mitochondria reveal spiral-like trajectories. Images were acquired in a single *z*-plane using burst mode with a frame interval of 112 ms over a total duration of 30 s. Scale bar, 5 μm.

Supplementary Video 2 Two-dimensional SIM time-lapse imaging of U2OS mEGFP–DRP1 cells (magenta) and mitochondria labelled with MitoTracker (green). mEGFP–DRP1 particles continuously scan the mitochondrial network, with some mEGFP–DRP1 accumulating at discrete, immobile puncta that evolve into mitochondrial fission sites. Images were acquired in a single *z*-plane using burst mode with a frame interval of 112 ms. Scale bar, 1 µm.

Supplementary Video 3 Two-dimensional SIM time-lapse imaging of U2OS mEGFP–DRP1 cells (magenta) and mitochondria labelled with MitoTracker (green). mEGFP–DRP1 particles continuously scan the mitochondrial network accumulating at fission sites. After mitochondrial fragmentation, DRP1 remains distributed in roughly equal proportions on both mitochondrial tips. Images were acquired in a single *z*-plane using burst mode with a frame interval of 112 ms. Scale bar, 0.5 µm.

Supplementary Video 4 Three-dimensional SIM time-lapse imaging of U2OS HALO–DRP1 cells. DRP1 particles are indicated by grey spheres, and mitochondria are labelled with MitoTracker (magenta). HALO–DRP1 particles continuously scan the mitochondrial network along the lateral and longitudinal axes of the mitochondrion. Trajectories are colour-coded over time. Images were acquired as nine *z*-planes (*z*-step: 0.126 µm) with a frame interval of 4.47 s. Scale bar, 0.5 µm.

Supplementary Video 5 Three-dimensional SIM time-lapse imaging of U2OS HALO–DRP1 cells. DRP1 particles are indicated by grey spheres, and mitochondria are labelled with MitoTracker (magenta). HALO–DRP1 particles continuously scan the mitochondrial network along the lateral and longitudinal axes of the mitochondrion. Trajectories are colour-coded over time. Images were acquired as nine *z*-planes (*z*-step: 0.126 µm) with a frame interval of 4.47 s. Scale bar, 0.5 µm.

Supplementary Video 6 Two-dimensional SIM Video of a U2OS mEGFP–DRP1 cell showing mitochondria in magenta labelled with MitoTracker orange, mEGFP–DRP1 in green and actin in cyan, labelled with SiR actin. Scale bar, 0.5 µm.

Supplementary Video 7 Two-dimensional SIM time-lapse imaging of U2OS mEGFP–DRP1 cells (magenta) and mitochondria labelled with MitoTracker (green). SPT of individual mEGFP–DRP1 oligomers reveals trajectories of DRP1 rotating at the tip of mitochondria. Images were acquired in a single *z*-plane using burst mode with a frame interval of 112 ms. Trajectories are colour-coded by time as indicated. Scale bar, 2 µm.

Supplementary Video 8 Confocal time-lapse imaging of HeLa DRP1-KO cells transiently expressing mEOS–DRP1 (green/red) and stained with MitoTracker (cyan) to visualize mitochondria. The first frame is captured immediately after photoconversion of mEOS using targeted illumination at 405 nm to convert mitochondrial mEOS–DRP1 oligomers from green to red, while the cytosolic mEOS–DRP1 pool remains largely unconverted. Scale bar, 0.5 µm.

Supplementary Video 9 Representative video of a polymer diffusing on the cylindrical scaffold. *L* = 26, K_*θ*_ = 500 kJ mol^−1^ rad^−2^ and *K*_*ε*_ = 2.236 kJ mol^−1^.

Supplementary Video 10 Representative video of a polymer diffusing on the cylindrical scaffold with a neck with radius *r* = 7 nm, *L* = 26, *K*_*θ*_ = 100 kJ mol^−1 ^rad^−2^ and *K*_*ε*_ = 2.236 kJ mol^−1^.

Supplementary Video 11 Two-dimensional SIM time-lapse imaging of HeLa MID49 and DRP1 double KO transiently transfected with mEGFP–DRP1 (green) and mCherry–MID49 (magenta). SPT of individual mEGFP–DRP1 and mCherry–MID49 oligomers together with corresponding single-particle trajectories colour-coded by particle identity. Images were acquired in a single *z*-plane using burst mode with a frame interval of 112 ms. Scale bar, 0.3 µm.

Supplementary Table 1 Light microscopy reporting table for SIM acquisitions performed on the ZEISS ELYRA 7 system, including imaging settings, acquisition parameters, fluorophores, objectives, detectors and image processing details used in this study.

Supplementary Table 2 Laser power measurements and illumination settings used for SIM imaging experiments at different acquisition timepoints/wavelengths.

Supplementary Table 3 Light microscopy reporting table for confocal microscopy acquisitions, including microscope configuration, imaging parameters, fluorophores, objectives, detectors and image processing details used throughout the study.

Supplementary Table 4 Laser power measurements and illumination settings used for confocal imaging experiments at different acquisition timepoints/wavelengths.

## Source data

Source Data Figs. 1–7 and Extended Data Figs. 1–7 Source numerical data.

Source Data Unprocessed western blots.

## Data Availability

Source data are provided with this paper. All other data supporting the findings of this study are available from the corresponding author upon reasonable request.

## Code availability

The Python script developed for this study is available via GitHub at https://github.com/YaPaKo/DRP1-Mito-Tracker.
