# Supplementary material for: DRP1 and MID49 co-diffusion scans mitochondria for fission
Source: Nat Cell Biol. 2026 Jun 11;28(7):1393–407. doi: 10.1038/s41556-026-01986-w (PMC13364638; doi:10.1038/s41556-026-01986-w)
Supplement: Supplementary file 19 — Unprocessed western blots. [file 41556_2026_1986_MOESM19_ESM.pdf]

Supporting blots

U20S mEGFP DRP1 MFF KO
Figure 7i

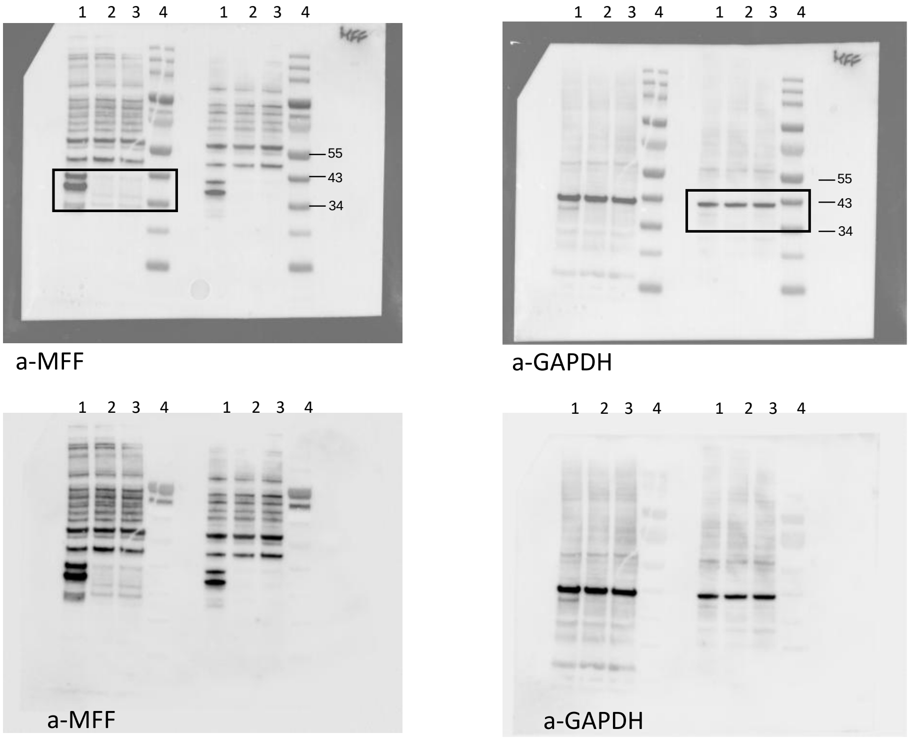

Lane 1: U20S mEGFP DRP1
Lane 2: U20S mEGFP DRP1 MFF KO#1
Lane 3: U20S mEGFP DRP1 MFF KO#2
Lane 4: Molecular weight marker

The square indicates the cropped region
shown in the figure.

U20S HALO-DRP1 GFP-Fis1 Fis1 KD

Figure 7k

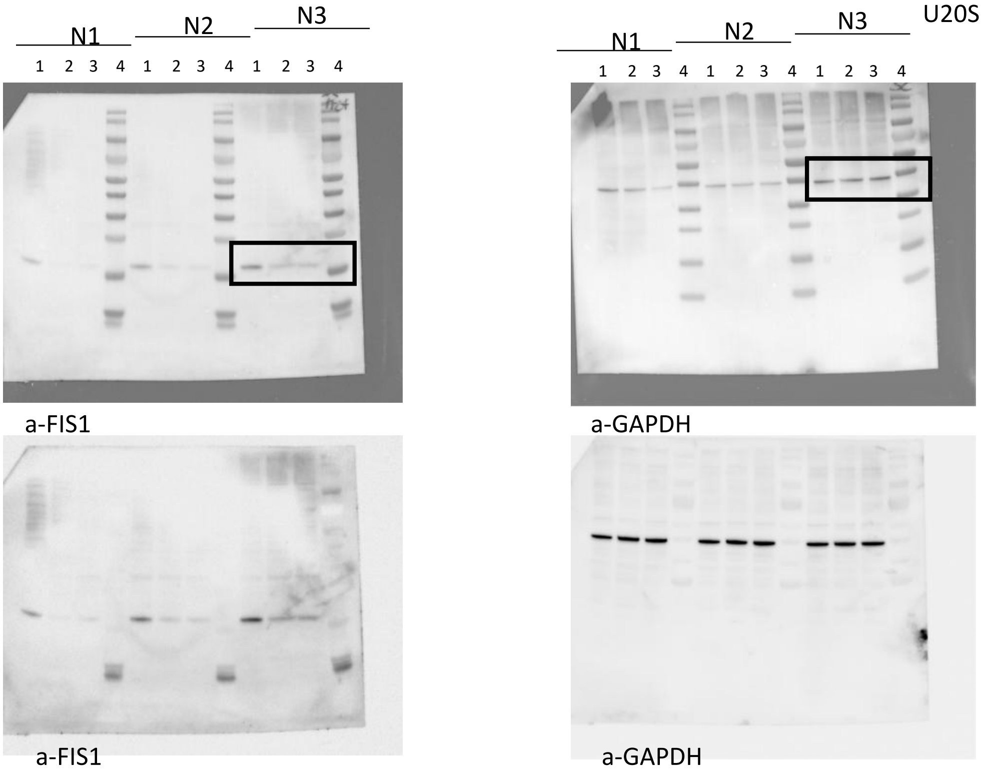

Lane 1: U20S HALO-DRP1

Lane 2: U20S U20S HALO-DRP1 Fis1KD #1

Lane 3: U20S HALO-DRP1 Fis1KD #2

Lane 4: Molecular weight marker

The square indicates the cropped region shown in the figure.

Generation of U2OS mEGFP DRP1

ED1b

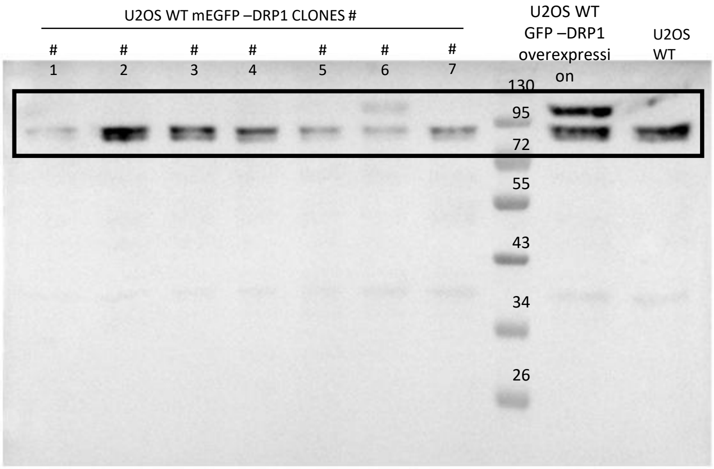

a-DRP1

80 kDa (DRP1)+27 kDa (GFP)≈107 kDa positive clone

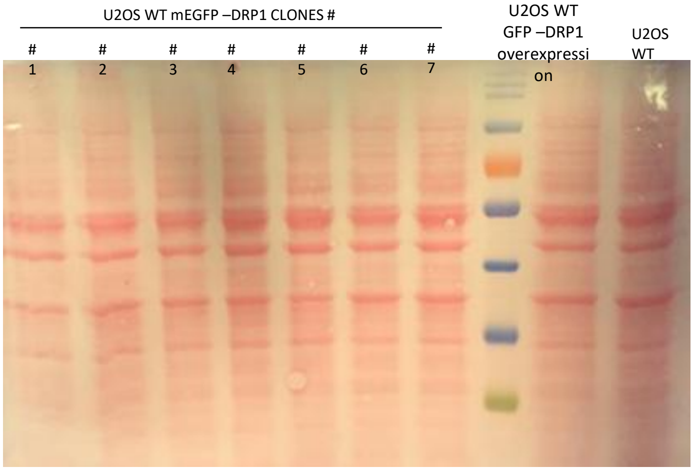

Ponceau S

The square indicates the cropped region shown in the figure.

Generation of U2OS HALO DRP1

Supplementary figure 1b

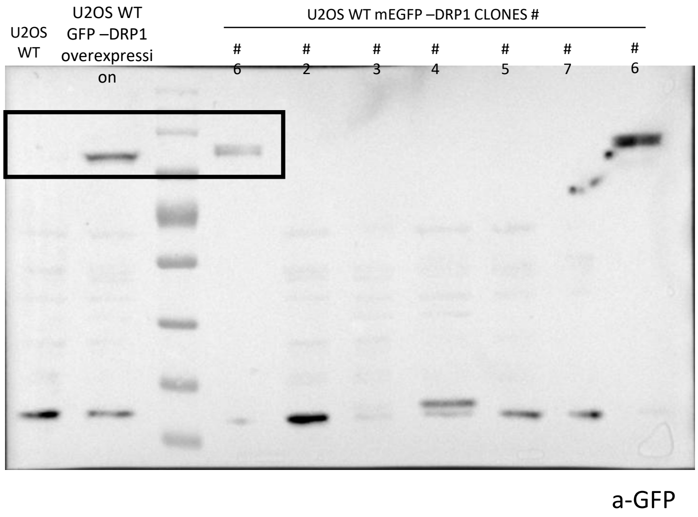

Generation of U2OS HALO DRP1

ED1c

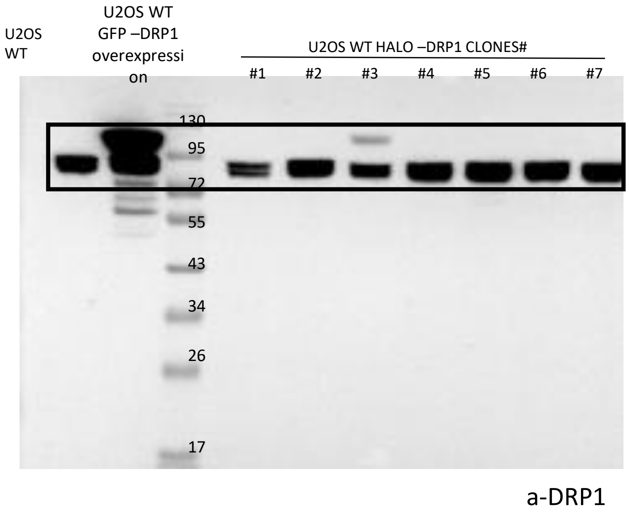

80 kDa (DRP1)+33 kDa (Halo)≈113 kDa positive clone

Square indicates cropped region displayed in the figure

HeLa mEOS Dox-inducible cell line
2 Clones with and wo Dox

ED4b

Exposure 1 Exposure 2 Exposure 3

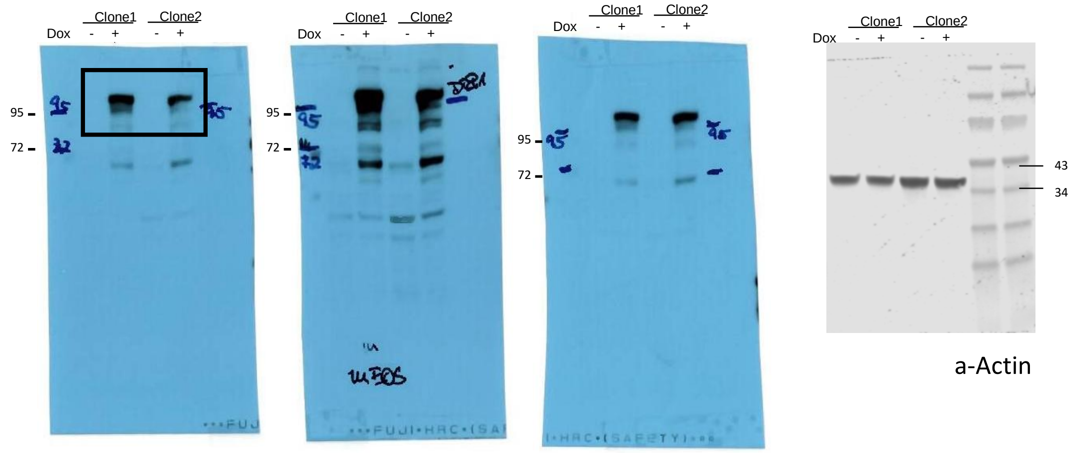

a-DRP1 a-DRP1 a-DRP1

80 kDa (DRP1)+27 kDa (mEos)≈107 kDa

Square indicates cropped region displayed in the figure

KD of MFF on U2OS mEGFP DRP1

ED7f

N1

KD of MFF

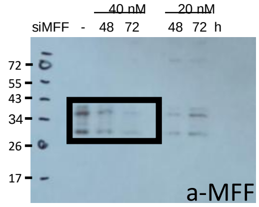

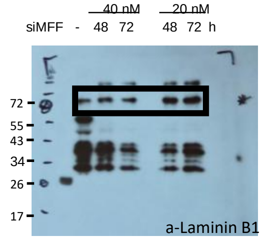

N2 + 3

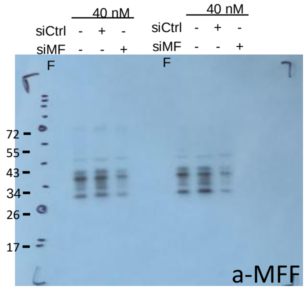

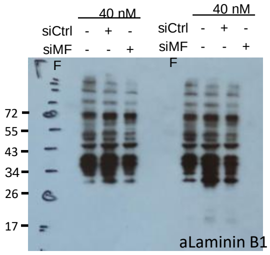

Square indicates cropped region displayed in the figure
