# Supplementary material for: DRP1 and MID49 co-diffusion scans mitochondria for fission
Source: Nat Cell Biol. 2026 Jun 11;28(7):1393–407. doi: 10.1038/s41556-026-01986-w (PMC13364638; doi:10.1038/s41556-026-01986-w)
Supplement: Supplementary file 2 — Peer Review File [file 41556_2026_1986_MOESM2_ESM.pdf]

Peer Review File

DRP1 and MID49 co-diffusion scans mitochondria for fission

Corresponding Author: Professor Ana García-Sáez

Version 0:

Decision Letter:

*Please delete the link to your author homepage if you wish to forward this email to co-authors.

Dear Professor García-Sáez,

Your manuscript, "DRP1 and MID49 co-diffusion scans mitochondria for fission", has now been seen by 3 referees, who are experts in mitochondrial dynamics (referee 1); DRP1 structure (referee 2); and particle tracking, MD simulations (referee 3). As you will see from their comments (attached below) they find this work of potential interest, but have raised substantial concerns, which in our view would need to be addressed with considerable revisions before we can consider publication in Nature Cell Biology.

Nature Cell Biology editors discuss the referee reports in detail within the editorial team, including the chief editor, to identify key referee points that should be addressed with priority, and requests that are overruled as being beyond the scope of the current study. To guide the scope of the revisions, I have listed these points below. We are committed to providing a fair and constructive peer-review process, so please feel free to contact me if you would like to discuss any of the referee comments further.

In particular, please focus on:

A) characterizing DRP1 scanning and identifying other components contributing to this activity

B) providing a detailed explanation of particle tracking methods and quantification

C) justifying the underlying parameters and output of the modelling as well as fully discuss limitations

D) providing evidence that DRP1 scanning/motion has a functional connection to fission

E) All other referee concerns pertaining to strengthening existing data, providing controls, methodological details, clarifications and textual changes, should also be addressed.

F) Finally please pay close attention to our guidelines on statistical and methodological reporting (listed below) as failure to do so may delay the reconsideration of the revised manuscript. In particular please provide:

- a Supplementary Figure including unprocessed images of all gels/blots in the form of a multi-page pdf file. Please ensure that blots/gels are labeled and the sections presented in the figures are clearly indicated.

- a Supplementary Table including all numerical source data in Excel format, with data for different figures provided as different sheets within a single Excel file. The file should include source data giving rise to graphical representations and statistical descriptions in the paper and for all instances where the figures present representative experiments of multiple independent repeats, the source data of all repeats should be provided.

We would be happy to consider a revised manuscript that would satisfactorily address these points, unless a similar paper is published elsewhere, or is accepted for publication in Nature Cell Biology in the meantime.

When revising the manuscript please:

- ensure that it conforms to our format instructions and publication policies (see below and https://www.nature.com/nature/for-authors).

- provide a point-by-point rebuttal to the full referee reports verbatim, as provided at the end of this letter.

- for any revision that includes light microscopy data, we ask our authors to please include a completed light microscopy reporting table https://www.nature.com/documents/Light_microscopy_reporting_table.xlsx to ensure the methods are described thoroughly. The table will be available to reviewers and ultimately published should the manuscript be accepted at the journal.

- provide the completed Reporting Summary (found here https://www.nature.com/documents/nr-reporting-summary.pdf). This is essential for reconsideration of the manuscript will be available to editors and referees in the event of peer review. For more information see http://www.nature.com/authors/policies/availability.html or contact me.

When submitting the revised version of your manuscript, please pay close attention to our href="https://www.nature.com/nature-portfolio/editorial-policies/image-integrity">Digital Image Integrity Guidelines. and to the following points below:

-- that unprocessed scans are clearly labelled and match the gels and western blots presented in figures.
-- that control panels for gels and western blots are appropriately described as loading on sample processing controls
-- all images in the paper are checked for duplication of panels and for splicing of gel lanes.

Finally, please ensure that you retain unprocessed data and metadata files after publication, ideally archiving data in perpetuity, as these may be requested during the peer review and production process or after publication if any issues arise.

Nature Cell Biology is committed to improving transparency in authorship. As part of our efforts in this direction, we are now requesting that all authors identified as 'corresponding author' on published papers create and link their Open Researcher and Contributor Identifier (ORCID) with their account on the Manuscript Tracking System (MTS), prior to acceptance. ORCID helps the scientific community achieve unambiguous attribution of all scholarly contributions. You can create and link your ORCID from the home page of the MTS by clicking on 'Modify my Springer Nature account'. For more information please visit please visit www.springernature.com/orcid.

This journal strongly supports public availability of data. Please place the data used in your paper into a public data repository, or alternatively, present the data as Supplementary Information. If data can only be shared on request, please explain why in your Data Availability Statement, and also in the correspondence with your editor. Please note that for some data types, deposition in a public repository is mandatory - more information on our data deposition policies and available repositories appears below.

Please submit the revised manuscript files and the point-by-point rebuttal to the referee comments using this link:

Link Redacted

*This url links to your confidential home page and associated information about manuscripts you may have submitted or be reviewing for us. If you wish to forward this email to co-authors, please delete the link to your homepage.

We would like to receive a revised submission within six months.

We hope that you will find our referees' comments, and editorial guidance helpful. Please do not hesitate to contact me if there is anything you would like to discuss.

Best wishes,

Angela Parrish

Angela R Parrish, PhD
Locum Senior Editor
Nature Cell Biology

Reviewers' Comments:

Reviewer #1 (Remarks to the Author):

Although both the importance of mitochondrial fission in organismal health and the key proteins involved in the process have long been established, the mechanism underlying mitochondrial fission is poorly understood. Here, the authors use sophisticated and rigorous approaches to address how DRP1- the GTPase responsible for mitochondrial fission — behaves at the outer mitochondrial membrane to drive fission.

To this end, the authors used live-cell structured illumination microscopy (SIM) in conjunction with sophisticated image analyses to visualize endogenously tagged DRP1-mEGFP at high temporal and spatial resolution. Using their imaging pipeline and various genetic approaches, the authors show that, contrary to the general perception, preformed DRP1 oligomers diffuse in helical trajectories across the outer mitochondrial membrane, which the authors term 'mito-scanning.' DRP1 mutants that disrupted diffusion led to decreased DRP1 motility along the OMM, and impaired fission. The authors also show that DRP1, form co-assemblies at the MOM with its co-adaptor, MID49, in accordance with previous in vitro studies. Disruption of these assemblies led to reduced DRP1 motility and more elongated mitochondria, in line with a reduction in mitochondrial fission. Thus, the authors propose that DRP1 motility on mitochondria is required for DRP1 to effectively scan the MOM for to-be fission sites, in a manner regulated by its interaction with Mid49. This is a very interesting paper, both for the data collected, tools generated, and for the originality and relevance of the hypothesis presented and supported. Below, I list a few points for the authors to consider:

Major points:
Support for 'scanning' model:
The authors propose that DRP1 diffuses along mitochondria and scans for fission sites that are marked by ER-actin-based constriction of mitochondria. The authors should provide evidence to support this — i.e. that the sites where DRP1 stalls corresponds to regions of constriction. The authors should also image DRP1 stall sites in conjunction with the ER or actin filaments.

Mito-scanner: DRP1 or MiD49, or MFF?
The bulk of the manuscript argues that DRP1 scans the mitochondria for fission sites. Towards the end, the authors provide evidence that MiD49 is essential for 'mito-scanning.' This raises two key questions the authors should address:
1) Is MiD51 the 'mito-scanner'? DRP1 may be diffusing along the OMM simply because it is complex with Mid51, and is therefore not doing any actual 'scanning'. Alternatively, DRP1-MID51 may be 'scanning' as a complex. To address this, the authors should compare MID51 motility in WT vs. DRP1 KO cells.
2) The deletion of MFF, as the authors write, produces the most severe fission defect (among the DRP1 receptors MID49, MID51 and FIS1). Indeed, the authors show that the DRP1 mutant (4KR) that has an increased interaction with MFF, leads to the most pronounced fission defect. The authors should test whether MFF may be involved in mito-scanning by comparing the motility of MFF with respect to DRP1.

Minor points/suggestions:
DRP1 also mediates peroxisomal fission. Do they think DRP1 behaves similary on peroxisomes. The authors should address this in the discussion.

A careful proofreading of the manuscript is recommended to address a few typographical or formatting issues and to enhance overall clarity.

Supplemental Figure S4b-c: Figures are cut off on the right side

Supplemental Video S5: A DRP1 assembly is evident at the end of the trace from the start while the DRP1 assembly from the start of the trace appears again at the same spot after supposedly moving. If true, this movie does not support the supposed movement of DRP1
DPR1 instead of DRP1 in abstract

Figure 1 is a bit cut off on the left side

Supplemental movie 1 and 2 appear to be the same

Supplemental figure S1d & S4d: wrote lenght instead of length on y axis (in fact length appears misspelled in most of figures)

Supplemental Figure S4 title is missing space between DRP1 and self-assembly

Figure 1: Color schematic to be consistent with fluorophore; i.e. green for mEGFP-DRP1

HeLa, not HELA

Consider adding 'sites' to end of title

Consider defining 'mito-scanning' at the start, and simply refer to the process as mito-scanning, rather than 'in a mito-scanner fashion'

Discussion: after reference 43 space needed.

Discussion: Could the authors touch upon other complexes they believe 'scan' membranes? For example?

Reviewer #1 (Remarks on code availability):

I am unable to assess the code.

Reviewer #2 (Remarks to the Author):

Review of "DRP1 and MID49 co-diffusion scans mitochondria for fission" by Zollo et al.

This is an interesting article from an established group in the mitochondrial dynamics field. In this manuscript, they suggest that mitochondrial fission operates as a diffusion-limited process that is regulated by MiD49. This is a novel finding backed with compelling videos of their microscopy sessions, clearly highlight movement of larger Drp1 species at the surface of mitochondria. The imaging aspects of the project are rigorous and generally clear and easy to follow. They supplment these data with simulations that are intended to define the type of movement observed (i.e. different types of diffusion), but the explanation and interpretation of this data is less clear and the parameters tested are limited, which also limited the interpretation. Additionally, the tags used to assess the movement of these complexes proximal to the mitochondrial are known to enhance oligomerization (Montecinos-Franjola 2020), which may bias these observations toward larger species that co-localize with the organelle. This is not cited nor ackowledge as a potential limitation of this study. The authors do a nice job of linking the role of MiD49 to this mito scanning, but they never exclude the possiblity of other partner proteins sharing this role, which limits their interpretation.

Overall, this is a novel and interesting finding in their study that adds to ourn understanding of dynamic protein interactions associated with organelle remodeling. Still, additional experiments and controls are needed to fully define the contributions of protein players potentially involved in these motions. Additionally, the limitations of their study design should be transparent. I don't think the use of tags detracts from their findings, but they may enhance their observation.

Specific comments and concerns are listed herein.

1. Are the authors confident that the Drp1 oligomers are scanning the tubular surface of mitochondria and not adjacent cytoskeletal elements. This distinction is important and there have been suggestions that Drp1 interactions with several types of filaments proximal to mitochondria. Complementary studies looking at microtubules and/or actin would be warranted.

2. It was previously reported that GFP-tagged Drp1 has a greater preponderance to assemble in cells and in vitro. As such, the construct expressed in U2OS cells would favor assembly. This may bias the results and interpretation. It is not clear whether this scanning by oligomers would be present with untagged protein that would be less oligomeric. One way to approach this would be to examine mutations on Drp1 that shift the oligomeric species that can be formed. Do these mutations that disrupt oligomerization affect this scanning?

3. Additionally, the mEOS experiments examining the exchance of oligmeric Drp1 with cytosolic is very interesting. But is this exchange limited by the preponderance of the tagger protein to form larger oligomers? This would seem to limit exchange.

4. The results demonstrating the equal partitioning of Drp1 to the tips of daughter mitochondria is interesting. If space is permitted, it would be worth bringing this data into a main panel in the manuscript so that it is not missed in the Supplemental Images. Given that the authors implicate MiD 49 in the scanning of Drp1 on the mitochondria, is there a molecular cue at sites where these oligomers "mature" into the fission complex? Specifically, are there other partner proteins present at these fission sites that would suggest synergy with MiD 49? Or is cardiolipin a trigger for assembly and constriction?

5. For the MSD calculation and representation, it is clear that the green fit line matches the black guideline. But it is unclear what this data might look like with confined or active diffusion. Can line representing these potential outcomes be plotted to demonstrate that the green fit line does not match the these other models.

6. A critical experiment was the removal MiD 49 to demonstrate impairment of this mito-scanner phenotype. I think it is equally important to knockdown Mff and Fis1 to determine the extent to which this one partner proteins is driving scanning. Related to this, Mid 51 would absolutely be examined.

7. Related to this point, is there a distinction in MiD49 and MiD51 that would prevent MiD51 from participating in the scanning of Drp1 oligomers? The double-laebling experiments nicely demonstrate the ability of Drp1 and MiD49 to interact, but both of these proteins are overexpressed in cells, which biases this interaction. Studies that eliminate a role for other partners in Drp1 scanning is still needed.

8. The interactions between MiD49 and Drp1 was found to build rigid filaments (Kalia et al), as noted in the authors' introduction. This type of rigid filament would limit diffusion, though MiD49 is implicated in facilitating scanning on the surface. This seems counterintuitive. Again, removing/depleting additional partners would be really helpful to delinate roles.

9. Within the course-grained simulations, the flexibility of the polymer sampling the surface of the tube is considered, but what governs the interactions with the tube? How is this meant to represent the surface of a mitochondrion? Most importantly, does the strength of this affinity for the tube impact the motion of these oliomers on the surface? I imagine that a

tighter interaction would limit motility and thereby restrict the conformational sampling of the oligomer. Another way to approach this might be to examine different lengths of polymers.

10. The authors speculate that MFF might first capture or recruit DRP1 oligomers from the cytosol, and then hand them over to MID49 (or MID51), where they might remain docked as part of complexes that continuous scan mitochondria for fission sites. There is evidence that MiDs prefer binding to larger oligomers of Drp1 and they actually impair Drp1 GTPase activation (Osellame 2016). In this way, MiD49 may prevent hydrolysis and thereby limit disassembly of the oligomers, pertmitting scanning. Conversely, Mff has been shown to augment Drp1 activity (Clinton 2016 and Liu 2021), which may promote constriction at defined sites of fission. Again, any experiment examining the interplay between Drp1 scanning and other partner proteins would greatly enhance the impact of this manuscript.

11. It is not clear in Figure 4C how these different representative images are different. The quantification is great, but any changes are unclear based on the images. Visual aids (star, arrowhead, etc) would be helpful in identifying attributes that should be highlighted.

Minor comments:

pg 3, line 58 - The description of post-translational modifications in Drp1 is a bit simplistic. There is tremendous diversity in the types of PTMs that impact Drp1 activity. I agree that this can be stated generally, but a more general review can be cited to better reference the types of PTM interactions involved.

Some of the formatting in the references appears to be off (i.e. extra spaces and special characters)

Figure 3 - Length is misspelled repeatedly

Reviewer #3 (Remarks to the Author):

This manuscript focuses on characterizing the motion of DRP1 oligomers on the mitochondrial surface, demonstrating that its motility is modulated by interactions with specific adaptors and that perturbing these interactions alters the network structure. The authors draw the conclusion that DRP1 motility promotes its ability to search for and accumulate at fission sites, and that fission rates may be limited by this search process. They use single particle tracking to describe the diffusivity of the oligomers, photoconversion to demonstrate they move as coherent units rather than treadmilling, and coarse-grained simulations to link oligomer shape with motility. While there is interesting data presented on DRP1 motility, I found the conclusions drawn from this study to be over-stretched and the quantitative analysis somewhat confusing. Specifically, the claim that DRP1 diffusive search is limiting for mitochondrial fission is not really supported by the data. Detailed comments are below.

Major issues:

1) The statistics calculated to characterize DRP1 motility are not well motivated. Throughout Figure 1, it is unclear if the oligomers are moving in a passive, diffusive-like fashion or in a processive `scanning' motion. The videos and individual trajectories indicate that there might be some directional processivity -- in which case, there needs to be an energy source for the motion and the authors should at least speculate as to what mechanism could lead to such directed motion. On the other hand, in Fig 2f, the scaling of the MSD appears to be diffusive even at short 0.1 sec (single frame?) timescales. If this is in fact the case then calculating a 'speed' for a processive particle is meaningless (the apparent speed will vary depending on the time separation used in the calculation). The authors need to clarify throughout Fig. 1 what mode of motion (diffusive or at least partially ballistic) they have in mind and to select an appropriate metric (diffusivity or mean squared step size if diffusive, speed if ballistic). Additionally, the text mentions that DRP1 exhibits 'spiral-like' motion but it was not clear where (if at all) this is shown in the data, nor what precisely is meant by 'spiral-like' motions. If the noisy-looking speed profiles shown in Fig 1 e-h are meant to support this observation, then it is unclear as to how. Claims of oscillatory motion would presumably require some spectral analysis of the velocity profiles (to show periodicity) in order to distinguish oscillation from random fluctuations.

Also, how the MSD is calculated should be better clarified. Is this MSD along the axis of the tube? Are trajectories unwrapped around the tube perimeter? If so, how do trajectories that go around full-circle contribute to the MSD?

2) The connection between DRP1 motion and its role as a fission factor remains tenuous. The only evidence for this seems to be that several mutations which perturb its interaction with mitochondrial proteins result in both changes in motion and changes in mitochondrial structure. However, the implied causality (``regulation of DRP1 scanning dynamics... modulates the correct efficiency of fission events'') is not directly shown. Also, the authors mention throughout the paper the idea that DRP1 scans for and then accumulates at fission sites, but the evidence for this is not clearly indicated in the figures. How many fission events are preceded by visible scanning motion of a DRP1 oligomer? Do the oligomers occur or accumulate preferentially on narrowed pre-pinched regions of the mitochondrial tubules? This ought to be measurable from the imaging data available. At the very least, the authors should make some estimates for what length of mitochondrial tubules is scanned by DRP1 prior to either finding a fission site or falling off the surface -- this would provide some rough indication as

to the importance of the scanning motion versus direct assembly in facilitating its search for a fission site.

3) The motivation for the simulation, its parameterization, and its relationship to the experimental data is unclear. Tubes of radius 1nm are on a completely different scale from mitochondria. If the goal is to simply rescale all lengths, since the mechanical and interaction parameters are arbitrary anyway, why not rename the length units to a more reasonable value? More importantly, what motivated the choice of relative size for the 1nm tubule and the 8nm(?) polymer. Are these reasonable proportions? What is the typical length of the DRP1 oligomer?
The finding that polymer diffusivity on a tubule is higher when the polymer is flexible enough to wrap around the tube is an interesting one -- however it is presumably very much dependent on the relative sizes of the polymer and the tube, and its relevance to the DRP1 story should be laid out more explicitly.

I was also unclear as to why the highly flexible polymer diffused more slowly. Where is the local entrapment coming from? More flexible (and hence more compact) polymers would generally diffuse faster if free in solution, so presumably this is due to some interaction with the surface. Could this effect be an artifact of representing the surface by discrete beads that are relatively sparsely placed (perhaps polymer beads are getting trapped in the gaps?).

To better connect the simulations with the rest of the results, it may be helpful to use them to reproduce some of the claimed observations in the experimental data. Do these simulated diffusing polymers exhibit the `spiral-like' or 'continuous oscillatory trajectory' motions claimed for DRP1? If constricted 'pre-fission' regions are included in the simulations, do the simulated polymers accumulate in such regions after an initial scanning period? And if so, do they show similar rotational movements when approaching a constriction, as noted for DRP1? A big question that remains unanswered after reading this manuscript is whether the observed intriguing movements of DRP1 are to be expected for a flexible structure diffusing passively on a surface, or whether they have to be attributed to some active as-yet unidentified processes driving the movement. Comparing passive diffusion simulations to the experimental observations may give some insight as to which is the case.

Minor comments:
- The authors should comment on the different slopes observed in Fig. 5A. What are the scaling exponents here? Are the less motile mutants showing more ballistic-like scaling?

- The word 'length' is misspelled throughout the figure labels

- What is mitochondrial circularity and perimeter and why is it relevant?

- In Fig 1c, it is not clear what is meant by the 'longitudinal length' L. Is this the length wrapped around the tube circumference or simply the distance from the axis?

- Bottom of pg. 8, Figure 3d-g is probably mean to refer to Fig S4

GUIDELINES FOR MANUSCRIPT SUBMISSION TO NATURE CELL BIOLOGY

READABILITY OF MANUSCRIPTS – Nature Cell Biology is read by cell biologists from diverse backgrounds, many of whom are not native English speakers. Authors should aim to communicate their findings clearly, explaining technical jargon that might be unfamiliar to non-specialists, and avoiding non-standard abbreviations. Titles and abstracts should concisely communicate the main findings of the study, and the background, rationale, results and conclusions should be clearly explained in the manuscript in a manner accessible to a broad cell biology audience. Nature Cell Biology uses British spelling.

MANUSCRIPT FORMAT – please follow the guidelines listed in our Guide to Authors regarding manuscript formats at Nature Cell Biology.

TITLE – should be no more than 100 characters including spaces, without punctuation and avoiding technical terms, abbreviations, and active verbs..

AUTHOR NAMES – should be given in full.

AUTHOR AFFILIATIONS – should be denoted with numerical superscripts (not symbols) preceding the names. Full addresses should be included, with US states in full and providing zip/post codes. The corresponding author is denoted by: "Correspondence should be addressed to [initials]."

ABSTRACT AND MAIN TEXT – please follow the guidelines that are specific to the format of your manuscript, as listed in our Guide to Authors (http://www.nature.com/ncb/pdf/ncb_gta.pdf) Briefly, Nature Cell Biology Articles, Resources and Technical Reports have 3500 words, including a 150 word abstract, and the main text is subdivided in Introduction, Results, and Discussion sections. Nature Cell Biology Letters have up to 2500 words, including a 180 word introductory paragraph (abstract), and the text is not subdivided in sections.

ACKNOWLEDGEMENTS – should be kept brief. Professional titles and affiliations are unnecessary. Grant numbers can be listed.

AUTHOR CONTRIBUTIONS – must be included after the Acknowledgements, detailing the contributions of each author to the paper (e.g. experimental work, project planning, data analysis etc.). Each author should be listed by his/her initials.

FINANCIAL AND NON-FINANCIAL COMPETING INTERESTS – the authors must include one of three declarations: (1) that they have no financial and non-financial competing interests; (2) that they have financial and non-financial competing interests; or (3) that they decline to respond, after the Author Contributions section. This statement will be published with the article, and in cases where financial and non-financial competing interests are declared, these will be itemized in a web supplement to the article. For further details please see https://www.nature.com/licenceforms/nrg/competing-interests.pdf.

REFERENCES – are limited to a total of 70 for Articles, Resources, Technical Reports; and 40 for Letters. This includes references in the main text and Methods combined. References must be numbered sequentially as they appear in the main text, tables and figure legends and Methods and must follow the precise style of Nature Cell Biology references. References only cited in the Methods should be numbered consecutively following the last reference cited in the main text. References only associated with Supplementary Information (e.g. in supplementary legends) do not count toward the total reference limit and do not need to be cited in numerical continuity with references in the main text. Only published papers can be cited, and each publication cited should be included in the numbered reference list, which should include the manuscript titles. Footnotes are not permitted.

METHODS – Nature Cell Biology publishes methods online. The methods section should be provided as a separate Word document, which will be copyedited and appended to the manuscript PDF, and incorporated within the HTML format of the paper.

Methods should be written concisely, but should contain all elements necessary to allow interpretation and replication of the results. As a guideline, Methods sections typically do not exceed 3,000 words. The Methods should be divided into subsections listing reagents and techniques. When citing previous methods, accurate references should be provided and any alterations should be noted. Information must be provided about: antibody dilutions, company names, catalogue numbers and clone numbers for monoclonal antibodies; sequences of RNAi and cDNA probes/primers or company names and catalogue numbers if reagents are commercial; cell line names, sources and information on cell line identity and authentication. Animal studies and experiments involving human subjects must be reported in detail, identifying the committees approving the protocols. For studies involving human subjects/samples, a statement must be included confirming that informed consent was obtained. Statistical analyses and information on the reproducibility of experimental results should be provided in a section titled “Statistics and Reproducibility”.

All Nature Cell Biology manuscripts submitted on or after March 21 2016 must include a Data availability statement as a separate section after Methods but before references, under the heading "Data Availability”. . For Springer Nature policies on data availability see http://www.nature.com/authors/policies/availability.html; for more information on this particular policy see http://www.nature.com/authors/policies/data/data-availability-statements-data-citations.pdf. The Data availability statement should include:

• Accession codes for primary datasets (generated during the study under consideration and designated as "primary accessions") and secondary datasets (published datasets reanalysed during the study under consideration, designated as "referenced accessions"). For primary accessions data should be made public to coincide with publication of the manuscript. A list of data types for which submission to community-endorsed public repositories is mandated (including sequence, structure, microarray, deep sequencing data) can be found here http://www.nature.com/authors/policies/availability.html#data.

• Unique identifiers (accession codes, DOIs or other unique persistent identifier) and hyperlinks for datasets deposited in an approved repository, but for which data deposition is not mandated (see here for details http://www.nature.com/sdata/data-policies/repositories).

• At a minimum, please include a statement confirming that all relevant data are available from the authors, and/or are included with the manuscript (e.g. as source data or supplementary information), listing which data are included (e.g. by figure panels and data types) and mentioning any restrictions on availability.

• If a dataset has a Digital Object Identifier (DOI) as its unique identifier, we strongly encourage including this in the Reference list and citing the dataset in the Methods.

We recommend that you upload the step-by-step protocols used in this manuscript to protocols.io. More details can found at https://www.protocols.io/help/publish-articles.

DISPLAY ITEMS – main display items are limited to 6-8 main figures and/or main tables for Articles, Resources, Technical Reports; and 5 main figures and/or main tables for Letters. For Supplementary Information see below.

FIGURES – Colour figure publication costs $600 for the first, and $300 for each subsequent colour figure. All panels of a multi-panel figure must be logically connected and arranged as they would appear in the final version. Unnecessary figures and figure panels should be avoided (e.g. data presented in small tables could be stated briefly in the text instead).

All imaging data should be accompanied by scale bars, which should be defined in the legend.
Cropped images of gels/blots are acceptable, but need to be accompanied by size markers, and to retain visible background signal within the linear range (i.e. should not be saturated). The boundaries of panels with low background have to be demarked with black lines. Splicing of panels should only be considered if unavoidable, and must be clearly marked on the figure, and noted in the legend with a statement on whether the samples were obtained and processed simultaneously. Quantitative comparisons between samples on different gels/blots are discouraged; if this is unavoidable, it should only be performed for samples derived from the same experiment with gels/blots were processed in parallel, which needs to be stated in the legend.

Figures should be provided at approximately the size that they are to be printed at (single column is 86 mm, double column is 170 mm) and should not exceed an A4 page (8.5 x 11"). Reduction to the scale that will be used on the page is not necessary, but multi-panel figures should be sized so that the whole figure can be reduced by the same amount at the smallest size at which essential details in each panel are visible. In the interest of our colour-blind readers we ask that you avoid using red and green for contrast in figures. Replacing red with magenta and green with turquoise are two possible colour-safe alternatives. Lines with widths of less than 1 point should be avoided. Sans serif typefaces, such as Helvetica (preferred) or Arial should be used. All text that forms part of a figure should be rewritable and removable.

We accept files from the following graphics packages in either PC or Macintosh format:

- For line art, graphs, charts and schematics we prefer Adobe Illustrator (.AI), Encapsulated PostScript (.EPS) or Portable Document Format (.PDF). Files should be saved or exported as such directly from the application in which they were made, to allow us to restyle them according to our journal house style.

- We accept PowerPoint (.PPT) files if they are fully editable. However, please refrain from adding PowerPoint graphical effects to objects, as this results in them outputting poor quality raster art. Text used for PowerPoint figures should be Helvetica (preferred) or Arial.

- We do not recommend using Adobe Photoshop for designing figures, but we can accept Photoshop generated (.PSD or .TIFF) files only if each element included in the figure (text, labels, pictures, graphs, arrows and scale bars) are on separate layers. All text should be editable in 'type layers' and line-art such as graphs and other simple schematics should be preserved and embedded within 'vector smart objects' - not flattened raster/bitmap graphics.

- Some programs can generate Postscript by 'printing to file' (found in the Print dialogue). If using an application not listed above, save the file in PostScript format or email our Art Editor, Allen Beattie for advice (a.beattie@nature.com).

Regardless of format, all figures must be vector graphic compatible files, not supplied in a flattened raster/bitmap graphics format, but should be fully editable, allowing us to highlight/copy/paste all text and move individual parts of the figures (i.e. arrows, lines, x and y axes, graphs, tick marks, scale bars etc.). The only parts of the figure that should be in pixel raster/bitmap format are photographic images or 3D rendered graphics/complex technical illustrations.

All placed images (i.e. a photo incorporated into a figure) should be on a separate layer and independent from any superimposed scale bars or text. Individual photographic images must be a minimum of 300+ DPI (at actual size) or kept constant from the original picture acquisition and not decreased in resolution post image acquisition. All colour artwork should be RGB format.

EXTENDED DATA FIGURES - When re-submitting your manuscript, please ensure that any supplementary figures and tables that are crucial to the manuscript's conclusions are converted into Extended Data figures and tables to increase visibility of these data. Extended Data figures and tables are online-only (present in the online PDF and full-text HTML versions of the paper), peer-reviewed display items that provide essential background to the article but are not included in the main article due to space constraints. A maximum of ten Extended Data display items (figures and tables) is permitted.

FIGURE LEGENDS – must not exceed 350 words for each figure to allow fit on a single printed NCB page together with the figure. They must include a brief title for the whole figure, and short descriptions of each panel with definitions of the symbols used, but without detailing methodology.

TABLES – main tables should be provided as individual Word files, together with a brief title and legend. For supplementary tables see below.

SUPPLEMENTARY INFORMATION – Supplementary information is material directly relevant to the conclusion of a paper, but which cannot be included in the printed version in order to keep the manuscript concise and accessible to the general reader. Supplementary information is an integral part of a Nature Cell Biology publication and should be prepared and

presented with as much care as the main display item, but it must not include non-essential data or text, which may be removed at the editor's discretion. All supplementary material is fully peer-reviewed and published online as part of the HTML version of the manuscript. Supplementary Figures and Supplementary Notes are appended at the end of the main PDF of the published manuscript.

Supplementary items should relate to a main text figure, wherever possible, and should be mentioned sequentially in the main manuscript, designated as Supplementary Figure, Table, Video, or Note, and numbered continuously (e.g. Supplementary Figure 1, Supplementary Figure 2, Supplementary Table 1, Supplementary Table 2 etc.).

Unprocessed scans of all key data generated through electrophoretic separation techniques need to be presented in a supplementary figure that should be labelled and numbered as the final supplementary figure, and should be mentioned in every relevant figure legend. This figure does not count towards the total number of figures and is the only figure that can be displayed over multiple pages, but should be provided as a single file, in PDF or TIFF format. Data in this figure can be displayed in a relatively informal style, but size markers and the figures panels corresponding to the presented data must be indicated.

The total number of Supplementary Figures (not including the “unprocessed scans” Supplementary Figure) should not exceed the number of main display items (figures and/or tables (see our Guide to Authors and March 2012 editorial http://www.nature.com/ncb/authors/submit/index.html#suppinfo; http://www.nature.com/ncb/journal/v14/n3/index.html#ed). No restrictions apply to Supplementary Tables or Videos, but we advise authors to be selective in including supplemental data.

Each Supplementary Figure should be provided as a single page and as an individual file in one of our accepted figure formats and should be presented according to our figure guidelines (see above). Supplementary Tables should be provided as individual Excel files. Supplementary Videos should be provided as .avi or .mov files up to 50 MB in size. Supplementary Figures, Tables and Videos much be accompanied by a separate Word document including titles and legends.

GUIDELINES FOR EXPERIMENTAL AND STATISTICAL REPORTING

REPORTING REQUIREMENTS – We are trying to improve the quality of methods and statistics reporting in our papers. To that end, we are now asking authors to complete a reporting summary that collects information on experimental design and reagents. The Reporting Summary can be found here https://www.nature.com/documents/nr-reporting-summary.pdf)If you would like to reference the guidance text as you complete the template, please access these flattened versions at http://www.nature.com/authors/policies/availability.html.

STATISTICS – Wherever statistics have been derived the legend needs to provide the n number (i.e. the sample size used to derive statistics) as a precise value (not a range), and define what this value represents. Error bars need to be defined in the legends (e.g. SD, SEM) together with a measure of centre (e.g. mean, median). Box plots need to be defined in terms of minima, maxima, centre, and percentiles. Ranges are more appropriate than standard errors for small data sets. Wherever statistical significance has been derived, precise p values need to be provided and the statistical test used needs to be stated in the legend. Statistics such as error bars must not be derived from n<3. For sample sizes of n<5 please plot the individual data points rather than providing bar graphs. Deriving statistics from technical replicate samples, rather than biological replicates is strongly discouraged. Wherever statistical significance has been derived, precise p values need to be provided and the statistical test stated in the legend.

Information on how many times each experiment was repeated independently with similar results needs to be provided in the legends and/or Methods for all experiments, and in particular wherever representative experiments are shown.

We strongly recommend the presentation of source data for graphical and statistical analyses as a separate Supplementary Table, and request that source data for all independent repeats are provided when representative experiments of multiple independent repeats, or averages of two independent experiments are presented. This supplementary table should be in Excel format, with data for different figures provided as different sheets within a single Excel file. It should be labelled and numbered as one of the supplementary tables, titled “Statistics Source Data”, and mentioned in all relevant figure legends.

--------- Please don't hesitate to contact NCB@nature.com should you have queries about any of the above requirements ---------

Version 1:

Decision Letter:

Our ref: NCB-A58645A

16th April 2026

Dear Dr. García-Sáez,

Thank you for submitting your revised manuscript "DRP1 and MID49 co-diffusion scans mitochondria for fission" (NCB-A58645A). I apologize for the delay in sharing our decision as a reviewer needed more time for re-review. Your revision has now been seen by the original referees and their comments are below. The reviewers find that the paper has improved in revision, and therefore we'll be happy in principle to publish it in Nature Cell Biology, pending minor revisions to satisfy the referees' final requests and to comply with our editorial and formatting guidelines.

The current version of your manuscript is in a PDF format; please email us a copy of the file in an editable format (Microsoft Word) as we cannot proceed with PDFs at this stage. Thank you in advance.

Once we have the Word file, we will be performing detailed checks on your paper and will send you a checklist detailing our editorial and formatting requirements in about 1-2 weeks. Please do not upload the final materials and make any revisions until you receive this additional information from us.

Thank you again for your interest in Nature Cell Biology. Please do not hesitate to contact me if you have any questions.

Sincerely,

Melina Casadio, PhD
Senior Editor, Nature Cell Biology
ORCID ID: https://orcid.org/0000-0003-2389-2243

Reviewer #1 (Remarks to the Author):

The authors have satisfactorily addressed all concerns. One remaining suggestion would be to include a quantification of the diameter of mitochondria lacking DRP1 to show the clear difference between constricting mitochondria versus to-be fissioned mitochondria (Fig. 1g). Congratulations on an exciting study!

Reviewer #2 (Remarks to the Author):

The authors have made substantial and thoughtful revisions in response to the initial round of reviews. The revised manuscript is significantly strengthened, with improved rigor, additional experimental support, and clearer interpretation of the data. In particular, the incorporation of new experiments addressing adaptor protein contributions and the expanded quantitative and simulation analyses meaningfully enhance the study’s impact and clarity.

The central model is now better supported by both experimental evidence and complementary simulations. The manuscript represents an important contribution to our understanding of mitochondrial dynamics.
Given the signification revisions and imporvements in the manuscript, I am in favor of acceptance.

Minor comment...

The authors may consider slightly tempering language around “scanning as rate-limiting,” unless explicitly quantified, although the new data substantially support its importance.

Reviewer #2 (Remarks on code availability):

I did a cursory review of the site and the code. We have not tried to implement it with any data

Reviewer #3 (Remarks to the Author):

The revised manuscript addresses my prior concerns.

In particular, the adjustment of the simulations to clearly defined relative units and the inclusion of constricted regions in the simulations, as well as clarifications on the passive nature of the Drp1 motion substantially strengthen the theory-experiment connection. Overall, this revised manuscript provides intriguing new observations on the mechanistic steps leading to Drp1 assembly as well as some interesting new results on the general behavior of long polymers diffusing over a tube with constrictions.

Version 2:

Decision Letter:

Dear Dr García-Sáez,

I am pleased to inform you that your manuscript, "DRP1 and MID49 co-diffusion scans mitochondria for fission", has now been accepted for publication in Nature Cell Biology.

Thank you for sending us the final manuscript files to be processed for print and online production, and for returning the manuscript checklists and other forms. Your manuscript will now be passed to our production team who will be in contact with you if there are any questions with the production quality of supplied figures and text.

Over the next few weeks, your paper will be copyedited to ensure that it conforms to Nature Cell Biology style. Once your paper is typeset, you will receive an email with a link to choose the appropriate publishing options for your paper and our Author Services team will be in touch regarding any additional information that may be required.

After the grant of rights is completed, you will receive a link to your electronic proof via email with a request to make any corrections within 48 hours. If, when you receive your proof, you cannot meet this deadline, please inform us at rjsproduction@springernature.com immediately.

You will not receive your proofs until the publishing agreement has been received through our system.

Due to the importance of these deadlines, we ask that you please let us know now whether you will be difficult to contact over the next month. If this is the case, we ask you provide us with the contact information (email, phone and fax) of someone who will be able to check the proofs on your behalf, and who will be available to address any last-minute problems.

If you have any questions about our publishing options, costs, Open Access requirements, or our legal forms, please contact ASJournals@springernature.com

Once your paper has been scheduled for online publication, the Nature press office will be in touch to confirm the details. An online order form for reprints of your paper is available at https://www.nature.com/reprints/author-reprints.html. All co-authors, authors' institutions and authors' funding agencies can order reprints using the form appropriate to their geographical region.

Publication is conditional on the manuscript not being published elsewhere and on there being no announcement of this work to any media outlet until the online publication date in Nature Cell Biology.

You may wish to make your media relations office aware of your accepted publication, in case they consider it appropriate to organize some internal or external publicity. Once your paper has been scheduled you will receive an email confirming the publication details. This is normally 3-4 working days in advance of publication. If you need additional notice of the date and time of publication, please let the production team know when you receive the proof of your article to ensure there is sufficient time to coordinate. Further information on our embargo policies can be found here: https://www.nature.com/authors/policies/embargo.html

Please note that *Nature Cell Biology* is a Transformative Journal (TJ). Authors may publish their research with us through the traditional subscription access route or make their paper immediately open access through payment of an article-processing charge (APC). Authors will not be required to make a final decision about access to their article until it has been accepted. Find out more about Transformative Journals

Authors may need to take specific actions to achieve compliance with funder and institutional open access mandates. If your research is supported by a funder that requires immediate open access (e.g. according to Plan S principles or the NIH public access policy) then you should select the gold OA route, and we will direct you to the compliant route where possible. Because authors warrant under our subscription licensing terms that they haven’t committed to licensing any version of their article under a licence inconsistent with the terms of our agreement – including the applicable embargo period – publication under the subscription model isn’t suitable for authors whose funders require no embargo.

To assist our authors in disseminating their research to the broader community, our SharedIt initiative provides you with a unique shareable link that will allow anyone (with or without a subscription) to read the published article. Recipients of the link with a subscription will also be able to download and print the PDF.

If your paper includes color figures, please be aware that in order to help cover some of the additional cost of four-color reproduction, Nature Portfolio charges our authors a fee for the printing of their color figures. Please contact our offices for exact pricing and details.

As soon as your article is published, you will receive an automated email with your shareable link.

If you have not already done so, we strongly recommend that you upload the step-by-step protocols used in this manuscript to protocols.io (https://protocols.io), an open online resource that allows researchers to share their detailed experimental know-how. All uploaded protocols are made freely available and are assigned DOIs for ease of citation. Protocols and Nature Portfolio journal papers in which they are used can be linked to one another, and this link is clearly and prominently visible in the online versions of both. Authors who performed the specific experiments can act as primary authors for the Protocol as they will be best placed to share the methodology details, but the Corresponding Author of the present research paper should be included as one of the authors. By uploading your Protocols onto protocols.io, you are enabling researchers to more readily reproduce or adapt the methodology you use, as well as increasing the visibility of your protocols and papers. You can also establish a dedicated workspace to collect your lab Protocols. Further information can be found at https://www.protocols.io/help/publish-articles.

You can use a single sign-on for all your accounts, view the status of all your manuscript submissions and reviews, access usage statistics for your published articles and download a record of your refereeing activity for the Nature Portfolio.

Nature Cell Biology encourages authors presenting evidence for cell, biological, molecular, and genetic interactions to consider communicating these findings using Biofactoid (https://biofactoid.org/). This tool helps users share a searchable representation of interactions (e.g. binding, gene expression, post-translational modification) between genes, gene products, or chemicals. Information added to Biofactoid, with author attribution, is shared on social media and public databases, such as Pathway Commons, where it can be discovered and analyzed in the context of a large and growing corpus of knowledge.

Please feel free to contact us if you have any questions.

With kind regards,

Melina Casadio, PhD
Senior Editor, Nature Cell Biology
ORCID ID: https://orcid.org/0000-0003-2389-2243

Click here if you would like to recommend Nature Cell Biology to your librarian
http://www.nature.com/subscriptions/recommend.html#forms

** Visit the Springer Nature Editorial and Publishing website at www.springernature.com/editorial-and-publishing-jobs for more information about our career opportunities. If you have any questions please click here.**

Open Access This Peer Review File is licensed under a Creative Commons Attribution 4.0 International License, which permits use, sharing, adaptation, distribution and reproduction in any medium or format, as long as you give appropriate credit to the original author(s) and the source, provide a link to the Creative Commons license, and indicate if changes were

made.
In cases where reviewers are anonymous, credit should be given to 'Anonymous Referee' and the source.
The images or other third party material in this Peer Review File are included in the article’s Creative Commons license, unless indicated otherwise in a credit line to the material. If material is not included in the article’s Creative Commons license and your intended use is not permitted by statutory regulation or exceeds the permitted use, you will need to obtain permission directly from the copyright holder.
To view a copy of this license, visit https://creativecommons.org/licenses/by/4.0/

Point-by-point answers to the reviewers

Reviewer #1 (Remarks to the Author):

Although both the importance of mitochondrial fission in organismal health and the key proteins involved in the process have long been established, the mechanism underlying mitochondrial fission is poorly understood. Here, the authors use sophisticated and rigorous approaches to address how DRP1- the GTPase responsible for mitochondrial fission — behaves at the outer mitochondrial membrane to drive fission.

To this end, the authors used live-cell structured illumination microscopy (SIM) in conjunction with sophisticated image analyses to visualize endogenously tagged DRP1-mEGFP at high temporal and spatial resolution. Using their imaging pipeline and various genetic approaches, the authors show that, contrary to the general perception, preformed DRP1 oligomers diffuse in helical trajectories across the outer mitochondrial membrane, which the authors term 'mito-scanning.' DRP1 mutants that disrupted diffusion led to decreased DRP1 motility along the OMM, and impaired fission. The authors also show that DRP1, form co-assemblies at the MOM with its co-adaptor, MID49, in accordance with previous in vitro studies. Disruption of these assemblies led to reduced DRP1 motility and more elongated mitochondria, in line with a reduction in mitochondrial fission. Thus, the authors propose that DRP1 motility on mitochondria is required for DRP1 to effectively scan the MOM for to-be fission sites, in a manner regulated by its interaction with Mid49. This is a very interesting paper, both for the data collected, tools generated, and for the originality and relevance of the hypothesis presented and supported. Below, I list a few points for the authors to consider:

We thank the reviewer for acknowledging the originality and novelty of our study, as well as the value of the data and tools generated.

Major points:

Support for 'scanning' model:

The authors propose that DRP1 diffuses along mitochondria and scans for fission sites that are marked by ER-actin-based constriction of mitochondria. The authors should provide evidence to support this — i.e. that the sites where DRP1 stalls corresponds to regions of constriction. The authors should also image DRP1 stall sites in conjunction with the ER or actin filaments.

We thank the reviewer for this suggestion. We have now quantified the mitochondrial diameter at the site of DRP1 stalling relative to the dynamics of DRP1 arrival and accumulation at these sites. As the reviewer notes, we find that the mitochondrial diameter is narrower at the sites where DRP1 accumulates, with about 180±20 nm, compared to 300-500 nm for unconstricted mitochondria[1]. These results strengthen our model and are shown in new Figure 1g.

We also developed a computational model to quantify how local geometric constrictions influence polymer dynamics, included in new Figure 4. The simulations show that even modest

perturbations of an otherwise cylindrical surface strongly reduce polymer mobility and promote localized accumulation and condensation of the polymer, in support of our model for DRP1 “mito-scanning”.

Finally, we attempted to image DRP1 stall sites together with actin filaments. To maintain compatibility with our live-cell super-resolution imaging conditions, we used SiR-actin, a far-red probe well suited for live-cell super-resolution microscopy due to its high brightness and photostability[2]. While genetically encoded probes such as LifeAct are widely used to visualize F-actin, they require genetic modification of cells and careful control of expression levels to avoid perturbing actin dynamics. For this reason, small-molecule probes such as SiR-actin are often preferred for live-cell imaging[3]. However, SiR-actin preferentially labels F-actin structures enriched near the cell periphery, and actin networks are highly abundant in U2OS cells, which complicates the visualization of mitochondrial-associated filaments in many regions of the cell. In addition, the combination of the high abundance of actin structures, the relatively low frequency of mitochondrial fission events, and the reduced temporal resolution associated with three-channel imaging (required to simultaneously visualize mitochondria, DRP1, and actin) makes precise temporal correlation between actin accumulation and DRP1 stalling difficult to establish in our dataset. This limitation is particularly relevant given the high temporal resolution required to visualize DRP1 mito-scanning dynamics. Similar experimental challenges in visualizing actin at mitochondrial fission sites have been reported previously[4]. A representative example is now included in Supplementary Figure S3 and supplementary video 6, together in an explanatory text in the main manuscript stating the limitations of this experiments.

Mito-scanner: DRP1 or MiD49, or MFF?

The bulk of the manuscript argues that DRP1 scans the mitochondria for fission sites. Towards the end, the authors provide evidence that MiD49 is essential for ‘mito-scanning.’ This raises two key questions the authors should address:

1) Is MiD51 the ‘mito-scanner’? DRP1 may be diffusing along the OMM simply because it is complex with Mid51, and is therefore not doing any actual ‘scanning’. Alternatively, DRP1-MID51 may be ‘scanning’ as a complex. To address this, the authors should compare MID51 motility in WT vs. DRP1 KO cells.

We thank the reviewer for raising this point. Following the reviewer’s suggestion, we performed live cell SIM imaging of mCherry-MID49 transiently expressed in HeLa WT and DRP1 knockout cells. Remarkably, we found that the presence of DRP1 influenced the mitochondrial distribution of mCherry-MID49, which appears as discrete foci in WT cells, but homogeneously distributed along the mitochondrial outer membrane in absence of DRP1. Single-particle tracking was therefore performed exclusively on MID49 foci in WT cells, where we found that these structures exhibit diffusion properties comparable to those of DRP1 assemblies. These results are now shown in new Figure 6.

We also performed two colour live cell SIM imaging experiments with mEGFP-DRP1 and mCherry-MID51. We found that DRP1/MID51 also co-diffused together as complexes, similar to MID49. However, the correlation analysis between the mEGFP-DRP1 and mCherry-MID51 signal was lower than for mCherry-MID49, indicating that a fraction of MID51 is not interacting

with DRP1 under our experimental conditions. These results are now shown in supplementary Figure S6.

Together, these new data indicate that MID49/51 alone is not the "mito-scanner", but that DRP1/MID49/51 scan mitochondria together as a complex. We have explained this now in the revised text.

2) The deletion of MFF, as the authors write, produces the most severe fission defect (among the DRP1 receptors MID49, MID51 and FIS1). Indeed, the authors show that the DRP1 mutant (4KR) that has an increased interaction with MFF, leads to the most pronounced fission defect. The authors should test whether MFF may be involved in mito-scanning by comparing the motility of MFF with respect to DRP1.

We thank the reviewer for this suggestion. To address this point, we performed live cell SIM imaging of transiently expressed mCherry-MFF in U2OS mEGFP-DRP1 knocked out in MFF. We found that mCherry-MFF mostly presents a homogeneous distribution suggesting of a lack of co-diffusion with mEGFP-DRP1 in discrete complexes. These results are included in new Figure 7.

In addition, to further understand the relationship between DRP1 and MFF with respect to the "mito-scanner" activity, we analyzed the dynamic behaviour of DRP1 under conditions of no interaction with MFF with live cell SIM imaging. For this, we used a mutant of mCherry-DRP1 that is deficient in MFF binding, R376E [5,6], transiently expressed in HeLa DRP1 KO cells. As an alternative approach, we also analyzed the dynamics of DRP1 endogenously tagged with mEGFP in U2OS cells in which we knocked down and knocked out MFF or not. In all cases, the mitochondrial localization of DRP1 was lost, and with that its "mito-scanner" activity, which was accompanied by an elongation of the mitochondrial network. These results are in line with a role of MFF in the recruitment of DRP1 to mitochondria, upstream of the "mito-scanner" activity, although they do not discard that continued interaction with MFF molecules contributes to the association with the mitochondrial surface also during the "scanning" process, thereby playing a role in it. These results are shown in Figure 7 and supplementary Figure S7.

Together, our results indicate that MFF is required for DRP1 recruitment to mitochondria, and with that, for the "mito-scanning" activity of DRP1. Yet, given the distinct mitochondrial distribution of MFF and DRP1/MID49/51 (MFF appears homogeneously distributed along the mitochondrial network in contrast to the discrete puncta of DRP1/MID49/51), our data rather suggest that MFF does not form part of these discrete "mito-scanning" complexes.

Minor points/suggestions:

DRP1 also mediates peroxisomal fission. Do they think DRP1 behaves similarly on peroxisomes. The authors should address this in the discussion.

This is a very interesting aspect. Given that peroxisomes have a different geometry, we would expect that the diffusion of DRP1 on the organelle surface may give rise to different trajectories. Alternatively, it is tempting to speculate that peroxisome elongation could influence DRP1 motility on the organelle and with that the fission process. Following the reviewer's suggestion, we have included these concepts in the discussion.

A careful proofreading of the manuscript is recommended to address a few typographical or formatting issues and to enhance overall clarity.

Following the reviewer's suggestion, we have thoroughly proofread the manuscript to eliminate typos and improve clarity.

Supplemental Figure S4b-c: Figures are cut off on the right side

We have adjusted this.

Supplemental Video S5: A DRP1 assembly is evident at the end of the trace from the start while the DRP1 assembly from the start of the trace appears again at the same spot after supposedly moving. If true, this movie does not support the supposed movement of DRP1.

We have exchanged the movie to show a clearer example.

DPR1 instead of DRP1 in abstract

We have corrected this.

Figure 1 is a bit cut off on the left side

We have adjusted this.

Supplemental movie 1 and 2 appear to be the same

We have checked that the movies 1 and 2 are correctly uploaded and named.

Supplemental figure S1d & S4d: wrote lenght instead of length on y axis (in fact length appears misspelled in most of figures)

We have corrected these typos throughout the manuscript.

Supplemental Figure S4 title is missing space between DRP1 and self-assembly

We have corrected this.

Figure 1: Color schematic to be consistent with fluorophore; i.e. green for mEGFP-DRP1

We thank the reviewer for this suggestion. We have updated the color scheme throughout the manuscript so that mEGFP–DRP1 is consistently shown in green whenever it appears in the same figures as mCherry-labeled proteins, ensuring fluorophore-consistent and intuitive color coding.

The only exceptions are as follows: In Figure 1, DRP1 is shown in magenta for the single-particle tracking analysis, as this representation provides optimal visualization of individual trajectories. In addition, whenever mEGFP-DRP1 is presented in the same figure as HALO–DRP1 labeled with JFX650, it is also displayed in magenta to maintain consistency in the color assignment for DRP1 across the figure.

HeLa, not HELA

We have corrected this.

Consider adding 'sites' to end of title

We thank the reviewer for this suggestion. After considering this option, we have opted to leave the title as it is.

Consider defining ‘mito-scanning’ at the start, and simply refer to the process as mito-scanning, rather than ‘in a mito-scanner fashion’

Following the reviewer’s suggestion, we have now defined “mito-scanning” in the abstract and introduction.

Discussion: after reference 43 space needed.

We have corrected this.

Discussion: Could the authors touch upon other complexes they believe ‘scan’ membranes? For example?

This is an interesting point. It is tempting to speculate that other GTPases of the dynamin family might exhibit scanning-like motility as part of their function in membrane fission and fusion. The supercoiling of lipid nanotubes by dynamin reported by De Camilli and colleagues in 2008 was interpreted as a rotatory movement of the helix turns for the best understood protein in the family[7]. Although live imaging data with sufficient spatiotemporal resolution are lacking for Mitofusins, a recent study reports their enrichment at discrete sites involved in membrane fusion and fission[8]), which would be compatible with a similar dynamic behavior. Based on our model, we would expect that the geometry of the membrane would strongly influence the resulting motility of the dynamin complex diffusing on it, as we mention above for peroxisomes. Visualization of the dynamics of oligomers of the dynamin family at high spatiotemporal resolution will help elucidate this question. In contrast, a treadmilling rotating motion around the bacterial septum that marks the site for membrane fission has been reported for the tubulin homolog FtsZ [9,10],which mediates bacterial cell division, indicating that alternative modes of motion can also be used for promoting membrane fission reactions. We have now elaborated on this aspect in the discussion.

Reviewer #2 (Remarks to the Author):

Review of "DRP1 and MID49 co-diffusion scans mitochondria for fission" by Zollo et al.

This is an interesting article from an established group in the mitochondrial dynamics field. In this manuscript, they suggest that mitochondrial fission operates as a diffusion-limited process that is regulated by MiD49. This is a novel finding backed with compelling videos of their microscopy sessions, clearly highlight movement of larger Drp1 species at the surface of mitochondria. The imaging aspects of the project are rigorous and generally clear and easy to follow. They supplment these data with simulations that are intended to define the type of movement observed (i.e. different types of diffusion), but the explanation and interpretation of this data is less clear and the parameters tested are limited, which also limited the interpretation. Additionally, the tags used to assess the movement of these complexes proximal to the mitochondrial are known to enhance oligomerization (Montecinos-Franjola 2020), which may bias these observations toward larger species that co-localize with the organelle. This is not cited nor ackowledge as a potential limitation of this study. The authors do a nice job of linking the role of MiD49 to this mito scanning, but they never exclude the possiblity of other partner proteins sharing this role, which limits their interpretation.

Overall, this is a novel and interesting finding in their study that adds to ourn understanding of dynamic protein interactions associated with organelle remodeling. Still, additional experiments and controls are needed to fully define the contributions of protein players potentially involved in these motions. Additionally, the limitations of their study design should be transparent. I don't think the use of tags detracts from their findings, but they may enhance their observation.

We thank the reviewer for acknowledging the novelty and interest of our study.

Specific comments and concerns are listed herein.

1. Are the authors confident that the Drp1 oligomers are scanning the tubular surface of mitochondria and not adjacent cytoskeletal elements. This distinction is important and there have been suggestions that Drp1 interactions with several types of filaments proximal to mitochondria. Complementary studies looking at microtubules and/or actin would be warranted.

We thank the reviewer for this suggestion. To address this question, we now provide a representative example of a 3D movie of DRP1 oligomers, in which individual DRP1 particles can be observed scanning around the mitochondrial tubule. These data are shown in Figure 1e and supplementary Figure S2e.

To further confirm that the DRP1 oligomers are scanning the tubular surface of mitochondria rather than adjacent cytoskeletal filaments, we performed complementary imaging of DRP1, mitochondria, and actin under our live-cell super-resolution imaging conditions. While simultaneous three-channel imaging imposes constraints on temporal resolution we did not observe DRP1 particles scanning along actin filaments in our dataset. A representative example of this experiment is now included in Supplementary Figure S3.

Our results are in line with previous studies specifically investigating the relationship between DRP1, actin, and mitochondria, that showed that DRP1 puncta at fission sites move along the mitochondrial outer membrane rather than along actin filaments[4]. Together, this report and our observations support the interpretation that the scanning behaviour we observe reflects DRP1 movement along the mitochondrial surface rather than along adjacent cytoskeletal structures.

2. It was previously reported that GFP-tagged Drp1 has a greater preponderance to assemble in cells and in vitro. As such, the construct expressed in U2OS cells would favor assembly. This may bias the results and interpretation. It is not clear whether this scanning by oligomers would be present with untagged protein that would be less oligomeric. One way to approach this would be to examine mutations on Drp1 that shift the oligomeric species that can be formed. Do these mutations that disrupt oligomerization affect this scanning?

We thank the reviewer for raising this point. The use of fluorescent labels is a requirement to visualize the dynamics of DRP1 with the high spatiotemporal resolution of our study. We agree with the reviewer that adding tags could alter the activity and dynamic behaviour of DRP1, as shown in Montecinos-Franjola et al. for mGFP[11]. In the revised manuscript, we now cite this article and acknowledge this as a potential limitation of our study.

We find similar behaviour for DRP1 tagged with GFP, mEOS or with HALO-tag. Although we cannot discard that the HALO- and mEOS-tags also shift DRP1 oligomerization, these results at least discard a GFP-specific effect. Together, our results suggest that even if the tag may bias the ratio of particles that are oligomerized, this does not seem to alter the general properties of their scanning behaviour.

3. Additionally, the mEOS experiments examining the exchange of oligomeric Drp1 with cytosolic is very interesting. But is this exchange limited by the preponderance of the tagger protein to form larger oligomers? This would seem to limit exchange.

Thank you for raising this point. As explained above, we cannot discard that tagging DRP1 with mEOS may alter the oligomerization and assembly/disassembly properties of DRP1, as reported for mEGFP[11]. We would not expect this effect to impact the ability of DRP1 oligomers to scan the mitochondrial surface observed in our study, since our data show that these potentially larger oligomers still scan the organelle without exchange. This is, the exchange of units is not necessary for the scanning. Yet, smaller oligomers not visualized in our study may exchange. In summary, from our data we cannot exclude a contribution of assembly/disassembly to DRP1 dynamics, which we know acknowledge in the text, toning down the conclusions accordingly.

4. The results demonstrating the equal partitioning of Drp1 to the tips of daughter mitochondria is interesting. If space is permitted, it would be worth bringing this data into a main panel in the manuscript so that it is not missed in the Supplemental Images.

Following the reviewer's suggestion, we now show these data in Figure 1 in the main article.

Given that the authors implicate MiD 49 in the scanning of Drp1 on the mitochondria, is there a molecular cue at sites where these oligomers "mature" into the fission complex? Specifically, are there other partner proteins present at these fission sites that would suggest synergy with MiD 49? Or is cardiolipin a trigger for assembly and constriction?

We thank the reviewer for this question. We propose that pre-constriction/membrane curvature act as such a cue for defining the sites where DRP1 matures into the fission complex, which we now strengthen with additional coarse-grained molecular dynamics simulations (see new Figure 4). In this case, it would be more of a structural cue.

While we cannot discard that other partner proteins or lipids might act as molecular cues (previous studies have proposed a role for ER–mitochondria contact sites, actin polymerization, activation of outer mitochondrial membrane receptors, and lipid composition such as cardiolipin enrichment[4,12–14]) the new experiments that we provide studying the role of MID51, MFF and FIS1 on DRP1 scanning dynamics do not directly implicate these proteins in defining the fission sites (see below).

Regarding cardiolipin, we could not envision a way of addressing this very challenging question experimentally: even if we detected altered DRP1 dynamics in cardiolipin depleted cells, this could be due to an indirect effect via the already established role of cardiolipin on mitochondria structure and function; also, fluorescently labelling cardiolipin as an alternative approach is known to alter its lipid properties, also confounding results.

5. For the MSD calculation and representation, it is clear that the green fit line matches the black guideline. But it is unclear what this data might look like with confined or active diffusion. Can line representing these potential outcomes be plotted to demonstrate that the green fit line does not match the these other models.

Thank you for this suggestion. In the revised manuscript, we now compare the fittings of the experimental data to models of free diffusion, confined diffusion and active transport, which is provided in supplementary Figure S4a. Fitting the average MSD curve to a confined diffusion model yielded confinement lengths far exceeding any physically relevant spatial scale of the system, effectively reducing the model to the free diffusion limit. Similarly, inclusion of a drift term in an active transport model did not produce a detectable quadratic scaling regime, and the fitted velocities were insufficient to generate ballistic behaviour within the experimental time window. In both cases, the alternative models collapse to normal diffusion, indicating that a simple diffusive description provides the minimal and physically consistent interpretation of the data. This comparative analysis reinforces that the most suitable model to describe DRP1 motility is free diffusion.

6. A critical experiment was the removal MiD 49 to demonstrate impairment of this mito-scanner phenotype. I think it is equally important to knockdown Mff and Fis1 to determine the extent to which this one partner proteins is driving scanning. Related to this, Mid 51 would absolutely be examined.

We thank the reviewer for raising this important point. Following the reviewer's suggestion, we have now performed new experiments of DRP1 motility under conditions in which the adaptors MFF, FIS1 and MiD51 have been depleted or their interactions with DRP1 abolished.

MFF: We analyzed the dynamic behaviour of DRP1 under conditions of no interaction with MFF with live cell SIM imaging. For this, we used a mutant of mCherry-DRP1 that is defficient in MFF binding, R376E[5,6]), transiently expressed in HeLa DRP1 KO cells. Under these conditions, we observed that disrupting the interaction between DRP1 and MFF directly affects DRP1 recruitment to mitochondria (Figure 7a). In contrast to WT DRP1, the DRP1 R376E mutant shows a more homogeneous cytosolic distribution, indicating impaired formation of discrete DRP1 foci. Consistent with this, cells expressing DRP1 R376E display a more elongated mitochondrial network, supporting the role of the DRP1–MFF interaction in promoting DRP1 accumulation at mitochondria and efficient mitochondrial fission. As an alternative approach, we also analyzed the dynamics of DRP1 endogenously tagged with mEGFP in U2OS cells in which we knocked down and knocked out in MFF. In both cases, the mitochondrial localization of mEGFP-DRP1 was lost, and with that its "mito-scanner" activity, which was accompanied by an elongation of the mitochondrial network. These results are in line with a role of the interaction with MFF, that is required for the recruitment of DRP1 to mitochondria, upstream of the "mito-scanner" activity. These results are shown in new Figure 7 and supplementary Figure S7.

To further test this relationship, we reintroduced fluorescently labelled MFF (mCherry-MFF) into U2OS mEGFP-DRP1 cells lacking MFF and performed live-cell SIM imaging. Under these conditions, DRP1 foci formation at the mitochondrial outer membrane was restored, confirming that MFF is required for efficient DRP1 recruitment and assembly on mitochondria. interestingly, mCherry-MFF mostly presents a homogeneous distribution in all tested

conditions suggesting of a lack of co-diffusion with DRP1 in discrete complexes. These results are included in new Figure 7.

FIS1: Using U2OS cells expressing endogenously tagged FIS1-EGFP and HALO-DRP1, we found that FIS1–EGFP displays a homogeneous mitochondrial distribution and does not form discrete sites along the mitochondrial surface, and that the two proteins do not co-localize together. Following the reviewer's suggestion, we also analyzed the dynamics of HALO-DRP1 in these cells and upon knockdown of the endogenously tagged FIS1-EGFP, which showed that depleting FIS1 had no significant effect on the diffusion coefficient of DRP1. Together, these results indicate that FIS1 does not contribute to DRP1 "mito-scanning" activity. These results are shown in new Figure 7.

MID51: Despite extensive attempts, we were unable to generate MID51 knockdown or knockout conditions in our U2OS mEGFP–DRP1 cells, which prevented a direct assessment of the effect of MID51 depletion on DRP1 scanning dynamics. We repeated the experiments ten times using two different delivery methods, electroporation and transient transfection with Lipofectamine 2000. In bulk populations, most cells died within one day after transfection. At the single-clone level, hundreds of surviving clones were screened, but none showed detectable MID51 depletion. As an alternative approach, we performed two-colour live-cell SIM imaging of mEGFP–DRP1 and mCherry–MID51. In these experiments, we observed that DRP1 and MID51 co-diffuse in dynamic complexes, similar to what we observed for MID49. However, correlation analysis between the mEGFP–DRP1 and mCherry–MID51 signals was lower than that observed for mCherry–MID49, indicating that a fraction of MID51 is not associated with DRP1 under our experimental conditions. This suggests that MID51 and MID49 may differ in their modes of interaction with DRP1. These results are now presented in Supplementary Fig. S6.

In summary, our new data define the contribution of DRP1 adaptors to its "mito-scanning" activity. They indicate that MFF is necessary for DRP1 association with mitochondria upstream of the scanning motion. Although MFF does not co-diffuse with DRP1 in discrete complexes, we cannot discard continued interaction between the proteins contributing to DRP1 association to mitochondria also during "mito-scanning". FIS1 did not colocalize with DRP1 and its depletion did not affect the diffusion of DRP1 particles, indicating that this protein does not contribute to the "mito-scanner" motion of DRP1.

In contrast, and consistent with a role in mitochondrial scanning, co-expression of mCherry–MID49 or mCherry-MID51 and mEGFP-DRP1 revealed co-diffusion in dynamic complexes. However, cross-correlation analysis yielded lower values for MID51 than those observed for MID49, suggesting differences in the interaction dynamics between DRP1 and the two receptors. These observations indicate that MID49 and MID51 may engage DRP1 through partially distinct mechanisms, a possibility that will require further investigation.

These results reveal distinct contributions of the DRP1 adaptors to the "mitoscanner" activity, with MID49 and MID51, but not MFF nor FIS1, directly associating with DRP1 in discrete co-diffusing complexes that regulate DRP1 motility.

7. Related to this point, is there a distinction in MiD49 and MiD51 that would prevent MiD51 from participating in the scanning of Drp1 oligomers? The double-laebling experiments nicely demonstrate the ability of Drp1 and MiD49 to interact, but both of these proteins are

overexpressed in cells, which biases this interaction. Studies that eliminate a role for other partners in Drp1 scanning is still needed.

We thank the reviewer for this comment. For comparison with MID49, we now performed two-color live cell SIM imaging of mCherry-MID51 and mEGFP-DRP1 transiently overexpressed in HeLa DRP1 KO (see supplementary Figure S6). Similar to MID49, we found that MID51 appeared in discrete complexes co-diffusing with DRP1, but remarkably, there was a fraction of MID51 that did not co-localize with DRP1, revealing different properties of MID49 and MID51 in their interaction with DRP1 at least under our experimental conditions. This raises interesting questions regarding the three-way interactions between DRP1/MID49/MID51 and how they are regulated, which we think go beyond the scope of this work, but that deserve attention in future studies.

While our experiments with WT DRP1 labelled with mEGFP or HALO-tag are performed in a cell line in which the protein is endogenously tagged, we agree that the two-color experiments with the DRP1 adaptors where performed under overexpression for technical reasons, which might bias the interactions. That said, similar two-color experiments with the different DRP1 adaptors provided different observations (MID51 partially colocalized with DRP1 in discrete complexes, while MFF and FIS1 did not), which supports the value of this system to study the association of DRP1 and its adaptors. Yet, we acknowledge this limitation in the revised manuscript.

8. The interactions between MiD49 and Drp1 was found to build rigid filaments (Kalia et al), as noted in the authors' introduction. This type of rigid filament would limit diffusion, though MiD49 is implicated in facilitating scanning on the surface. This seems counterintuitive. Again, removing/depleting additional partners would be really helpful to delinate roles.

This is an interesting point. We find our study highly complementary to the work by Kalia and colleagues. Their study critically advances our understanding of the structural organization of *in vitro* reconstituted DRP1 oligomers in complex with MID49 without addressing their dynamics in the cellular context, which our work tackles. Of note, the potential role of post-translational modifications or other cellular factors in the dynamics of the complexes and their regulation is not accounted for in the *in vitro* reconstituted system, which may underlie potential differences between both studies.

We have now extended the molecular dynamics simulations, which suggest is that there is non-linear effect of the stiffness of DRP1 oligomers on “mito- scanning” diffusion (see new Figure 4). At very low stiffness, the polymer adopts a compact conformation and diffuses rapidly. Increasing stiffness initially slows diffusion as the polymer extends, but further stiffening leads to an increase in diffusion coefficient. It is hard to estimate the stiffness or persistence length of the filaments reported by Kalia and coworkers, but we cannot discard that they might be within the same range. In this context, one could speculate that the decrease in persistence length of DRP1 assemblies in absence of MID49 could indeed be the reason why they are less efficient in “mito-scanning”. Another interesting possibility that opens based on the non-fully overlapping behaviour of MID49 and MID51 that we find (see new Figure 6 and supplementary Figure S6) is that the interplay between the two might modulate the stiffness of their complexes with DRP1. We have speculated about this in the revised discussion.

9. Within the course-grained simulations, the flexibility of the polymer sampling the surface of the tube is considered, but what governs the interactions with the tube? How is this meant to represent the surface of a mitochondrion? Most importantly, does the strength of this affinity for the tube impact the motion of these oliomers on the surface? I imagine that a tighter interaction would limit motility and thereby restrict the conformational sampling of the oligomer. Another way to approach this might be to examine different lengths of polymers.

We thank the reviewer for raising this point. We would like to note that the course-grained simulations provide a simplistic model to test whether diffusion of an oligomer on a cylindrical surface reproduces basic aspects of DRP1 motility in cells, as thus it does not capture all elements of the real situation. The interaction between the polymer and the cylindrical surface is controlled by the interaction force constant k_ϵ, aimed at mimicking the binding of DRP1 oligomers to mitochondrial tubules observed in our live cell imaging experiments. Following the reviewer suggestion, we have now extended our analysis to explicitly quantify the effect of k_ϵ on polymer diffusion. The new results indicate that for a polymer with fixed length and stiffness, increasing k_ϵ leads to a monotonic decrease in mobility, in agreement with the reviewer's expectation. This additional analysis confirms that polymer–surface adhesion is a key regulator of diffusion in our model. We have also tested the effect of different polymer lengths and found that the diffusion coefficient also decreases with the polymer length. These new results are included in Figure 4.

10. The authors speculate that MFF might first capture or recruit DRP1 oligomers from the cytosol, and then hand them over to MID49 (or MID51), where they might remain docked as part of complexes that continuous scan mitochondria for fission sites. There is evidence that MiDs prefer binding to larger oligomers of Drp1 and they actually impair Drp1 GTPase activation (Osellame 2016). In this way, MiD49 may prevent hydrolysis and thereby limit disassembly of the oligomers, pertmitting scanning. Conversely, Mff has been shown to augment Drp1 activity (Clinton 2016 and Liu 2021), which may promote constriction at defined sites of fission. Again, any experiment examining the interplay between Drp1 scanning and other partner proteins would greatly enhance the impact of this manuscript.

Thank you for sharing these interesting ideas. Following the reviewer's suggestion, we examined the interplay of DRP1 with its adaptors in experiments of co-localization and mEGFP-DRP1 diffusion analysis under conditions of adaptor depletion or co-expression of fluorescently tagged adaptors. We also analyzed MID49 dynamics under DRP1 depletion. Please see a summary of results obtained above. Based on these new results, we have refined our model for the mechanism of DRP1 "mito-scanning". Our data indicate that mCherry-MFF presents a different mitochondrial distribution compared to mEGFP-DRP1 and that it contributes to DRP1 recruitment to mitochondria. Since DRP1 recruitment is upstream of its scanning activity, from our data we cannot make conclusions on the potential role of MFF in promoting DRP1 constriction at fission sites. Yet, our results are in good agreement with a contribution of MID49/51 association with DRP1 oligomers and promotion of DRP1 "mito-scanning" activity, which could be associated with MID49 impairment of DRP1 GTPase activity leading to DRP1 oligomer stabilization and increase in stiffness. We have now included these considerations in the Discussion section.

11. It is not clear in Figure 4C how these different representative images are different. The

quantification is great, but any changes are unclear based on the images. Visual aids (star, arrowhead, etc) would be helpful in identifying attributes that should be highlighted.

Thank you for this suggestion. We show now zoomed-in regions of the cells for improved clarity.

Minor comments:

pg 3, line 58 - The description of post-translational modifications in Drp1 is a bit simplistic. There is tremendous diversity in the types of PTMs that impact Drp1 activity. I agree that this can be stated generally, but a more general review can be cited to better reference the types of PTM interactions involved.

Thank you for this suggestion. We have extended the text about the regulation of DRP1 by PMTs and included references.

Some of the formatting in the references appears to be off (i.e. extra spaces and special characters)

We have corrected this.

Figure 3 - Length is misspelled repeatedly

We have corrected this.

Reviewer #3 (Remarks to the Author):

This manuscript focuses on characterizing the motion of DRP1 oligomers on the mitochondrial surface, demonstrating that its motility is modulated by interactions with specific adaptors and that perturbing these interactions alters the network structure. The authors draw the conclusion that DRP1 motility promotes its ability to search for and accumulate at fission sites, and that fission rates may be limited by this search process. They use single particle tracking to describe the diffusivity of the oligomers, photoconversion to demonstrate they move as coherent units rather than treadmilling, and coarse-grained simulations to link oligomer shape with motility. While there is interesting data presented on DRP1 motility, I found the conclusions drawn from this study to be over-stretched and the quantitative analysis somewhat confusing. Specifically, the claim that DRP1 diffusive search is limiting for mitochondrial fission is not really supported by the data. Detailed comments are below.

We thank the reviewer for acknowledging the interest of our results.

Major issues:

1) The statistics calculated to characterize DRP1 motility are not well motivated. Throughout Figure 1, it is unclear if the oligomers are moving in a passive, diffusive-like fashion or in a

processive `scanning' motion. The videos and individual trajectories indicate that there might be some directional processivity -- in which case, there needs to be an energy source for the motion and the authors should at least speculate as to what mechanism could lead to such directed motion. On the other hand, in Fig 2f, the scaling of the MSD appears to be diffusive even at short 0.1 sec (single frame?) timescales. If this is in fact the case then calculating a 'speed' for a processive particle is meaningless (the apparent speed will vary depending on the time separation used in the calculation). The authors need to clarify throughout Fig. 1 what mode of motion (diffusive or at least partially ballistic) they have in mind and to select an appropriate metric (diffusivity or mean squared step size if diffusive, speed if ballistic). Additionally, the text mentions that DRP1 exhibits 'spiral-like' motion but it was not clear where (if at all) this is shown in the data, nor what precisely is meant by 'spiral-like' motions. If the noisy-looking speed profiles shown in Fig 1 e-h are meant to support this observation, then it is unclear as to how. Claims of oscillatory motion would presumably require some spectral analysis of the velocity profiles (to show periodicity) in order to distinguish oscillation from random fluctuations.

We thank the reviewer for raising this point. Based on our MDS analysis in Figure 2, we conclude that the motion of DRP1 is diffusive. Our intention in Figure 1 was to perform an agnostic description of DRP1 motility, where the focus was on reporting the motion of DRP1 along the mitochondrial network under steady state conditions, in contrast to the prevalent view in the field that DRP1 is directly recruited to mitochondrial fission sites from the cytosol. We agree with the reviewer that this could be confusing considering the conclusions about diffusive behavior from Figure 2 and for this reason, we have have now removed the speed analysis from Figure 1.

From the reviewer's comment, we understand that the description of DRP1 motion as in spiral trajectories was unclear. With this term we intended to describe that the net movement of DRP1 particles along the mitochondrial axis is often accompanied by rotation around that axis, so that the overall trajectory resembles a spiral (Figure 1d). We did not intend to claim any oscillatory or periodical motion. In our study, we propose that the trajectories observed result from diffusive motion of DRP1 particles on the tubular surface of the mitochondrion due to the coupling between the length of DRP1 oligomers, their flexibility, the strength of their interaction with the mitochondrial surface and the geometry of the mitochondrial surface. This model is supported by the coarse grained molecular dynamics simulations, including the new results. We have now described the trajectories as "spiral-like" and explained this better in the manuscript.

To improve clarity, we also provide now more and clearer examples of this type of "spiral-like" trajectories for DRP1 particle in Supplementary Figure 2 and provide a representative 3D example of the rotation of DRP1 particles around the mitochondrial axis to strengthen this point (Figure 1e and Supplementary Movies S4 and S5).

Also, how the MSD is calculated should be better clarified. Is this MSD along the axis of the tube? Are trajectories unwrapped around the tube perimeter? If so, how do trajectories that go around full-circle contribute to the MSD?

We thank the reviewer for this request. We have now explicitly detailed the MSD calculation protocol in the Methods section. Briefly, because MSD is computed for both experimental trajectories and simulations, two complementary approaches are used. For the experimental data, trajectories are analyzed in a two-dimensional (x, y) parametrization after drift correction.

For the simulations, particle coordinates are unwrapped to account for periodic boundary conditions along the cylindrical surface, ensuring that full rotations around the tube contribute continuously to the MSD rather than being artificially truncated. These procedures are now clearly described in the sections "SPT Tracking with Tubular Model and MSD Calculation" and "Molecular Dynamics Simulations."

2) The connection between DRP1 motion and its role as a fission factor remains tenuous. The only evidence for this seems to be that several mutations which perturb its interaction with mitochondrial proteins result in both changes in motion and changes in mitochondrial structure. However, the implied causality (``regulation of DRP1 scanning dynamics... modulates the correct efficiency of fission events'') is not directly shown. Also, the authors mention throughout the paper the idea that DRP1 scans for and then accumulates at fission sites, but the evidence for this is not clearly indicated in the figures. How many fission events are preceded by visible scanning motion of a DRP1 oligomer? Do the oligomers occur or accumulate preferentially on narrowed pre-pinched regions of the mitochondrial tubules? This ought to be measurable from the imaging data available. At the very least, the authors should make some estimates for what length of mitochondrial tubules is scanned by DRP1 prior to either finding a fission site or falling off the surface -- this would provide some rough indication as to the importance of the scanning motion versus direct assembly in facilitating its search for a fission site.

We thank the reviewer for their helpful suggestions how to better substantiate causality. Following the reviewer's suggestion, we have now monitored the dynamics of accumulation of DRP1 particles at fission sites followed by fission events. We have quantified whether DRP1 arrival at the fission sites takes places by visible scanning motion of DRP1 particles, or whether the particles are directly recruited to these sites (see new Figure 5j,k). From 65 events detected, we found that DRP1 exhibited scanning motion prior to accumulation at fission sites in 49 cases, while we observed direct DRP1 recruitment to the fission sites in 16 occasions. These results indicate that accumulation of DRP1 at fission sites through scanning motion takes places in a the majority of the cases, underscoring DRP1 scanning as a major path for DRP1 enrichment at these sites and strengthening the functional role of DRP1 mito-scanning on mitochondrial fission suggested by the results with DRP1 mutants and MID49 depletion.

Following the reviewer's suggestion, we have also quantified the diameter of the mitochondrial tubule at the fission site relative to the dynamics of stalling of DRP1 particles at that site. Remarkably, we observe that mitochondria are pre-constricted at fission sites prior to accumulation of DRP1 particles at these sites. These results are shown in new Figure 1g. We propose that the increased curvature at these pre-constricted sites promotes the stalling of DRP1 particles at the fission sites. This model is also supported by the new coarse-grained molecular dynamics simulations suggested by the reviewer (see below and new Figure 4).

Regarding the length of DRP1 trajectories along the mitochondrial tubule that are scanned prior to fission or to falling of the surface, we cannot provide an answer with strong statistics. The duration of the movies that we acquire with sufficient spatiotemporal resolution is limited by photobleaching of DRP1 particles and it is in general shorter than the trajectories of DRP1 particles on the mitochondrial surface. All the MSD calculations have been performed for several hundreds of trajectories longer than 15 s, indicating that scanning trajectories are often longer than this. However, fission events are very rare and we provide a time analysis for the few particles in which we could record the recruitment, scanning, stalling and fission in new Figure 5l, with trajectories that last around 100-700 of seconds (note, these movies were

collected at lower spatiotemporal resolution to extend the acquisition time and thus increase the chances of detecting fission events).

3) The motivation for the simulation, its parameterization, and its relationship to the experimental data is unclear. Tubes of radius 1nm are on a completely different scale from mitochondria. If the goal is to simply rescale all lengths, since the mechanical and interaction parameters are arbitrary anyway, why not rename the length units to a more reasonable value? More importantly, what motivated the choice of relative size for the 1nm tubule and the 8nm(?) polymer. Are these reasonable proportions? What is the typical length of the DRP1 oligomer?
The finding that polymer diffusivity on a tubule is higher when the polymer is flexible enough to wrap around the tube is an interesting one -- however it is presumably very much dependent on the relative sizes of the polymer and the tube, and its relevance to the DRP1 story should be laid out more explicitly.

We thank the reviewer for this comment. Our simulations are not intended to reproduce absolute mitochondrial dimensions, but rather the relative geometry experienced by DRP1 oligomers on mitochondrial tubules. To address this, in the revised manuscript we have now repeated the simulations framing the model in terms of relative length scales (see new Figure 4). In particular, we considered a ratio between the linearized DRP1 oligomer length (≈386 nm, measured experimentally; Figure 3g) while the mitochondrial diameter was measured to be approximately 500 nm from SIM images using Fiji. This corresponds to a ratio of ~0.8 between the DRP1 oligomer length and mitochondrial diameter, which is consistent with observed DRP1 assemblies in our data.

As noted by the reviewer, the effect of polymer stiffness on diffusion depends on the relative sizes of the polymer and the tube. To address this explicitly, we have now performed additional simulations for polymers of two different lengths while varying the angular force constant $k\theta$ (see new Figure 4b,c). In both cases, we observe the same qualitative non-monotonic dependence of diffusivity on stiffness, although the magnitude differs, with shorter polymers exhibiting higher diffusion coefficients. These new results show that our main conclusion is robust to changes in polymer length and strengthen its relevance to DRP1 dynamics.

I was also unclear as to why the highly flexible polymer diffused more slowly. Where is the local entrapment coming from? More flexible (and hence more compact) polymers would generally diffuse faster if free in solution, so presumably this is due to some interaction with the surface. Could this effect be an artifact of representing the surface by discrete beads that are relatively sparsely placed (perhaps polymer beads are getting trapped in the gaps?).

Thank you for this question. Precisely this point we find very interesting, as it is not a straight forward case of diffusion. This case involves 2D diffusion of an elongated particle on a curved surface. We argue that the relationship between the persistence length of the polymer and the curvature of the tube is important for the diffusion of the polymers on the surface. To strengthen this point, we have now performed additional coarse grained molecular dynamics simulations. The coupling between the relative geometries of the polymer and tubular surface is now also strengthened by the simulations including narrower tube necks of different diameters (new Figure 4g-k), which lead to polymer entrapment depending on the neck curvature, as well as by the simulations of polymers of different lengths with varying angular force constant $k\theta$ (Figure 4b,c), which show a complex non-monotonic behavior. This behavior contrasts with simple three-dimensional diffusion, where D_{iff} typically scales as Rg^{-1}, and highlights the

importance of anisotropic confinement on a curved surface. Our findings reveal a geometry-mediated diffusion mechanism that emerges from surface confinement.

To better connect the simulations with the rest of the results, it may be helpful to use them to reproduce some of the claimed observations in the experimental data. Do these simulated diffusing polymers exhibit the `spiral-like' or 'continuous oscillatory trajectory' motions claimed for DRP1?

Thank you for raising this point. To better connect the simulations with the experimental observations, we now include in Figure 4f and in Supplementary Movies 10 and 11 representative trajectories from systems exhibiting high and low diffusivities. These trajectories show that polymers undergoing coupled angular and longitudinal motion along the cylindrical surface result in spiral-like paths. This combined motion is consistent with the spiral-like trajectories observed experimentally for DRP1.

If constricted 'pre-fission' regions are included in the simulations, do the simulated polymers accumulate in such regions after an initial scanning period?

We thank the reviewer for this suggestion. In the revised manuscript, we extended our simulations to include cylinders with a localized constriction (“neck”), representing pre-fission mitochondrial regions. The neck geometry is defined by two parameters: the neck radius and the neck length. As shown in Figure 4g-k, we quantify both the polymer diffusion coefficient and the probability of escaping the neck region. Interestingly, we find that polymers preferentially accumulate within constricted regions after an initial scanning phase, and that even modest geometric constrictions strongly reduce diffusion and promote polymer trapping at the neck. These results support our model in which local mitochondrial geometry directly regulates DRP1 motility along the tubular network and stalling at pre-constricted fission sites.

And if so, do they show similar rotational movements when approaching a constriction, as noted for DRP1? A big question that remains unanswered after reading this manuscript is whether the observed intriguing movements of DRP1 are to be expected for a flexible structure diffusing passively on a surface, or whether they have to be attributed to some active as-yet unidentified processes driving the movement. Comparing passive diffusion simulations to the experimental observations may give some insight as to which is the case.

We thank the reviewer for bringing up this important point. Our passive diffusion simulations show that rotational and spiral-like motions naturally emerge as polymers approach and enter constricted regions, driven solely by geometric confinement. These results indicate that such movements can arise from passive diffusion on a curved surface and do not require active processes, indeed suggesting that the DRP1 motion observed in cells can be explained by passive diffusion of the oligomers interacting with the curved mitochondrial surface. That said, our results in cells suggest that receptors can additionally regulate DRP1 dynamics, which might happen by modulating the structure of the polymer (as speculated for MID49/51) or the strength of the interaction with the mitochondrial surface (as hypothesized for MFF). We clarify this in the Discussion of the revised manuscript.

Minor comments:

- The authors should comment on the different slopes observed in Fig. 5A. What are the scaling exponents here? Are the less motile mutants showing more ballistic-like scaling?

From the fittings we calculated scaling exponents of $\alpha \approx 0.5$ for WT cells, and $\alpha \approx 0.7$ for the S600A mutant, $\alpha \approx 0.6$ for the D221A and 4KR mutants. While both systems approach normal diffusive behavior at longer timescales ($\alpha \approx 1$), allowing a diffusion coefficient to be defined, their short-time dynamics slightly differ. In particular, WT cells exhibit stronger subdiffusion (lower α) compared to the mutants. This behavior likely reflects transient local constraints that hinder particle motion on short timescales, which are progressively relaxed as particles explore larger spatial regions, leading to effective diffusive transport at longer times.

- The word 'length' is misspelled throughout the figure labels

We have now corrected this.

- What is mitochondrial circularity and perimeter and why is it relevant?

Mitochondrial circularity describes how closely the shape of an individual mitochondrion approximates a perfect circle and is calculated as $C=4\pi A/P^2$ where *A* is the area and *P* is the perimeter of the mitochondrion. A value of 1 corresponds to a perfect circle, while lower values indicate more elongated or irregular shapes. Mitochondrial perimeter refers to the average contour length of the individual mitochondria. These parameters were extracted using the MitoSkel analysis pipeline[15], which performs automated segmentation and quantification of mitochondrial morphology. Together, these parameters are relevant because the provide an estimation about the general morphology of the mitochondrial network in a cell, with lower values of circularity and perimeter reflecting a more fragmented mitochondrial network. We have now explained this better in the methods section.

- In Fig 1c, it is not clear what is meant by the 'longitudinal length' L. Is this the length wrapped around the tube circumference or simply the distance from the axis?

We thank the reviewer for pointing out that this definition was unclear. We have corrected the description to specify that L corresponds to the distance between the central axis of the mitochondrial tube and the center of mass of each mEGFP-DRP1 particle.

- Bottom of pg. 8, Figure 3d-g is probably mean to refer to Fig S4

We have now corrected this in the new Figure order.

Reviewer #1:
Remarks to the Author:
The authors have satisfactorily addressed all concerns. One remaining suggestion would be to include a quantification of the diameter of mitochondria lacking DRP1 to show the clear difference between constricting mitochondria versus to-be fissioned mitochondria (Fig. 1g). Congratulations on an exciting study!
We thank the reviewer for this comment. We did not quantify the diameter of mitochondria lacking DRP1 because it would be a rather subjective process to identify the to-be fission sites without the protein accumulating there.

Reviewer #2:
Remarks to the Author:
The authors have made substantial and thoughtful revisions in response to the initial round of reviews. The revised manuscript is significantly strengthened, with improved rigor, additional experimental support, and clearer interpretation of the data. In particular, the incorporation of new experiments addressing adaptor protein contributions and the expanded quantitative and simulation analyses meaningfully enhance the study's impact and clarity.

The central model is now better supported by both experimental evidence and complementary simulations. The manuscript represents an important contribution to our understanding of mitochondrial dynamics.
Given the signification revisions and imporvements in the manuscript, I am in favor of acceptance.

Minor comment...

The authors may consider slightly tempering language around "scanning as rate-limiting," unless explicitly quantified, although the new data substantially support its importance.

We thank the reviewer for this suggestion. We have removed explicit references to "rate-limiting" in the text.

Remarks on code availability:
I did a cursory review of the site and the code. We have not tried to implement it with any data

Reviewer #3:
Remarks to the Author:
The revised manuscript addresses my prior concerns.

In particular, the adjustment of the simulations to clearly defined relative units and the inclusion of constricted regions in the simulations, as well as clarifications on the passive nature of the Drp1 motion substantially strengthen the theory-experiment connection. Overall, this revised manuscript provides intriguing new observations on the mechanistic steps leading to Drp1 assembly as well as some interesting new results on the general behavior of long polymers diffusing over a tube with constrictions.

References

1. Tábara, L. C., Segawa, M. & Prudent, J. Molecular mechanisms of mitochondrial dynamics. *Nat. Rev. Mol. Cell Biol.* **26**, (2024).

2. Lukinavičius, G. *et al.* A near-infrared fluorophore for live-cell super-resolution microscopy of cellular proteins. *Nature Chemistry 2013 5:2* **5**, 132–139 (2013).

3. Nasufovic, V. *et al.* SiR-XActin: A fluorescent probe for imaging actin dynamics in live cells. *bioRxiv* 2025.02.04.636537 (2025) doi:10.1101/2025.02.04.636537.

4. Ji, W. K., Hatch, A. L., Merrill, R. A., Strack, S. & Higgs, H. N. Actin filaments target the oligomeric maturation of the dynamin GTPase Drp1 to mitochondrial fission sites. *Elife* **4**, 1–25 (2015).

5. Clinton, R. W., Francy, C. A., Ramachandran, R., Qi, X. & Mears, J. A. Dynamin-related protein 1 oligomerization in solution impairs functional interactions with membrane-anchored mitochondrial fission factor. *Journal of Biological Chemistry* **291**, 478–492 (2016).

6. Strack, S. & Cribbs, J. T. Allosteric modulation of Drp1 mechanoenzyme assembly and mitochondrial fission by the variable domain. *Journal of Biological Chemistry* **287**, 10990–11001 (2012).

7. Roux, A., Uyhazi, K., Frost, A. & De Camilli, P. GTP-dependent twisting of dynamin implicates constriction and tension in membrane fission. *Nature 2006 441:7092* **441**, 528–531 (2006).

8. Gatti, P., Schiavon, C., Cicero, J., Manor, U. & Germain, M. Mitochondria-and ER-associated actin are required for mitochondrial fusion Check for updates. https://doi.org/10.1038/s41467-024-55758-x doi:10.1038/s41467-024-55758-x.

9. Bisson-Filho, A. W. *et al.* Treadmilling by FtsZ filaments drives peptidoglycan synthesis and bacterial cell division. *Science (1979).* **355**, 739–743 (2017).

10. Yang, X. *et al.* GTPase activity-coupled treadmilling of the bacterial tubulin FtsZ organizes septal cell wall synthesis. *Science (1979).* **355**, 744–747 (2017).

11. Montecinos-Franjola, F., Bauer, B. L., Mears, J. A. & Ramachandran, R. GFP fluorescence tagging alters dynamin-related protein 1 oligomerization dynamics and creates disassembly-refractory puncta to mediate mitochondrial fission. *Scientific Reports 2020 10:1* **10**, 14777- (2020).

12. Friedman, J. R. *et al.* ER tubules mark sites of mitochondrial division. *Science (1979).* **334**, 358–362 (2011).

13. Korobova, F., Ramabhadran, V. & Higgs, H. N. An actin-dependent step in mitochondrial fission mediated by the ER-associated formin INF2. *Science (1979).* **339**, 464–467 (2013).

14. Bustillo-Zabalbeitia, I. *et al.* Specific Interaction with Cardiolipin Triggers Functional Activation of Dynamin-Related Protein 1. *PLoS One* **9**, e102738 (2014).

15. Zaghbani, S., Pranti, R. K., Faber, L. & Garcia-Saez, A. J. MitoSkel: AI tool for semantic segmentation and quantification of mitochondria from light microscopy images. *Biomed. Signal Process. Control* **106**, 107762 (2025).
